# Gene network analysis to determine the effect of hypoxia-associated genes on brain damages and tumorigenesis using an avian model

**DOI:** 10.1186/s43141-021-00184-5

**Published:** 2021-07-08

**Authors:** Hamed Kharrati-Koopaee, Esmaeil Ebrahimie, Mohammad Dadpasand, Ali Niazi, Rugang Tian, Ali Esmailizadeh

**Affiliations:** 1 Institute of Biotechnology, Shiraz University, Shiraz, Iran; 2 Department of Animal Science, Faculty of Agriculture, Shahid Bahonar University of Kerman, Kerman, Iran; 3 School of Animal and Veterinary Sciences, The University of Adelaide, Adelaide, Australia; 4 Genomics Research Platform, School of Life Sciences, La Trobe University, Melbourne, Victoria Australia; 5 Department of Animal Science, School of Agriculture, Shiraz University, Shiraz, Iran; 6 Institute of Animal Husbandry, Inner Mongolia Academy of Agricultural & Animal Husbandry Sciences, Hohhot, 010031 China

**Keywords:** Hypoxia, Brain damages, Gene network, Tumorigenesis

## Abstract

**Background:**

Hypoxia refers to the condition of low oxygen pressure in the atmosphere and characterization of response to hypoxia as a biological complex puzzle, is challenging. Previously, we carried out a comparative genomic study by whole genome resequencing of highland and lowland Iranian native chickens to identify genomic variants associated with hypoxia conditions. Based on our previous findings, we used chicken as a model and the identified hypoxia-associated genes were converted to human’s orthologs genes to construct the informative gene network. The main goal of this study was to visualize the features of diseases due to hypoxia-associated genes by gene network analysis.

**Results:**

It was found that hypoxia-associated genes contained several gene networks of disorders such as Parkinson, Alzheimer, cardiomyopathy, drug toxicity, and cancers. We found that biological pathways are involved in mitochondrion dysfunctions including peroxynitrous acid production denoted in brain injuries. Lewy body and neuromelanin were reported as key symptoms in Parkinson disease. Furthermore, calmodulin, and amyloid precursor protein were detected as leader proteins in Alzheimer’s diseases. Dexamethasone was reported as the candidate toxic drug under the hypoxia condition that implicates diabetes, osteoporosis, and neurotoxicity. Our results suggested DNA damages caused by the high doses of UV radiation in high-altitude conditions, were associated with breast cancer, ovarian cancer, and colorectal cancer.

**Conclusions:**

Our results showed that hypoxia-associated genes were enriched in several gene networks of disorders including Parkinson, Alzheimer, cardiomyopathy, drug toxicity, and different types of cancers. Furthermore, we suggested, UV radiation and low oxygen conditions in high-altitude regions may be responsible for the variety of human diseases.

**Supplementary Information:**

The online version contains supplementary material available at 10.1186/s43141-021-00184-5.

## Background

Hypoxia refers to the condition of low oxygen pressure in atmosphere, and is also considered as one of the most important factors that can impact on many biological pathways of cells [52]. Thus, organisms must adapt to the low oxygen environment to increase the chance of survival [66]. It has been demonstrated that there is close association between abnormal level of oxygen and human’s disorders and hypoxia is known as the common symptom among human diseases such as cancers, cardiovascular, heart failure, ischemia, cerebral edema, and diabetes [26, 71, 82, 110, 111, 119].

Many studies aimed to explain the role of hypoxia in diseases and disorders. As an example, in high-altitude conditions, high doses of UV (ultraviolet) radiation lead to the *DNA* damage, cell apoptosis, skin cancer, and tissue injuries in mammals [26, 71, 82, 97, 108, 119]. In addition, the low oxygen concentration in high-altitude conditions is another risk factor for diseases [70]. The lack of normal oxygen level leads to the inadequate mitochondrial metabolism, and also plays a critical role in cell survival [41]. Therefore, any alteration or dysfunction in mitochondrial activity can lead to irreparable injuries of the body’s organs, especially in the central nervous system [15], because the brain is considered as the most sensitive organ to the consumption of oxygen and energy.

In this way, cerebral anoxia induces the neuron cell death and apoptosis, which will lead to hypoxic brain injury including Alzheimer, Huntington, and Parkinson [100, 116]. It should be noted that numerous investigations have attempted to identify candidate genes and molecular mechanisms involved in the process of adaptation to hypoxia and incidence of disorders; however, this process contains complex biological regulatory pathways, leader proteins, and different gene expression patterns [53]. Therefore, there is no evidence for integrated response to hypoxic stress in humans [41].

The network analysis of the hypoxia-regulated proteins can provide new insight for a better understanding of the molecular mechanisms of the adaptation to anaerobic stress and predict roles of key proteins in human diseases [52].

Animal models have been widely used in order to explore diseases and provide novel insights into the mechanisms of human diseases. Although biological pathways of animals do not conform to humans perfectly, many treatment systems and drugs were developed using animal models [16].

Many of the features of avian biology and organization of the chicken genome make it an ideal model organism for phylogenetics and embryology, along with applications in agriculture and medicine [11, 25, 28]. Since the sequencing of the genome and the development of high-throughput tools for the exploration of functional elements of the genome, the chicken has reached model organism status. Functional genomics and computational analyses in chickens provide powerful tools in order to understand the function and regulation of genes and obtaining new insight into the evolution of gene families in birds and other organisms [18].

In this study, based on the chicken hypoxia-associated genes involved in response to hypoxia [51] the biological network analysis was investigated to clarify the roles of key candidate genes in the human hypoxia-related diseases.

## Methods

The current study was founded based on the outcomes of our pervious investigation which were planned to identified hypoxia-associated genes in native chickens. Previously, we carried out a comparative population genomic study by whole genome resequencing of highland and lowland Iranian native chickens to identify genomic variants associated with hypoxia conditions [51]. In this way, 80 hypoxia-associated candidate genes were obtained for gene network analysis. Here, to avoid repetition, the summary of material and method was described. Supplementary file S1 provides the complete details of sampling and analyzing data.

### Blood sampling, whole genome sequencing, and phenotypic data

Briefly, blood samples were collected from ten native chickens in Isfahan (highland, altitude = 2087 m, five samples) and Sari (lowland, altitude = 54 m, five samples) provinces in Iran. Total genomic *DNA* was isolated from the whole blood and whole genome was sequenced using Hiseq2000 platform and the length of the provided paired-end short reads was 125 bp. The raw genome resequencing reads were deposited in the browser of chickenSD database (http://bigd.big.ac.cn/chickensd/). In order to classify the highland and lowland chickens phenotypically, 24 quantitative traits were recorded for principle component analysis (PCA) and discriminate analysis in Minitab software (version: 17). The summary of recorded traits was included body weight (gr), neck length, body size (between waist and pectoral circumference), shank length, body size (between waist and abdominal circumference), wing length, tail length, femur length, crown length, crown height, back cape length, body size (between pectoral and cloaca circumference), head height (height from head to floor), femur diameter and shank diameter. To reduce measurement error, all chickens were matured while recording. It should be noted that the same recording protocol was also used and all chickens were matured while recording. Supplementary file S1 gives more detail about phenotypic data analysis.

### Measurement of UV radiation and atmosphere pressure

Atmosphere pressure and UV dose are different in highland and lowland regions. The measurement of UV dose is associated with environmental conditions such as earth-to-sun distance, absorption by atmospheric gases, air pollutions, and also the amount of atmosphere pressure depends on temperature and humidity [50, 85]. Therefore, in order to show the UV dose and atmosphere pressure in selected highland and lowland regions, the recent annual average was reported for Isfahan (highland; UV: 3228.83 ± 51.12 (j/m^2^), atmosphere pressure: 760.63 ± 12.19 (mmHg)) and Sari (lowland; UV: 2569.60 ± 41.87 (j/m^2^), atmosphere pressure: 1015.12 ± 16.74 (mmHg)) (www.weather.ir).

### Quality control, trimming, and mapping

The function of quality control in CLC Genomic Workbench (8.5.1) was applied by the following parameters for each sample: length distribution, GC content, ambiguous base content, Phred score, nucleotide contribution, enrich 5 mers, and duplicate sequences [22]. The adaptor sequences were removed by Illumina Company; thereby, trimming was carried out based on other parameters. The reference genome and annotations were downloaded from the Ensembl database. Annotations included gene annotations and variants (ftp://ftp.ensembl.org/pub/relase-84/fasta/gallus_gallus). Mapping was performed against the reference genome. Briefly, mapping parameters were included mismatch cost = 2, insertion cost = 3, deletion cost = 3, length fraction = 0.7, similarity fraction = 0.8 [69]. More details were provided in supplementary file S1.

### Genetic variants detection and gene ontology enrichment analysis

The variant detection algorithm was run by CLC genomics workbench (8.5.1). The ploidy level was fixed in chickens (2n = 78). Therefore, fixed ploidy algorithm was applied for variant calling based on the following parameters: minimum count = 2, minimum frequency (%) = 30, base quality filter = yes, neighborhood radius = 15, minimum central quality = 30, minimum neighborhood quality = 25 [22, 23].

**Fig. 1:**
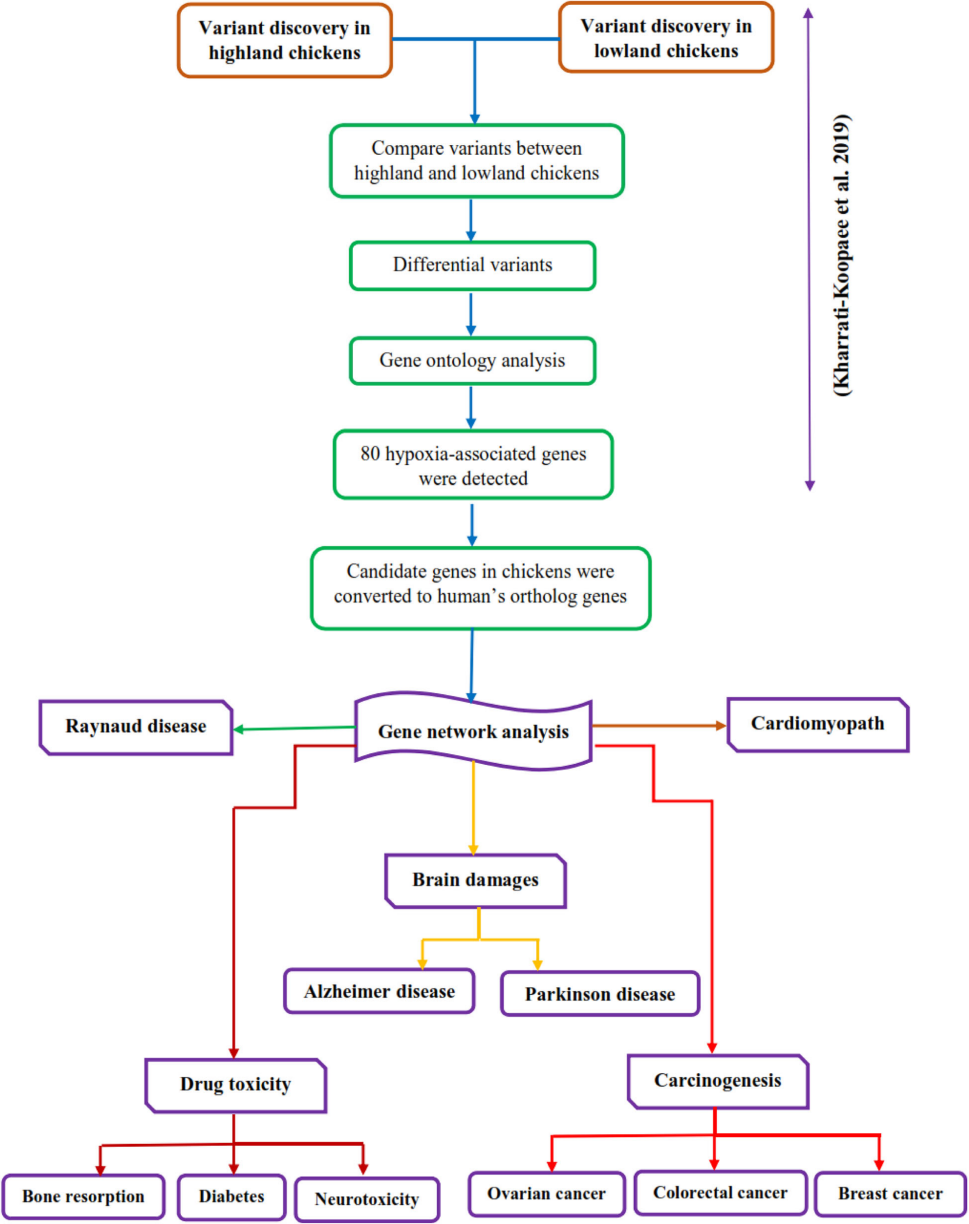
The workflow of the variant detection and gene network analyses which are associated with human diseases such as brain damages, carcinogenesis, cardiomyopathy, and drug toxicity

After variants calling, variations of highland chickens were compared to the reads of lowland chickens as a control tool in order to remove the common variation between lowland and highland samples. The file of gene ontology (GO) association, which included gene names and associated gene ontology terms, was downloaded from the gene ontology consortium (http://geneontology.org/) and imported to CLC Genomic Workbench (8.5.1). The output of amino acid change analysis was applied in order to analyze the gene ontology enrichment of the biological process, molecular function, and cellular component. The significance level of GO analysis was determined to be 0.01. Finally, based on the results of GO analysis, candidate genes associated with significant GO term (*P* ≤ 0.01) were used in the gene network analysis [51]. The lists of selected genes are presented in Table S1. The graphic abstract of genetic variants detection and gene network analyses are briefly depicted in Fig. 1.

### Gene network analysis

Gene network analysis was performed based on hypoxia-associated candidate genes which were reported by comparative genomic study between highland and lowland chickens [51]. Unlike, chicken’s genes a lot of information of human’s genes are available in the database of Studio pathway software web (Elsevier). Therefore, the identified chicken Ensembl IDs were converted to human orthologs by BioMart tool in Ensembl database (https://asia.ensembl.org/) in order to build an informative gene network (Table S1) [22]. BioMart tool provides an easy-to-use web-based tool that allows extraction of data without any programming knowledge or understanding of the underlying database structure. Gene network analysis was carried out by Pathway Studio web (Elsevier), with the purpose of identifying gene network and upstream neighbors network for diseases based on parameters of Min overlap = 2, and *P* value < 0.05 [75].

## Results

The complete results of genetic variants detection that were published in our investigation [51] are provided in supplementary file S1. However, the summary outcomes of the previous study describe here in order to clarify the identification of hypoxia-associated genes.

A total of more than 20 million variations were generated, including single nucleotide variant (SNV), multi nucleotide variant (MNV), insertion, deletion, and replacement. In order to remove the common variants between highland and lowland chickens, total variants of those samples were compared. A total of 97,610 and 17,024 genetic variants were detected for both males and females as the differential variants between highland and lowland chickens, respectively. The results of gene ontology analysis indicated that the most frequent significant GO terms belonged to DNA repair, histone binding, pericardium morphogenesis, thalamus development, and immune response. Finally, 80 candidate genes, associated with significant GO terms (*P* ≤ 0.01) were selected for gene network analysis (Table S1).

The outcomes phenotypic data analysis indicated that lowland and highland chickens can be classified into two separated groups, phenotypically. The accuracy was estimated to be 75% (Tables S2-S3).

### Gene network analysis

The nomenclature of network figures is given in Table 1, and the summary results of gene network building are shown in Table 2.

**Table 1:**
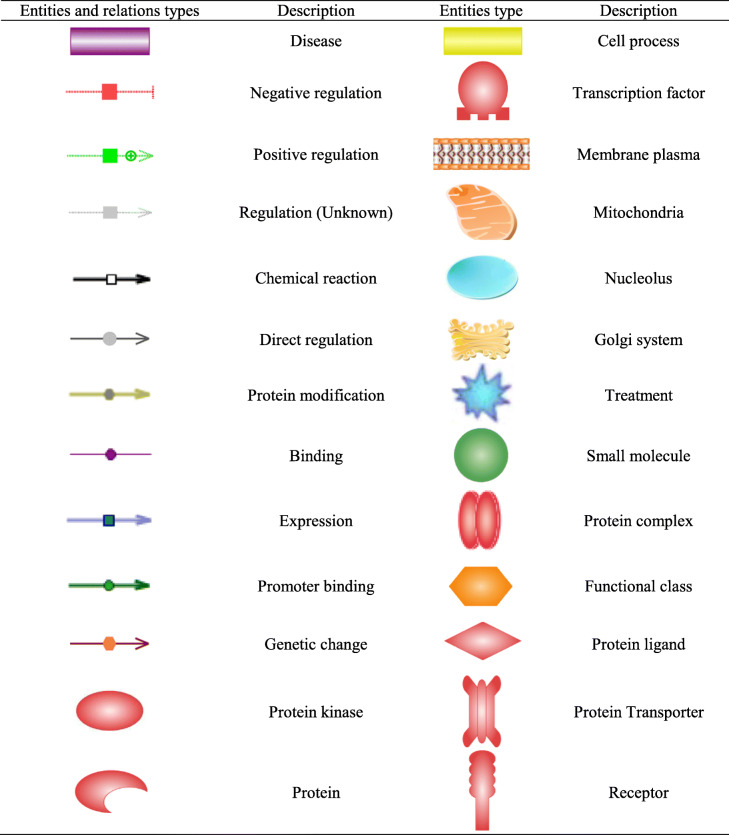
The legend of entities and relations types for gene network analysis

**Table 2:** The details of significant gene networks of diseases which were constructed by hypoxia-associated genes

	Disorder	Total genes in database	Total genes in network	Enriched genes in network	Gene names	*P* value
**Brain damages**	Parkinson disease	104	14	9	*CYCS*, *BID*, *GPX1*, *OGDH*, *TYR*, *SOD2*, *SLC18A2*, *XDH*, *NQO1*	*
	Mitochondrial damage	117	8	7	*HIF1A*, *NOS1*, *NOS2*, *SLC9A1*, *SLC8A1*, *SOD2*, *PARP1*	**
	Alzheimer disease	93	16	9	*CYCS*, *BAG1*, *SLC8B1*, *MICU1*, *MCU*, *DNM1*, *OPA1*, *DNM1L*, *CASP3*	*
**Drug toxicity (Dexamethasone)**	Diabetes	48	21	15	*SGK1*, *NR3C1*, *EP300*, *RUNX2*, *SOST*, *DKK1*, *LRP6*, *LRP5*, *DVL1*, *GSK3B*, *IRS1*, *IRS2*, *PDPK1*, *AKT1*, *TBC1D4*	*
	Neurotoxicity	45	17	17	*SGK1*, *NR3C1*, *EP300*, *RUNX2*, *SOST*, *DKK1*, *LRP6*, *LRP5*, *DVL1*, *GSK3B*, *IRS1*, *IRS2*, *MAPT*, *PDPK1*, *AKT1*, *SIRT2*, *TBC1D4*	**
	Bone resorption	141	20	16	*SGK1*, *NR3C1*, *EP300*, *RUNX2*, *SOST*, *DKK1*, *LRP6*, *LRP5*, *DVL1*, *AKT1*, *TGFBR1*, *CTNNB1*, *BGLAP*, *TNSFS11*, *CSF1*, *TNFRSF11B*	**
**Carcinogenesis**	Ovarian cancer	698	9	8	*VEGFC*, *LRP1B*, *ATR*, *RIF1*, *HDAC10*, *UIMC1*, *BARD1*, *LRPPRC*	**
	Colorectal cancer	1067	11	9	*VEGFC*, *ATR*, *LRP1B*, *CA4*, *CD109*, *AKAP12*, *MKI67*, *DOT1L*, *CENPF*	**
	Breast cancer	1660	21	18	*VEGFC*, *ATR*, *LRP1B*, *LRP6*, *GPNMB*, *APOB*, *GAS8*, *CCDC170*, *ATAD2*, *POLQ*, *SLX4*, *USP1*, *SYNE2*, *BARD1*, *BMX*, *UIMC1*, *CENPF,USP1*	**
	Metastasis	5278	7	6	*ATAD2*, *BMX*, *BDKRB2*, *PRKDC*, *SYTL2*, *CD200*	**
	Cardiomyopathy	330	7	6	*XIRP1*, *ALPK3*, *BDKRB2*, *SYNE2*, *LRPPRC*, *APOB,*	**
	Raynaud disease	36	5	3	*DDAH1*, *ARG2*, *RHOA*	*

*P < 0.05, **P < 0.01

Table 2 shows results of gene network construction by hypoxia-associated genes. “Total gene in database” indicates the count of all available candidate genes for diseases in the database of Pathway Studio software. As an example, 104 candidate genes are available in the database of Pathway Studio for Parkinson disease. “Total genes in network” shows the count of genes that were constructed the network. For instance, 14 candidate genes contributed to network building for Parkinson disease. In the current study, 80 hypoxia-associated genes were used for network analysis; therefore, “Enriched genes in network” shows how many of 80 hypoxia-associated genes were enriched in network. For Parkinson disease, 9 candidate genes which we reported as hypoxia-associated genes were enriched in the network.

### Brain damages

Hypoxic brain injuries occur when the brain receives less oxygen than its requirements. Generally, the brain needs the continuous supply of oxygen for its survival and uses 20% of the body’s oxygen intake. Mitochondrion plays an important role in cell respiration and ATP production, while there is a close relationship between mitochondrial oxygen consumptions, neuronal activity, and aerobic metabolisms. Therefore, any interruption of cell respiration can lead to brain injuries [48].

**Fig. 2:**
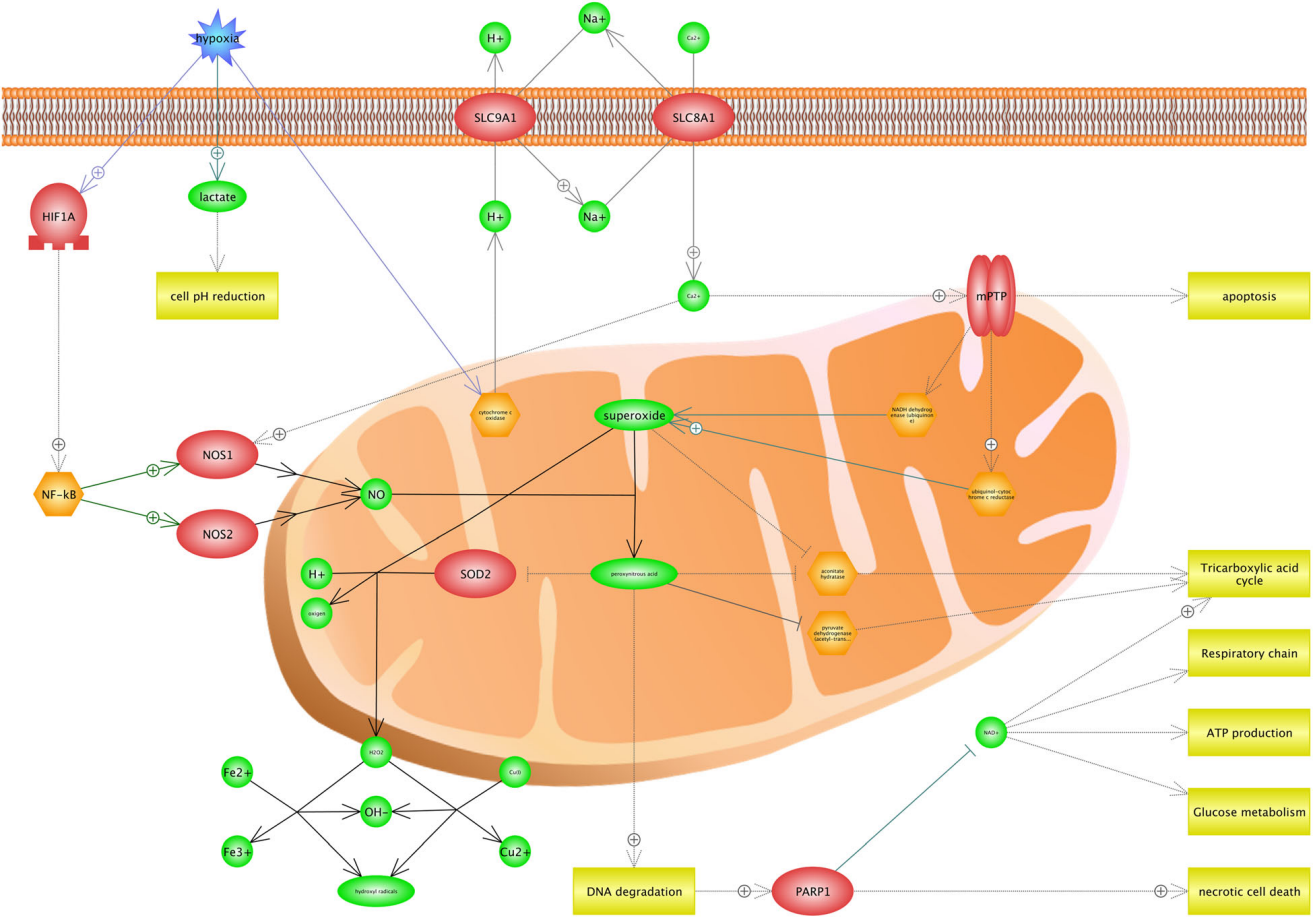
The effect of hypoxia on mitochondrial damage. Hypoxia reduces the level of pH in cells, which will result in the increased lactate. The process of transferring that exists in plasma membrane depends on the cellular pH. Thus, the level of Ca^2+^ increases in the cytoplasm. The high concentration of Ca^2+^ contributes in two processes of the cell. First, it enhances *MPTP* complex (mitochondrial permeability transition pore) and leads to the ATP depletion, mitochondria damage, and apoptosis. Additionally, *MPTP* has a regulatory role in superoxide and peroxynitrous acid productions and DNA degradation. Second, Ca^2+^ contributes in the peroxynitrous acid production of superoxide, indirectly. This figure shows the important role of Peroxynitrous acid in necrotic cell death by several pathways. Peroxynitrous acid can enter the nucleus and leads to the DNA damage and mutations. DNA degradation is, directly and indirectly, responsible for several varieties of cell processes including necrotic cell death, glucose metabolism, and ATP production

Our findings suggested that there was an association between hypoxia and mitochondrion functions such as glucose metabolism, ATP production, respiratory chain, and Tricarboxylic acid cycle (Fig. 2). Hypoxia, which is considered as an environmental stress, can reduce the cells’ pH level; thus, it will lead to the increased lactate. More importantly, it is proved that the transports process in plasma membrane depends on cellular pH. Therefore, the level of Ca^2+^ increases in cytoplasm. Figure 2 shows that Ca^2+^ can lead two processes in the cell. First, it enhances MPTP complex (mitochondrial permeability transition pore) and causes the ATP depletion, mitochondria damage, and apoptosis. Additionally, it was found that MPTP has a regulatory role in ubiquinol-cytochrome c reductase and NADH dehydrogenase activities, and leads to *DNA* degradation which itself results in the superoxide and peroxynitrous acid productions. Second, Ca^2+^ has a positive effect on NOS1 (Nitric Oxide Synthase 1) and contributes in peroxynitrous acid production from superoxide, indirectly. We found that hypoxia induces the HIF1A (hypoxia-inducible factor 1-alpha) transcription factor and also contributes in the peroxynitrous acid production by activating NOS1 and NOS2 (Nitric Oxide Synthase 2), indirectly. It is observed that hypoxia involves in the process of producing peroxynitrous acid and necrotic cell death by applying several approaches (Fig. 2). Peroxynitrous acid is known as a potent oxidant that reacts with many macro molecules including proteins and lipids. Moreover, peroxynitrous acid can enter the nucleus and cause the *DNA* damage and mutations [36]. *DNA* degradation is both directly and indirectly responsible for several varieties of cell processes including necrotic cell death, glucose metabolism, and ATP production. We also found that hydroxyl radicals are generated in cell by the key roles of peroxynitrous acid, superoxide, and SOD2 proteins. It was observed that hydroxyl radicals indicate the apoptosis and cell damages [88].

#### Parkinson disease

**Fig. 3:**
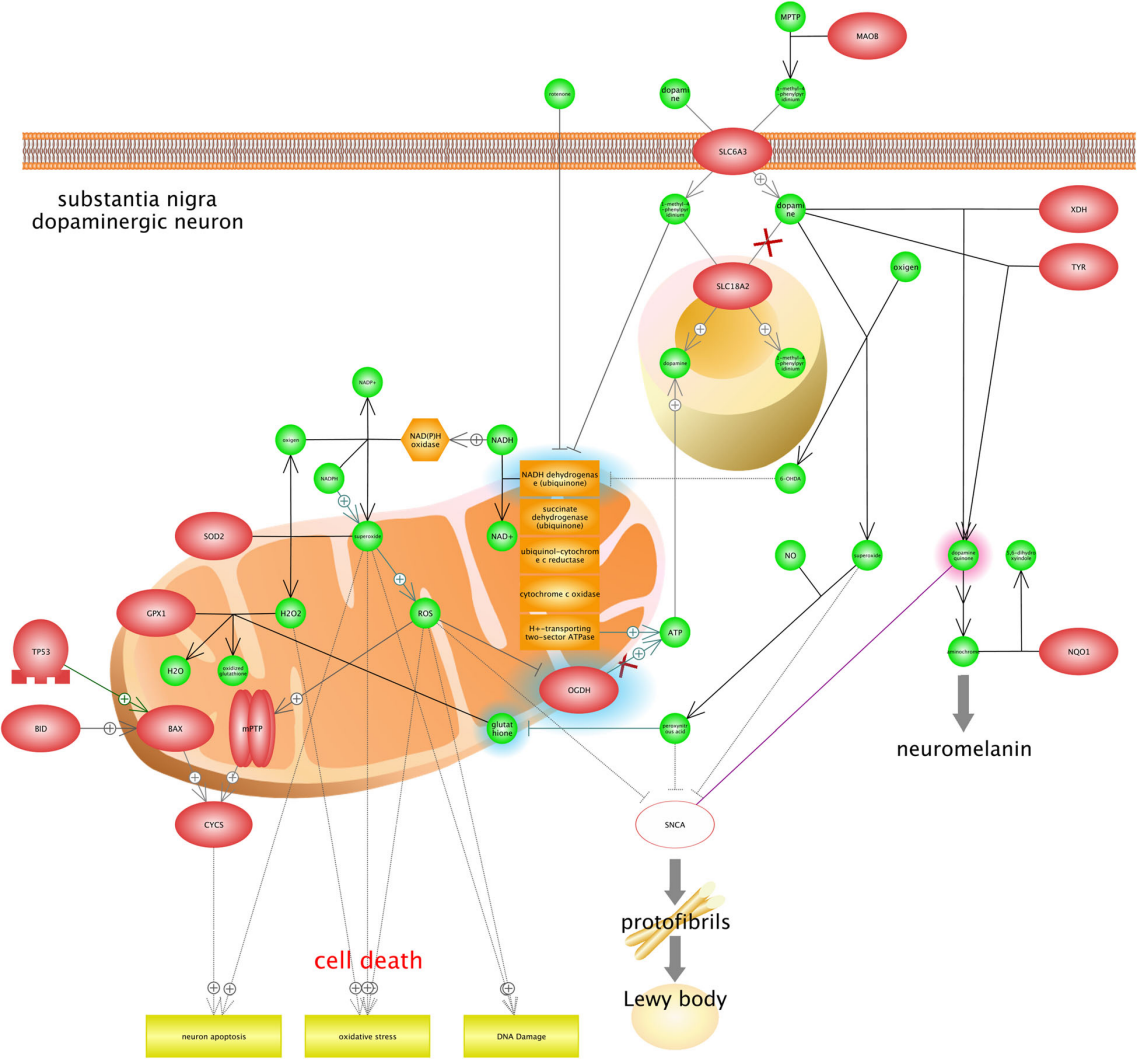
The role of hypoxia in Parkinson’s disease by neuron apoptosis, DNA damage, and oxidative stress. Results illustrated that Substantia nigra is the most important part of the midbrain that plays a critical role in the development of Parkinson's disease (PD). In Substantia nigra, Superoxide contributes in the neuron apoptosis, and Superoxide can also lead to oxidative stress and DNA damage by increasing ROS (Reactive oxygen species). Results show that *CYCS* (cytochrome c) protein can lead to the neuron apoptosis. In fact, it has an important role as the electron carrier between the cytochrome c1 subunit of cytochrome reductase and cytochrome oxidase complex, but releasing cytochrome c into the cytosol can activate the apoptosis process in cells. Lewy bodies and Neuromelanin are found as the main symptoms of PD. Lewy bodies are associated with unusual deposits of proteins in Substantia nigra, and Neuromelanins are also considered as complex structures containing granules of melanin polymer. In addition, loss of neuromelanin can be considered as effective symptoms of PD

Parkinson disease (PD) is defined as a neurodegenerative disorder impacts on movements. It is recognized that the incidence of PD has overshadowed; however, several risk factors including age, family history, pesticide exposure, and environmental chemicals were suggested for PD [4]. Substantia nigra, as a long nucleus, is the most important part of the midbrain that plays a critical role in movements of the body. Figure 3 shows that superoxide contributes in neuron apoptosis in the Substantia nigra; it is also found that the Superoxide can lead to oxidative stress and *DNA* damage by increasing ROS (Reactive oxygen species). Results show that ROS are known as normal forms of oxygen in the process of metabolism; however, the abnormal levels of ROS contribute in the cell damage. Figure 3 illustrates that the CYCS protein can lead to neuron apoptosis. *CYCS* gene encodes the cytochrome c, which is a member of the electron transport chain in the mitochondrion. Cytochrome c, which is recognized as the electron carrier between the cytochrome c1 subunit of cytochrome reductase and cytochrome oxidase complex, has a very significant role; however, releasing cytochrome c into the cytosol can lead to the activation of the apoptosis process in the cell (UniProtKB - P99999). Lewy bodies are well-known as the main symptom of PD [109]. Lewy bodies are associated with unusual deposits of proteins in Substantia nigra. It is believed that α-synuclein (α-Syn or SNCA) contributes in the lewy body formation and clumps of α-Syn also have a significant role in PD. We found that neuromelanin is considered as another important marker of PD (Fig. 3). Neuromelanins are known as complex structures that contain granules of melanin polymer; however, they are actually dark pigments that are deposited with aging in substantia nigra [91]. It is reported that the loss of Neuromelanin could be considered as an effective symptom of PD. Interestingly, it is suggested that there is a close association between the degeneration of dopamine neuron and high amounts of Neuromelanin. Dopaminergic neurons are recognized as the most significant sources of dopamine in brain, and their dysfunctions are also associated with different types of neurological disorders, especially PD [2, 37, 120]. We also found that rotenone inhibits mitochondrion respiratory chain complex, and induces cell damages. Rotenone is known as a natural plant toxin produced by tropical plants and has been used for more than 150 years in the world because of its insecticide properties. It is proved that levels of rotenone toxicity vary in different animals. For example, it is highly toxicant for aquatic organisms, while has a low degree of toxicity for birds and mammals [61]. It is recognized that rotenone, which is considered as an oxygen sensor, is a key component of hypoxia sensing [103].

#### Alzheimer disease

**Fig. 4:**
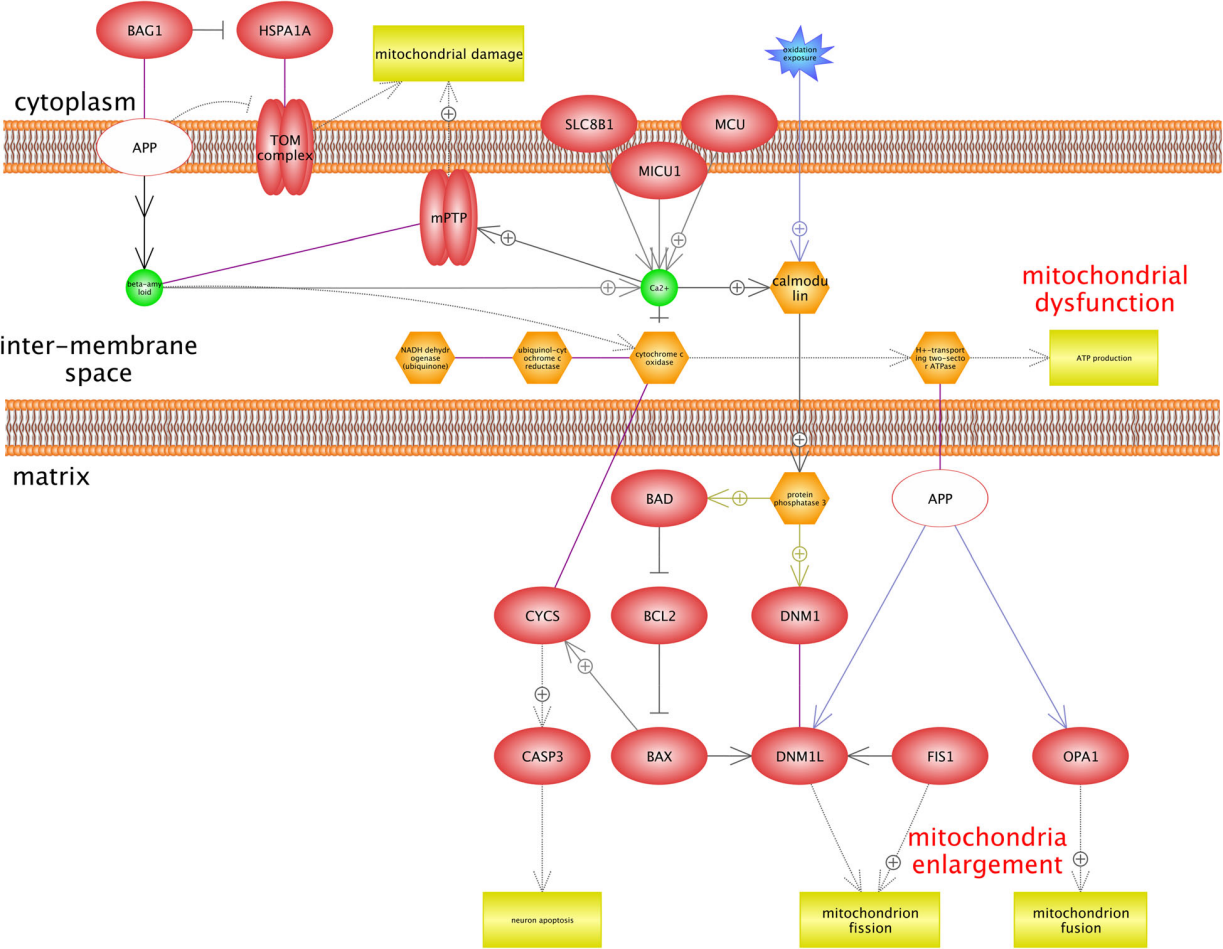
Gene network analysis to identify the roles of hypoxia-associated genes in Alzheimer’s disease (AD). This figure shows two main protein complexes including TOM (translocase of the outer mitochondrial membrane) and MPTP (mitochondrial permeability transition pore) that play a critical role in AD. Most of the proteins of mitochondrion are produced in the cytoplasm, and then, they are imported to the mitochondrion by the TOM complex. However, APP (amyloid precursor protein) inhibits the TOM complex and leads to the mitochondrial damage. The accumulation of β-amyloid peptide (Aβ) is recognized as one of the main features of AD. Mitochondrial calcium is overloaded by Aβ and MCU (mitochondrial calcium uniporter). Based on our results, there is an association between mitochondrial calcium overloads that induces the activity of MPTP, which is related to mitochondrial dysfunction because it is opened in the inner mitochondrial membrane. It is recognized that protons can cross into the mitochondria and lead to the ATP depletion and mitochondria damage

Alzheimer’s disease (AD) is related to the central nervous system that impacts on the memory and leads to the cognitive decline [27, 92]. Results indicated that there are two main complexes including TOM (translocase of the outer mitochondrial membrane) and MPTP, which play a critical role in AD (Fig. 4). TOM complex is well-known as the protein translocator of the outer membrane of mitochondrion. Most of the mitochondrion proteins are produced in the cytoplasm and are imported to the mitochondrion by the TOM complex [39]. Figure 4 shows that APP (amyloid precursor protein) inhibits the TOM complex and leads to the mitochondrial damage. We found that there are associations between APP, OPA1, DNM1L, which contribute in the mitochondrion fusion and fission. However, their accurate role in mitochondrial dynamics remains to be determined [40]. It is believed that mitochondrion is dynamic organelle in cells and is also able of fission/fusion. In fact, fission/fusion is related to the quality control of mitochondrion. Therefore, it is recognized that any alteration in mitochondrial fusion/fission and related proteins contribute in AD and PD disorders [1, 44]. The accumulation of β-amyloid peptide (Aβ) is considered as one of the hallmark features of AD, and is produced by the degeneration of protein tau; however, it contributes in the process of regulating synaptic scaling and synaptic vesicle release, physiologically [73]. Based on our results mitochondrial calcium is overloaded by Aβ and MCU (mitochondrial calcium uniporter). MCU is located on mitochondrial inner membrane and involves the calcium uptake (Uniprot: Q8NE86). According to Fig. 4, there is an association between mitochondrial calcium overload and MPTP activity. MPTP is related to mitochondrial dysfunction because it is opened in the inner mitochondrial membrane; we also found that molecules of < 1.5 KDa, including protons, could be transferred to mitochondria and lead to the ATP depletion and mitochondria damage in this situation [38]. Calmodulin is known as a calcium-binding protein involves in the process of regulating different protein targets’ multitude; therefore, it impacts on many cellular functions. Calmodulin is considered as a specific biomarker in AD; thus, blood’s Calmodulin levels are increased significantly in AD cells, but it is not reported in other neurodegenerative disorders [29]. Additionally, it is demonstrated that calmodulin-binding proteins play a significant role in the formation of Aβ [72].

### Drug toxicity

**Fig. 5:**
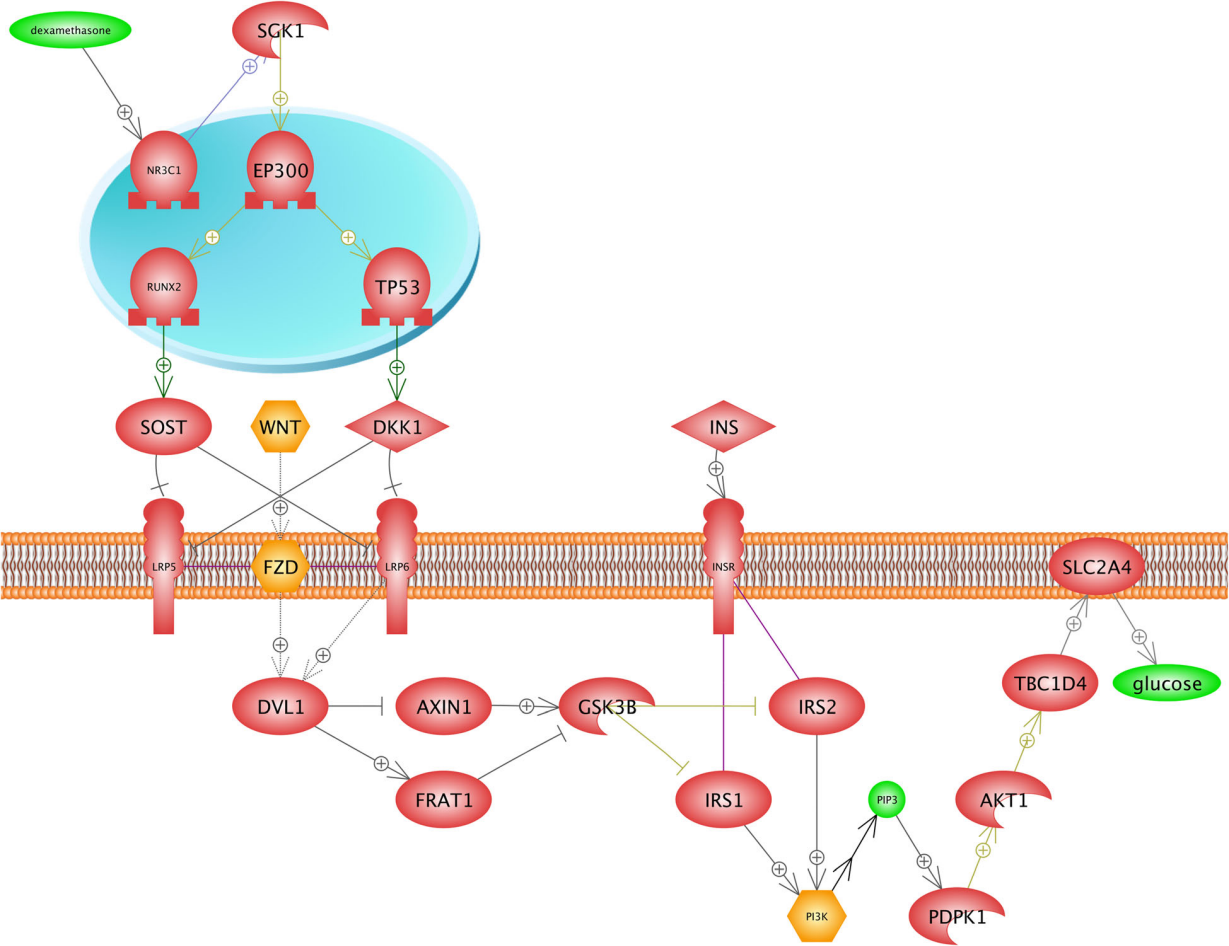
The role of dexamethasone in the process of inducing diabetes in hypoxia condition. This figure illustrates that the *NR3C1* gene activates *SGK1* (Serine/threonine-protein kinase) and other TFs block two receptors (*LRP6* and *LRP5*) in the cell membrane. In fact, *SGK1* gene is well known as the regulator for many biological pathways such as TFs, receptors, and transporters. Thus, WNT-FZD (Wingless/Int1-Frizzled) and *LRP6* activate protein kinase *GSK38*. We found that it has a positive effect on the *SLC2A4* gene, indirectly. It is proved that the *SLC2A4* gene encodes the glucose transporters family (GLUTs) in diabetes, which is considered as an insulin-sensitive transporter. GLUTs contribute in glucose hemostasis and diabetes diseases

**Fig. 6:**
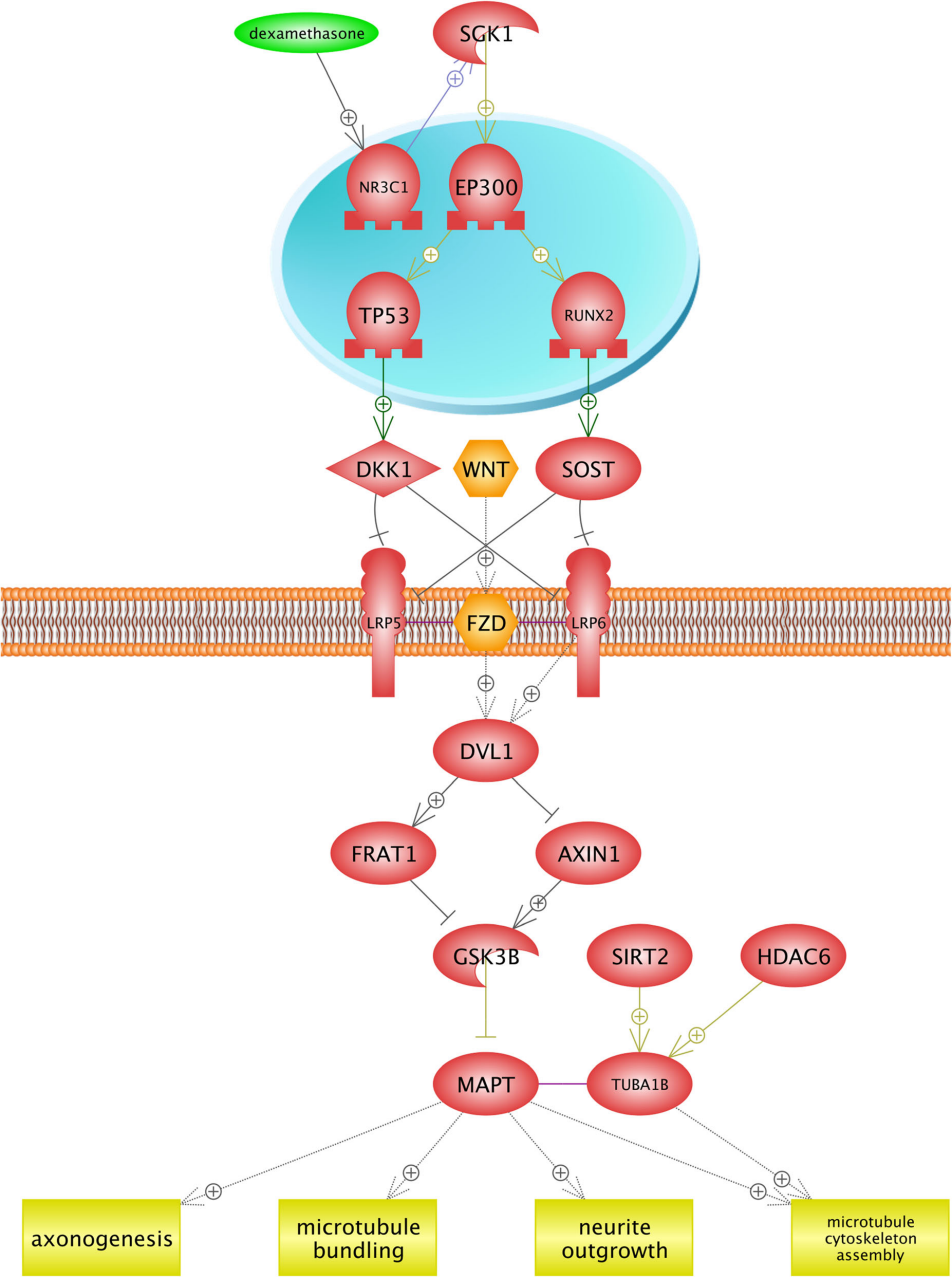
The development of neurotoxicity process in hypoxia condition by drug toxicity of Dexamethasone. Neurotoxicity occurs when neurotoxins affect the normal activity of the nervous system and ultimately, damage the nervous tissue. In the other words, all biological processes that lead to the development of the nervous system are inhibited. Generally, the function of the nervous system depends on neurite (axons and dendrites) outgrowth. In neurotoxicity, the number and length of neurites and axonogenes are decreased. MAPT provides microtubule-associated protein tau found in the nervous system and contributes in the process of stabilizing microtubules and structures the cytoskeleton. Figure 6 suggests that dexamethasone inhibits MAPT gene and decreases the growth of neurons in the nervous system and leads to neurotoxicity

**Fig. 7:**
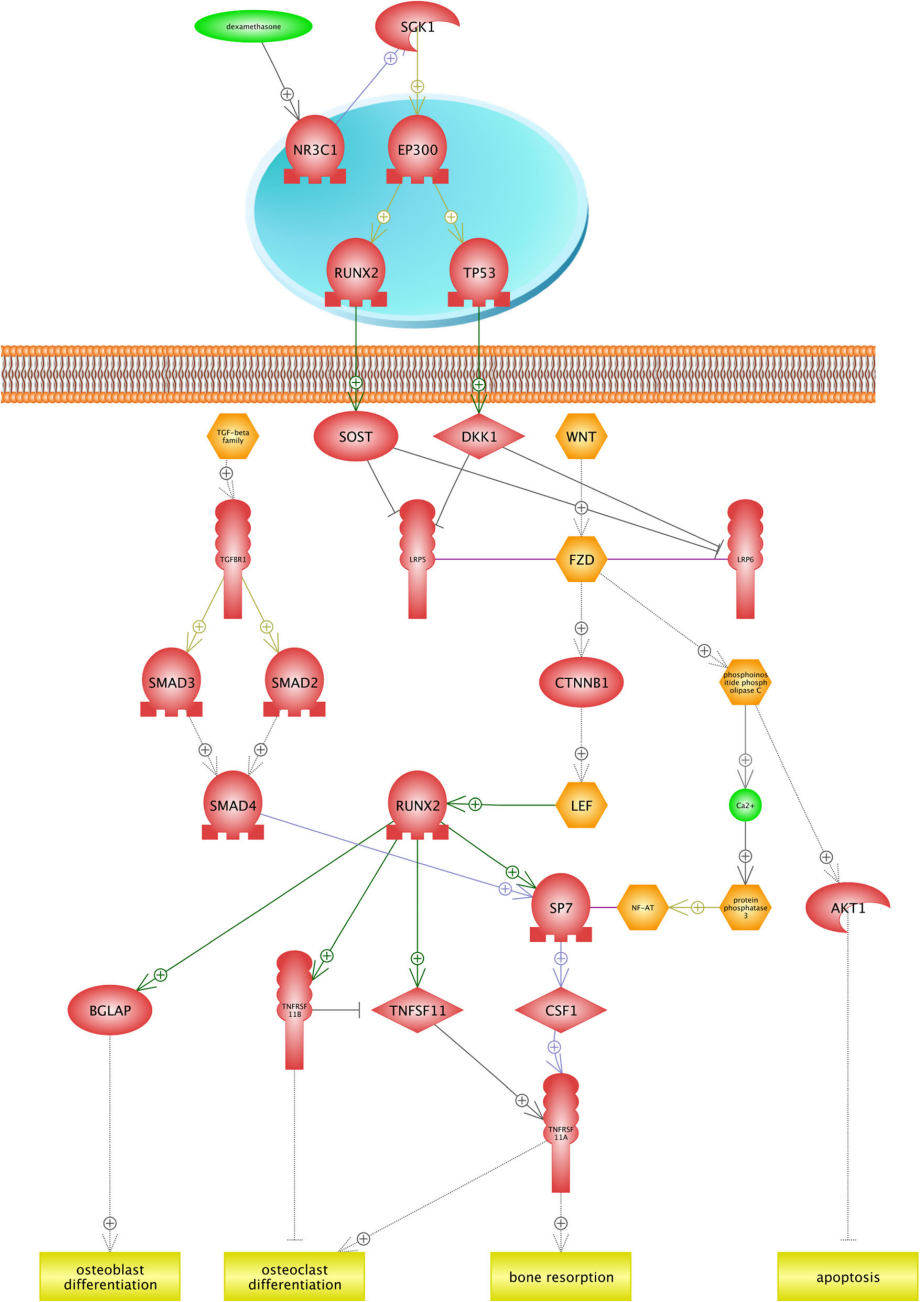
The association between drug toxicity of dexamethasone and bone resorption in low oxygen conditions. Bone resorption is known as another side effect of dexamethasone. According to our findings, the *RUNX2* (Runt-related transcription factor 2) gene is associated with the skeletal morphogenesis by activating the tumor necrosis factor receptor superfamily (*TNFRSF*) that inhibits the osteoclast differentiation. Additionally, *RUNX2* transcription factor induces tumor necrosis factor superfamily (*TNFSF*) ligand and bone resorption. *TNF/TNFR* proteins are classified as the cytokine, and it is also demonstrated that cytokines might contribute in bone resorptions directly by increasing proliferation and activity of cells in the osteoclast lineage. *TNFRSF11A* gene encodes osteoprotegerin (OPG) protein, which is also known as osteoclastogenesis inhibitory factor (OCIF)

Many pathways of drug metabolisms such as oxidation, sulfation, and acetylation depend on the oxygen availability; thereby, the investigation of drugs and their metabolism pathways can provide beneficial information about optimal drug therapy and avoidance of drugs’ side effects in plateau regions [57]. Results of this study showed that Dexamethasone under hypoxia condition induces diabetes, osteoporosis, and neurotoxicity. Dexamethasone is a corticosteroid that prevents the release of substances in the body that cause inflammation. We found that similar networks were constructed for diabetes and neurotoxicity (Figs. 5 and 6). In the first step, dexamethasone induces the transcription factor (TF) NR3C1 (Nuclear Receptor Subfamily 3 group C member 1). It has been demonstrated that the *NR3C1* gene plays two critical roles. First, it encodes glucocorticoid receptor as a transcription factor that binds to glucocorticoid response elements in the promoters of glucocorticoid responsive genes in order to activate their transcription. Second, it is known as the regulator of other transcription factors [32]. We found that the *NR3C1* gene blocks two receptors (*LRP6* and *LRP5*) placed in the cell membrane by activating SGK1 (Serine/threonine-protein kinase) and other TFs. In fact, the *SGK1* gene is well known to be a regulator of many biological pathways including TFs, receptors, proliferation, cellular enzymes, and transporters (Uniprot: O00141). Finally, WNT-FZD (Wingless/Int1-Frizzled), and LRP6 activate the protein kinase GSK38. These described pathways are common between diabetes and neurotoxicity. In diabetes (Fig. 5), the *SLC2A4* gene encodes glucose transporters family (GLUTs), which is known as an insulin-sensitive transporter. GLUTs contribute in glucose hemostasis and diabetes diseases [99]. Figure 6 illustrates that the development of the neurotoxicity process is related to axonogenesis, neruite outgrowth, microtubule bundling, and microtubule cytoskeleton assembly. Neurotoxicity occurs when neurotoxins affect the normal activity of the nervous system and ultimately, damages the nervous tissue. In the other words, any biological process that develops the nervous system is inhibited [81]. Generally, the function of the nervous system depends on neurite (axons and dendrites) outgrowth. In the case of neurotoxicity, the number and length of neurites and axonogenes are decreased [14]. *MAPT* provides microtubule-associated protein tau that is found in the nervous system and is involved in the process of stabilizing microtubules, and structures the cytoskeleton [13]. Figure 5 suggests that dexamethasone inhibits the *MAPT* gene and decreases the growth of neurons in the nervous system, and leads to neurotoxicity. Bone resorption is observed as another side effect of dexamethasone (Fig. 7). We found that the *RUNX2* (Runt-related transcription factor 2) transcription factor contributes in the osteoblast differentiation, bone resorption, and osteoclast differentiation. *RUNX2* gene is the member of RUNX transcription factors, which is closely associated with the skeletal morphogenesis [10]. Figure 7 shows that *RUNX2* inhibits the osteoclast differentiation by activating the tumor necrosis factor receptor superfamily (TNFRSF), while osteoclasts are considered as specialized cells that play a significant role in the bone matrix development [9]. Moreover, the RUNX2 transcription factor induces the tumor necrosis factor superfamily (TNFSF) ligand, TNFRSF, and bone resorption. It is found that TNF/TNFR molecules contribute in a variety of biological functions including the cell death, inflammation, brain function, and chemo-attractants for natural killer, monocytes, and neutrophils [84, 106]. TNF/TNFR proteins are classified as cytokines and it is also demonstrated that cytokines might directly contribute in the bone resorptions by increasing the proliferation and activity of cells in the osteoclast lineage [8]. TNFRSF11A gene encodes the osteoprotegerin (OPG) protein, which is also known as the osteoclastogenesis inhibitory factor (OCIF). OPG and receptor activator of nuclear factor-kappaB ligand (RANKL) are classified into two main regulators of bone resorptions. In fact, many factors, including hormones and cytokines, can change the ratio of RANKL to OPG and impact on the bone resorption rate [55].

### Raynaud disease

**Fig. 8:**
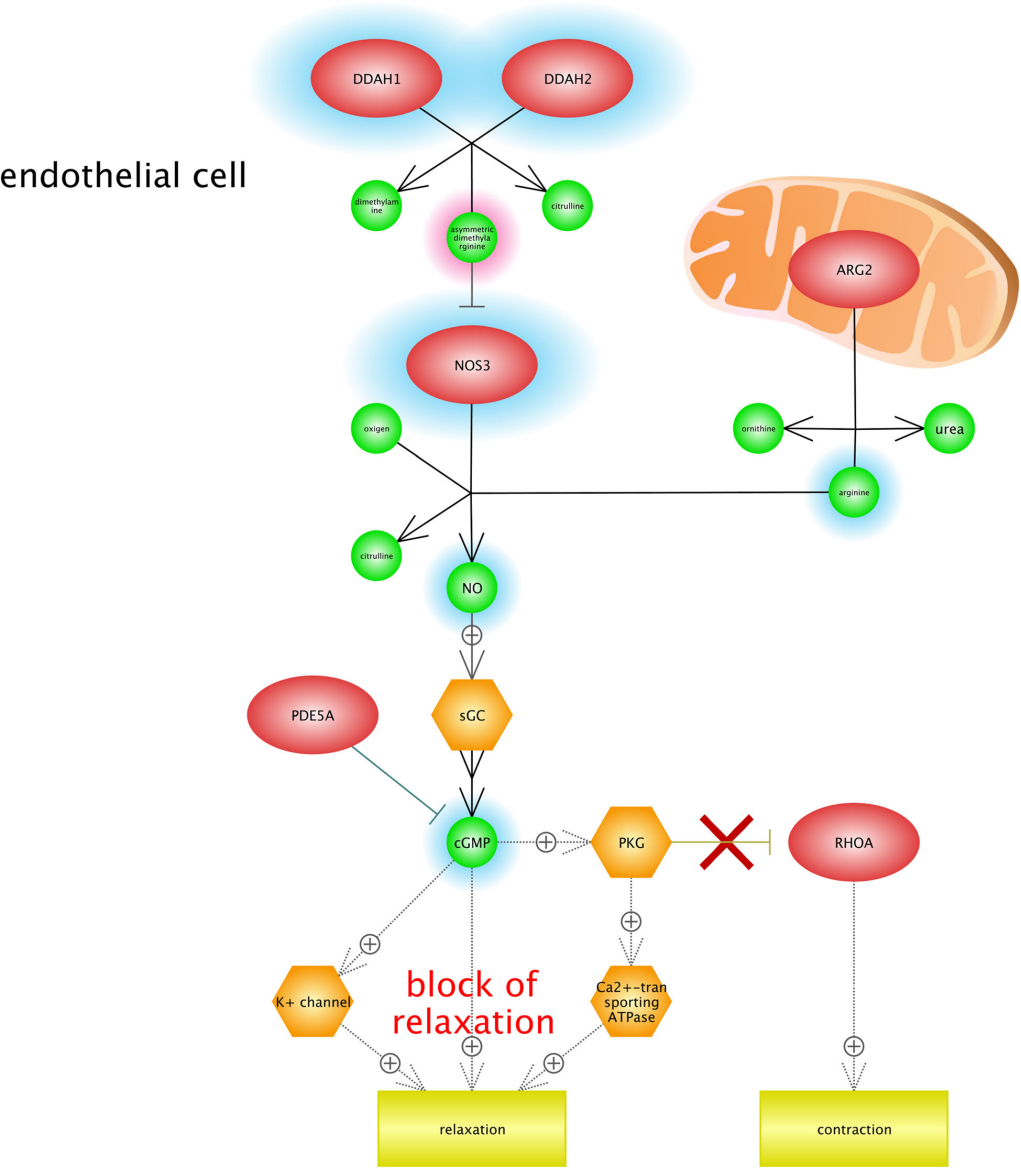
Gene network analysis in order to identify the association between hypoxia condition and Raynaud disease (RD). According to our results, NO (nitric oxide) and ADMA (Asymmetric dimethylarginine) are considered as signaling pathways thatcontribute in RD. DDAH1 and DDAH2 are two isoforms of DDA that regulate the levels of ADMA in blood. Moreover, ADMA has a negative impact on endothelial-NO synthase 3 (NOS3) and is based on the dysfunction of endothelial cells associated with RD. It was observed that the *ARG2* gene (Arginase-2), known as a mitochondria enzyme, contributes in the process of *NO* production in endothelia cells. The inhibition of NOS3 will lead to the low level of NO production; it can also lead to the increase of oxygen free radical formation and endothelial dysfunction. This figure highlights the importance of relaxation pathways’ block. It has been demonstrated that stress and emotional upsets impact on the severity of the RD

We found that hypoxia condition can lead to Raynaud disease (RD), or Raynaud’s phenomenon. It is classified as a type of disorder in which the blood flow to different parts of the body, specially the fingers, are restricted by narrowing (contracting) the arteries and leads to the discoloration of fingers. It is proved that RD is related to the vasospasm (arterial spasm), and can decrease the ratio of blood flow to various organs [90]. Figure 8 shows the significance of blocking relaxation pathways. There are two factors that impact on the severity of the RD including stress and emotional upsets; therefore, it could be very beneficial to use relaxation techniques and manage stress as the alternative treatments. Our results indicate that NO (nitric oxide) and ADMA (Asymmetric dimethylarginine) are signaling pathways that contribute in Raynaud disease. ADMA is known as an endogenous analog of l-arginine, which is metabolized to L-citrulline and dimethylamine by NG, NG-dimethylarginine dimethylaminohydrolase (DDAH). DDAH1 and DDAH2 are two isoforms of DDAH that regulate levels of blood’s ADMA [58, 83]. Moreover, *ADMA* has a negative impact on the endothelial-NO synthase 3 (NOS3) based on the dysfunction of endothelial cells associated with a variety of diseases including coronary artery disease, diabetes mellitus, and hypertension. In fact, Endothelium refers to the interior surface of blood vessels and has a significant role in the maintenance of body’s vascular structure [83, 87]. It is also observed that the *ARG2* gene (Arginase-2), which is recognized as a mitochondria enzyme, might regulate the urea cycle arginine metabolism and biosynthesis of arginine, and contributes in the process of NO production in endothelia cells. Low levels of NO production are made because of the inhibition of NOS3. It can also lead to the increase of oxygen free radical formation and endothelial dysfunction [21, 105]. Contraction was another outcome of Fig. 7. It should be noted the cold temperature is one of the important factors that can play a role in Raynaud attack. In high altitude conditions by decreasing the temperature, the body restricts blood flow to the skin to save heat body and to sustain the core body temperature. However, in this situation, the probability of constricting blood vessels will increase and it may lead to further limiting blood flow and finally Raynaud attack [68].

### Carcinogenesis

We found that hypoxia conditions might contribute in the tumorigenesis such as breast cancer, ovarian cancer, and colorectal cancer. The UV radiation in highlands leads to the *DNA* damage in high-altitude conditions; it is also proved that there is a close association between DNA damages and cancers. Moreover, hypoxia-inducible factors (HIFs) are attributed to multiple steps of tumorigenesis including tumor formation, progression, and response to therapy [79].

**Fig. 9:**
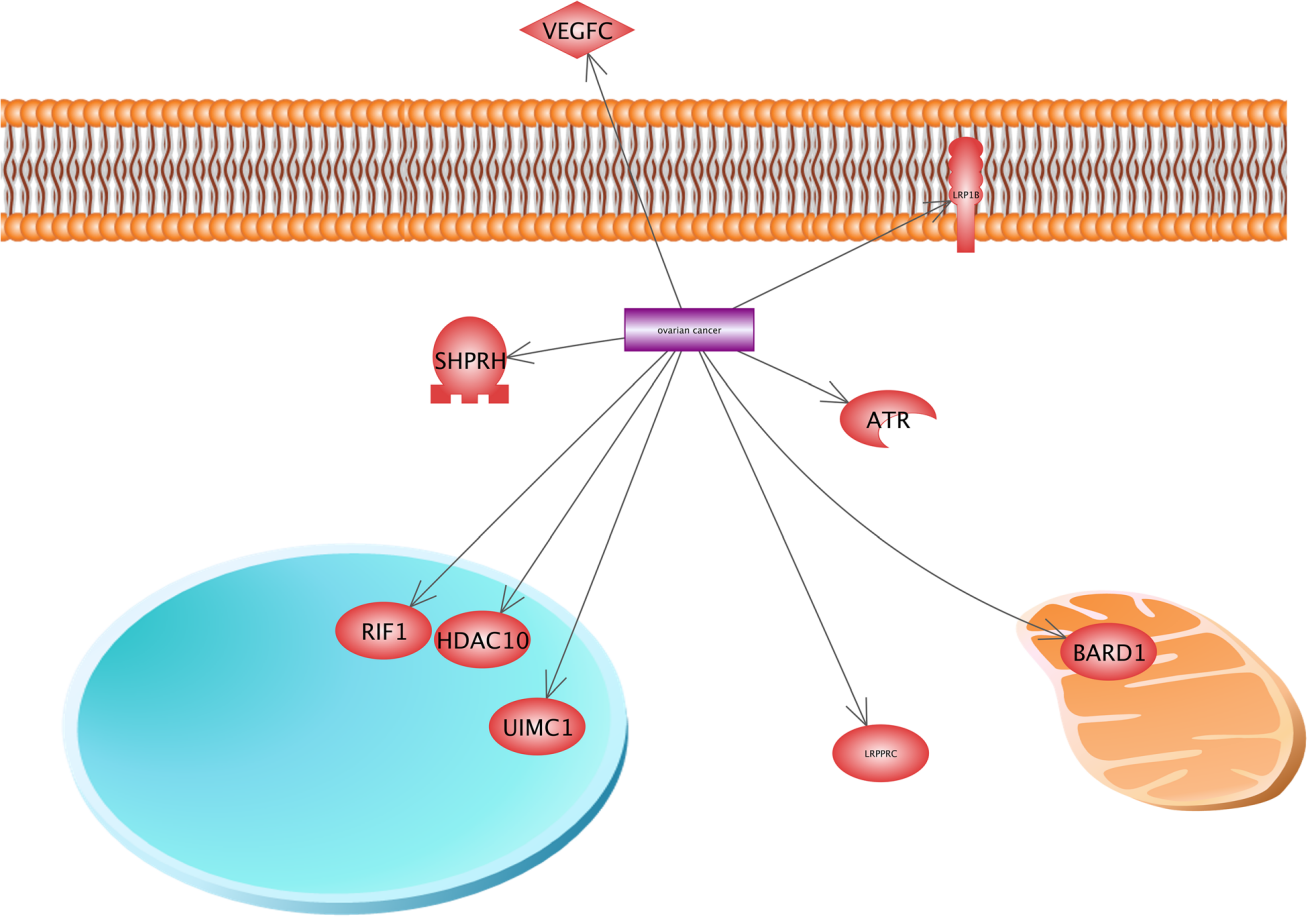
Gene network construction of hypoxia-associated genes for the description of the ovarian cancer (OC). Results show that VEGF (vascular endothelial growth factor), considered as the protein ligand, is involved in ovarian carcinoma. VEGF enhances cancer cell mobility and promotes the cancer cell metastasis. *LRP1B* gene encodes low-density lipoprotein receptor-related protein 1B, which plays several important roles in the normal cell function and development due to their interactions with multiple ligands. The disruption of LRP1B is based on several types of cancers and could be considered as the tumor suppressor. Deletion or downregulation of *LRP1B* is associated with the chemotherapy resistance in OC

Results indicated that the VEGF (Vascular endothelial growth factor), known as a protein ligand, is involved in the ovarian carcinoma (OC) (Fig. 9). The VEGF gene family encodes five polypeptide growth factors including VEGF-A, VEGF-B, VEGF-C, VEGF-D, and VEGF-E, characterized by angiogenic and lymphangiogenic properties in neoplasms [94]. VEGF enhances the cancer cell mobility, and also promotes the cancer cell metastasis [93]. It was observed that LRP1B, which is identified as a receptor, contributes in OC. *LRP1B* gene encodes low-density lipoprotein receptor-related protein 1B, which plays several significant roles in the normal cell function and development due to their interactions with multiple ligands. The disruption of LRP1B is based on several types of cancers and can be considered as the tumor suppressor [62]. Deletion or downregulation of LRP1B is associated with chemotherapy resistance in OC [20].

**Fig. 10:**
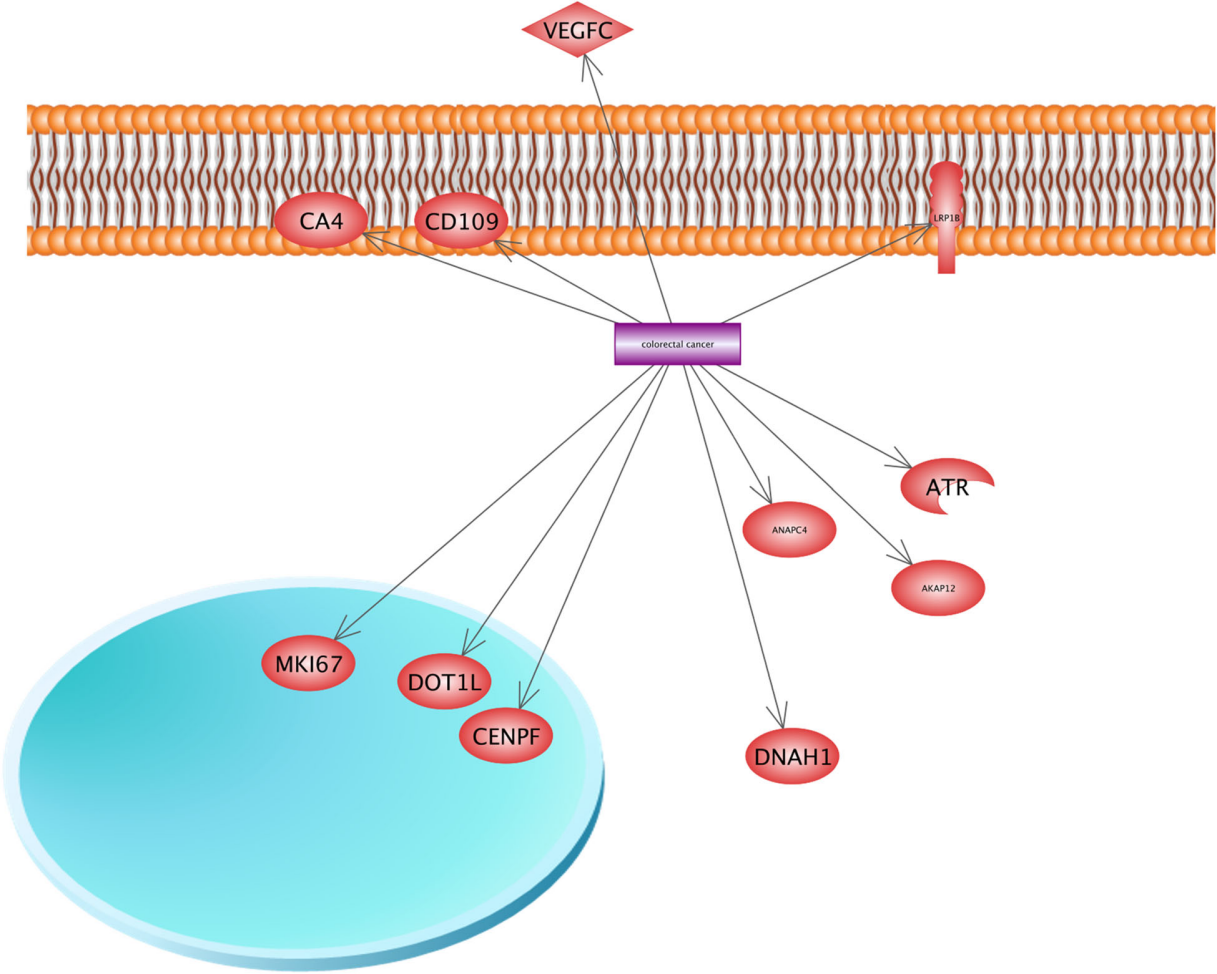
The role of the hypoxia-regulated gene in colorectal cancer. We found that *ATR* serine/threonine kinase is known as the *DNA* damage sensor, and is also activated upon genotoxic stresses such as ionizing radiation, ultraviolet light, and DNA replication stalling. For instance, it has a regulatory role in the response to *DNA* double-strand breaks. It is reported that the mutation in *ATR* serine/threonine kinase leads to a wide variety of types of tumors

Colorectal cancer (CRC) is known as the most common cancer in the world and makes 9% of all types of cancers [77]. We found that 11 candidate genes including ligands, receptors, and protein kinases are involved in CRC. Figure 10 illustrates that ATR serine/threonine kinase is related to CRC. ATR serine/threonine kinase is known as the *DNA* damage sensor which is activated in the conditions of genotoxic stresses such as ionizing radiation, ultraviolet light, and *DNA* replication stalling. For instance, it has a regulatory role in response to *DNA* double-strand breaks [6]. It is reported that mutation in ATR serine/threonine kinase leads to wide types of tumors [113].

**Fig. 11:**
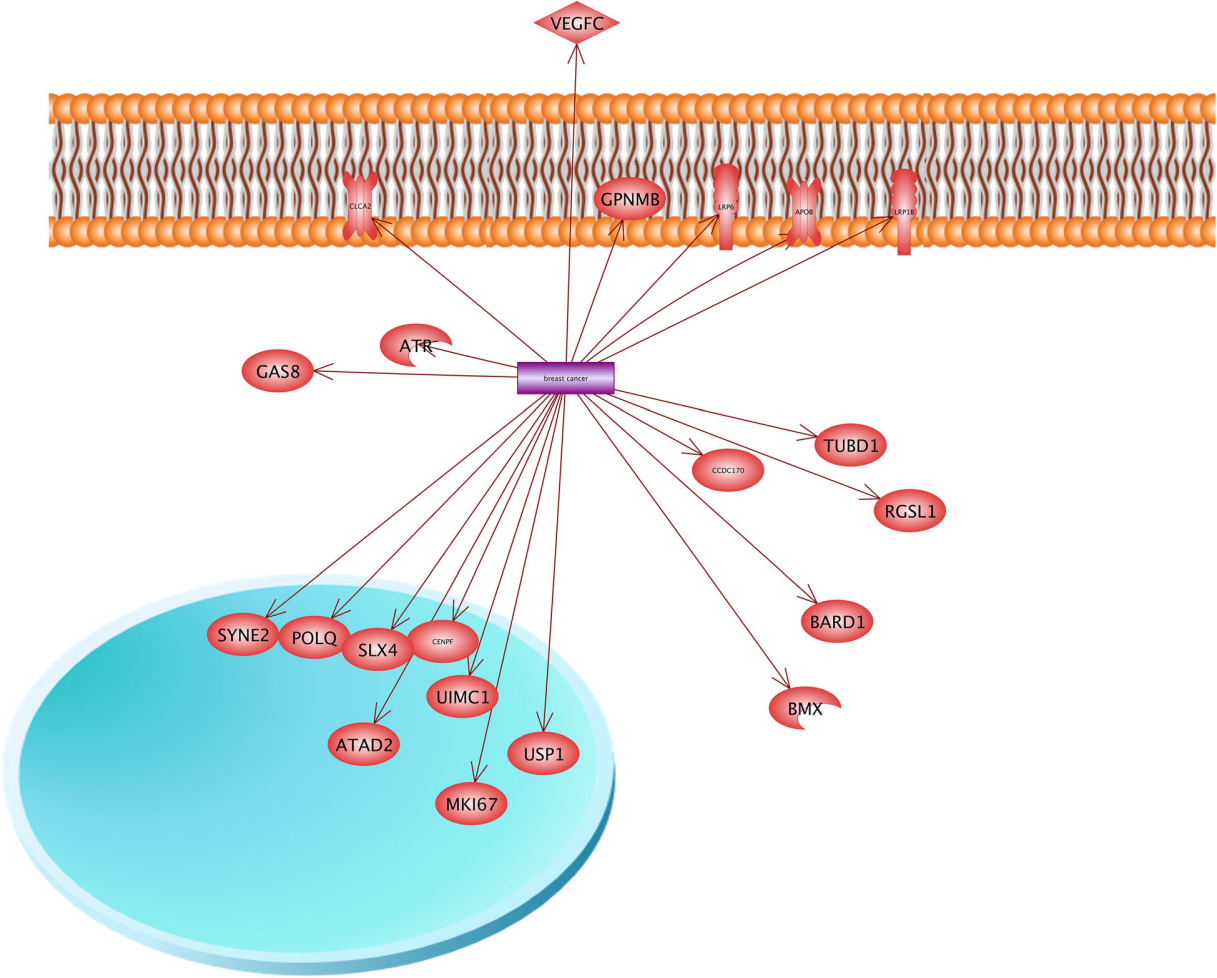
Gene network analysis for the identification of roles of key candidate genes in breast cancer. Our gene network analysis indicated that two protein transporters including APOB (Apolipoprotein B) and *CLCA2* genes are involved in breast cancer. APOB is one important member of body’s lipid profile and is considered as an essential factor for cholesterol hemostasis. The abnormal level of APOB is associated with different diseases including cardiovascular, inflammation, and tumorigenesis. Furthermore, it was reported that rs693 and rs1042031 polymorphisms that existed in the *APOB* gene increased the risk of breast cancer. Another result showed that CLCA2 is involved in the calcium-activated chloride channel regulator family and regulates transferring the chloride across the plasma membrane. The over-expression of the *CLCA2* gene in negative cell lines of CLCA2 will lead to the reduction of metastasis and tumorigenesis

Our gene network analysis indicated that two protein transporters including APOB and CLCA2 contribute in the breast cancer (Fig. 11). APOB (Apolipoprotein B) is recognized as an important member of body’s lipid profile, which is an essential factor of cholesterol hemostasis [64]. The abnormal level of APOB is associated with different diseases such as cardiovascular, inflammation, and tumorigenesis [7, 30]. Furthermore, Liu et al. [63] reported that rs693 and rs1042031 polymorphisms that existed in the APOB gene increased the risk of breast cancer.

We also found that the *CLCA2* gene can be considered in breast cancer. Investigations have shown the association of *CLCA2* with the development of breast cancer and metastasis [59, 76]. This protein is contained in the calcium-activated chloride channel regulator family that regulates transferring chloride across the plasma membrane. Moreover, the *CLCA2* gene is known as the tumor suppressor. When the *CLCA2* gene was over-expressed in negative cell lines for CLCA2, the tumorigenicity and metastasis capability of cell lines were significantly reduced [59]. It should be noted that CLCA2 plays a critical role in the epithelial differentiation of breasts and the process of promoting methylation will also lead to the downregulation of CLCA2 in breast cancers [78].

**Fig. 12:**
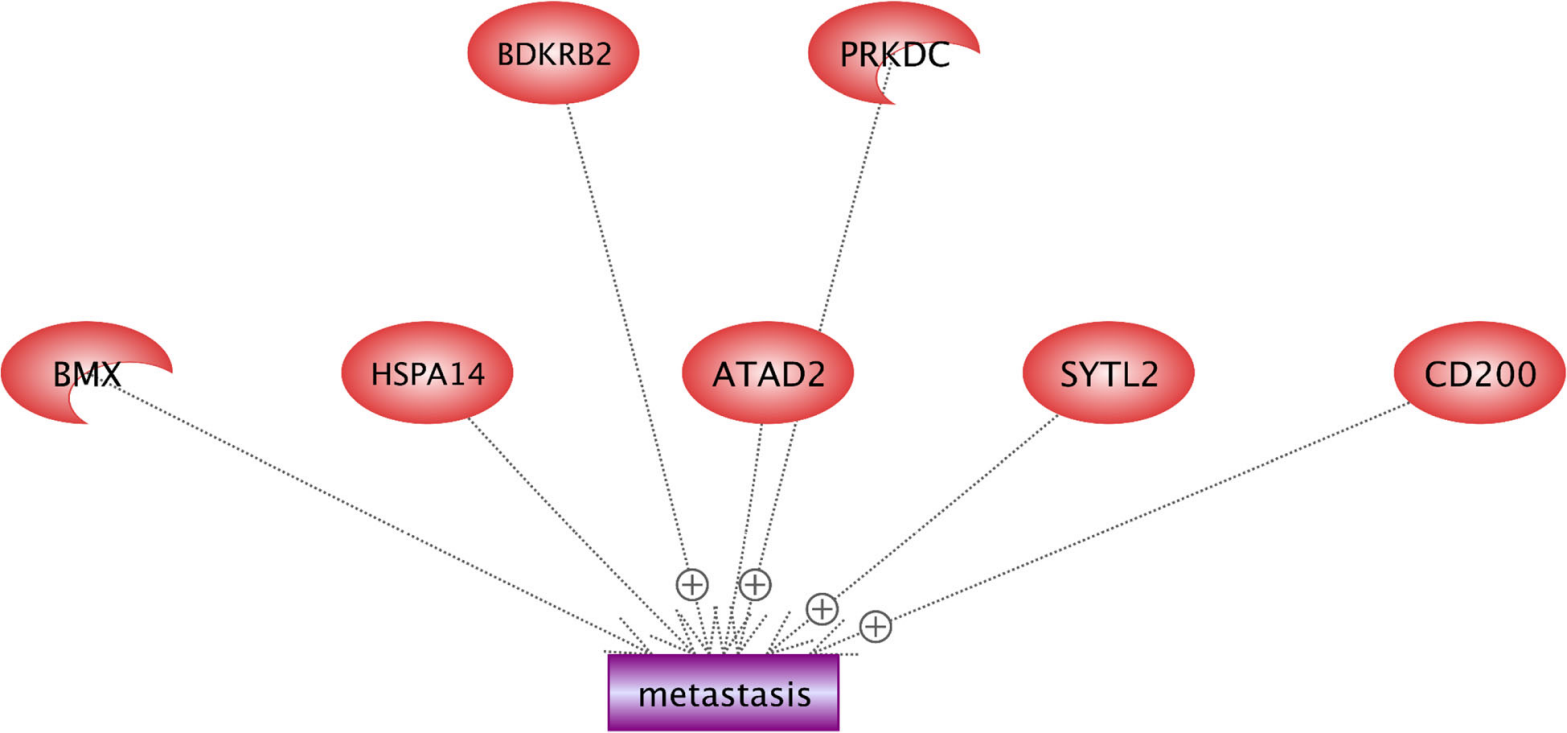
Identification of regulatory roles of candidate genes in metastasis under hypoxia condition. We found that *PRKDC*, *CD200*, *SYTL2*, and *BDKRB2* enhanced the metastasis event. *PRKDC* encodes DNA-PKcs protein that participates in the development of the immune system and is usually overexpressed in cancer metastasis. SYTL2 (synaptotagmin like 2) is one of the members of C2 domain-containing protein family. It is demonstrated that SYTL2 contributes in ovarian cancer and its overexpressed one can promote the metastatic potential in ovarian cancer. Immunoglobulin superfamily (IgSF) contains two domains including *CD200* and *CD200*-R, and their interaction is also involved in functions of myeloid cells. The overexpression of *CD200* in breast cancer can be considered as an important risk factor for metastasis. Bradykinin contains nine amino acid peptide chains and is implicated in many responses including vasodilation, edema, smooth muscle spasm, and pain fiber stimulation

Metastasis is recognized as the main complex problem of cancer treatments and is defined as the extension of cancer cells from an initial tumor to different parts of the body [31, 43]. Results indicate that *PRKDC*, *CD200*, *SYTL2*, and *BDKRB2* enhanced the metastasis event (Fig. 12). *PRKDC* encodes DNA-PKcs protein that participates in the development of the immune system and is usually overexpressed in the cancer metastasis. Kotula et al. [56] demonstrated that there are 103 secretion proteins controlled by DNA-PKcs, and most of them are associated with metastasis. Therefore, the *PRKDC* gene contributes in the invasion of cancer cells by regulating secretion proteins [56]. *SYTL2* (synaptotagmin like 2) is contained in the C2 domain-containing protein family.

It has been demonstrated that *SYTL2* contributes in the ovarian cancer, and can also promote the metastatic potential in ovarian cancer when it is overexpressed [95]. Immunoglobulin superfamily (IgSF) contains two domains of extracellular and intracellular, including CD200 and CD200-R; their interaction is also involved in functions of myeloid cells [98]. The overexpression of CD200 in breast cancer can be considered as an important risk factor of metastasis [35]. The receptor of Bradykinin is encoded by the *BDKRB2* gene. Bradykinin contains nine amino acid peptide chains, and is implicated in many responses including vasodilation, edema, smooth muscle spasm, and pain fiber stimulation [111].) showed that the expression of *BDKRB2* is negatively associated with miR-129-1-3p in the gastric cancer, while miR-129-1-3p inhibits metastasis by targeting *BDKRB2*.

### Cardiomyopathy

**Fig. 13:**
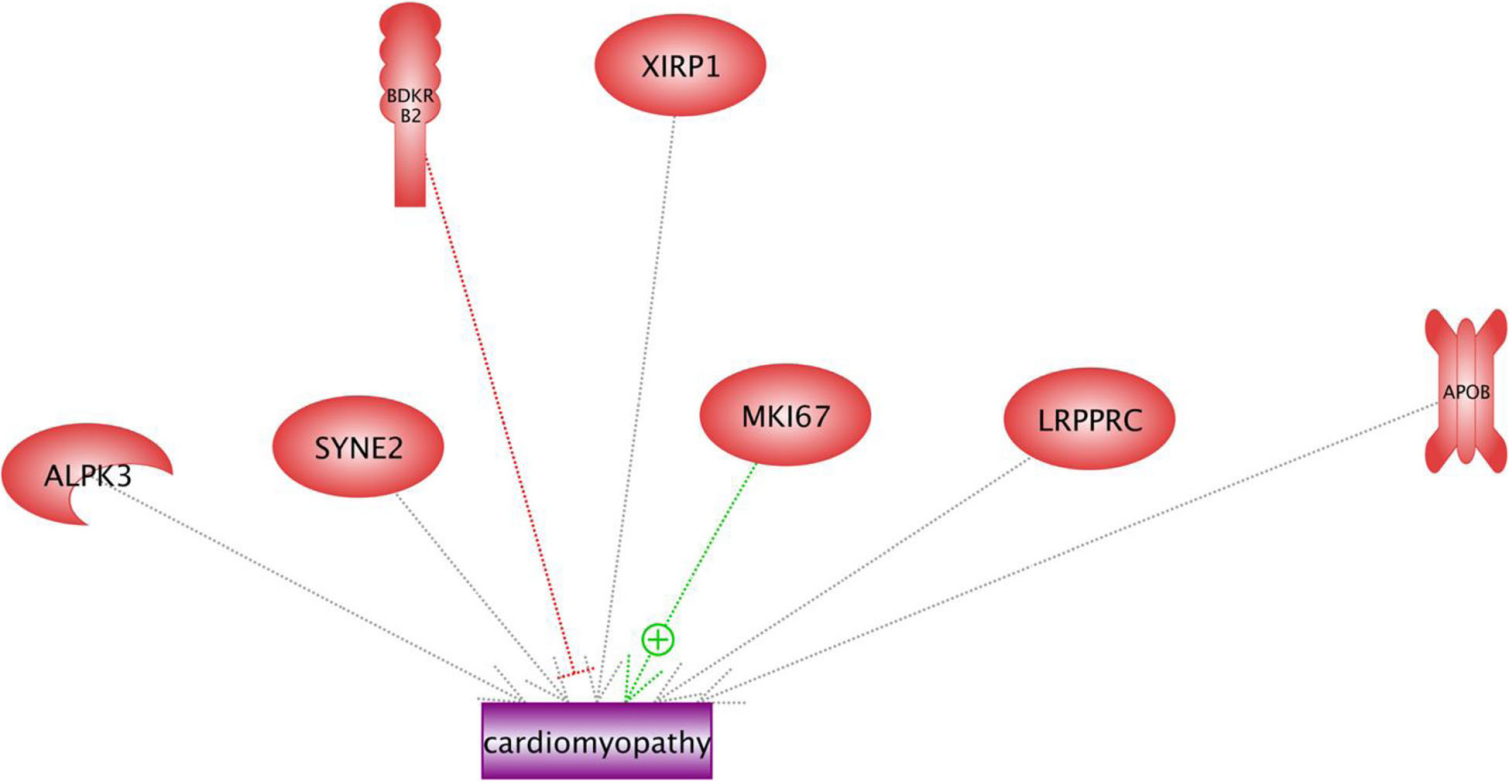
The candidate genes involved in cardiomyopathy and their regulation roles in the low oxygen concentration. Cardiomyopathy is related to the abnormal formation of muscle heart and leads to the hypertrophic muscle. Heart muscle cells are sensitive to the low oxygen concentration that results in the death of cardiac myocytes. Results of this study showed that MKI67 induces cardiomyopathy, and BDKRB2 inhibits the disorder. *MKI67* gene encodes the nuclear protein, which is related to cellular proliferation and might also play a role in the chromatin organization; however, it is poorly understood. The regulatory roles of other enriched genes in cardiomyopathy networks (including *SYNE2*, *APOB*, *XIRP1*, *ALPK3*, and *LRPPRC)* are not clear

It is reported that cardiomyopathy is related to the abnormal formation of muscle heart that leads to the hypertrophic muscle. Heart muscle cells are sensitive to low oxygen concentration, which itself results in the death of cardiac myocytes [47]. Results of the current study showed that MKI67 induces cardiomyopathy, and BDKRB2 inhibits the disorder (Fig. 13). *MKI67* gene encodes the nuclear protein, which is related to cellular proliferation and might also play a critical role in the chromatin organization; however, it is poorly understood (Uniprot: KI67_HUMAN). As we described earlier, BDKRB2, known as the receptor of Bradykinin, contributes in edema, smooth muscle spasm, and pain fiber stimulation. Moreover, Gohlke et al. [33] demonstrated that Bradykinin involved in cardiac protection by blood pressure reduction, changes in renal blood flow and tubular function, inflammatory reactions. The regulatory roles of other enriched genes in cardiomyopathy networks (including *SYNE2*, *APOB*, *XIRP1*, *ALPK3*, and *LRPPRC*) are not clear (Fig. 13).

## Discussion

Several attempts have been made to describe the association between high-altitude conditions and the incidence of diseases. However, adaptation mechanisms to high-altitude conditions are complex and include several biological pathways such as gene networks, epigenetic regulation, and different gene expression patterns. Moreover, there are irregular patterns for disorders to occur [17, 46, 49]. Therefore, we tried to identify candidate genes for diseases and explore biological pathways between hypoxia-associated genes and human diseases by gene network analysis.

High-altitude conditions can be investigated like harsh environments because there are factors including two-point perspective, low oxygen concentration, and UV radiation. The low level of oxygen is considered as the most important problem of high-altitude conditions. Results of this study showed that several hypoxia-associated genes are enriched in brain injury disorders including PD and AD. It is recognized as a possible explanation that the normal activity of the nervous system is extremely dependent upon normal levels of oxygen. Any disruption of oxygen consumption by brain neurons can lead to mitochondrion dysfunction and neuron necrosis. This result is in accordance with findings of Winklhofer and Haass [114] who showed that the mitochondrial dysfunction plays a central role in PD, and several PD-associated genes impact on biological pathways are associated with mitochondrial integrity. Furthermore, a strong relationship between the function of the central nervous system and supply of oxygen has been reported by Mukandala et al. [67], who indicated that the effect of low levels of oxygen is based on the release of inflammation’s agents (including cytokines of neuron cells) in the early response to hypoxia that will lead to cell death. However, it was hypothesized that the inflammation response plays a protective role against brain damages. Inflammatory reactions could be having contrasting dual roles. For instance, released cytokines could improve the synaptic plasticity, modulate neuronal excitability, and also and stimulated neurogenesis and neurite outgrowth. However, a strong inflammation response in neuron cells induces overexpression or dysregulation of cytokine, which accelerates neurodegeneration [89]. It has been demonstrated, neurodegenerative disorders such as PD and AD are related to the irreversible loss of structure and dysfunction of neurons [19, 54]. Furthermore, the comparable results indicated that there is an association between the hypoxic condition and PD-related protein including α-synuclein (α-Syn), and is also considered as a key protein to understand PD based on the following reasons. First α-Syn protofibrils contributes in lewy bodies’ formation and it is believed that the clump of α-Synuclein plays a critical role in PD [118]. Second, the over-expression of α-Syn is based on the death of neuron cells and third, the mutation in α-Syn gene encoding is responsible for the autosomal dominant PD [54].

Another problem in high-altitude conditions is UV radiation. It is shown that high-dose UV radiation can lead to DNA damage, cell apoptosis, and tissue injury of mammals [96, 117]. Similarly, our results support previous researches by enriching the gene associated- hypoxia in cancer networks. *DNA* damages are responsible for the variety of human diseases. For example, there is a close association between tumorigenesis and *DNA* damages and consequently, numerous investigations aimed to explore the association between high-altitude and cancer mortality [12, 101, 102]. This study showed that metastasis, breast, ovarian and colorectal cancers occur in high-altitude conditions frequently. Findings of the current study are consistent with those of Hongo et al. [42] who recognized that hypoxia enhances the colon cancer through the activation of epithelial-mesenchymal transition in colon cancer cells. It is demonstrated by McEvoy et al. [65] that there is an association between hypoxia and features of cancers such as metastasis, abnormal cellular proliferation, and prevention of apoptosis. Additionally, they describe the effect of hypoxia on chemo-resistance in an ovarian cancer model.

This study indicates that many pathways of drug metabolisms are markedly altered under hypoxia conditions. Reviewing the literature, many investigations tried to describe the effect of hypoxia on pathways of drug metabolisms [45, 57, 74, 107]. There are several explanations for these results. First, for instance, many pathways of drug metabolisms such as oxidation, sulfation, and acetylation depend on the oxygen availability; therefore, the study of drug metabolism pathways in high-altitude conditions can explain the side effect of drugs [57]. Second, hypoxia can impact on the pharmacokinetic features of some drugs [34]. Third, the delivery of cytotoxic drugs can be insufficient because of the negative effects of hypoxia on blood vessels and fluctuating blood flow. Thus, the concentration of cytotoxic drugs in normal tissues is higher than in cancerous tissues and will lead to the drug resistance and the destruction of normal cells. Consequently, the tumor will be resistant to chemotherapeutic treatments [115].

We found that hypoxia-associated genes can be considered in heart failure diseases. Several studies obtained evidences about the role of hypoxia in cardiomyopathy [24, 104, 112]. In addition, this study showed that MKI67 had a positive impact on cardiomyopathy. It is reported by comparable results that cardiomyocytes are able to karyokinesis (division of a cell nucleus during mitosis) in the absence of cytokinesis and are based on the increased polyploidy. Also, MKI67 proteins are expressed in the S, G1 S, and G2 phases of mitosis [80]. Furthermore, HIF expression in low oxygen conditions induced the cellular proliferation proteins such as *MKI67* [5]. Considering evidences together, the polyploidy of cardiomyocytes and induced nuclear proteins of cell proliferation have significant roles in the myocardial hypertrophy disease [86]. In contrast, our results showed that *BDKRB2* genes inhibit cardiomyopathy. Similar results were obtained by Alves et al. [3] who showed that *BDKRB2* polymorphisms are associated with cardiovascular phenotypes and suitable performance. This could be explained by the fact that the abnormal structure of *BDKRB2* gene is related to heart failures [60]. In addition, the existence of mutation in *BDKRB2* gene leads to an increase in the number of receptors of Bradykinin on the cell surface membrane and vasodilation [3].

## Conclusion

The gene network analysis was carried out based on hypoxia-associated genes using an animal model in this study. We found that hypoxia-associated genes were enriched in several gene networks of disorders including Parkinson, Alzheimer, cardiomyopathy, drug toxicity, and different types of cancers. There are two main reasons that can be described for human diseases in high-altitude conditions. First, UV radiation is known as one of the most important problems in high-altitude conditions and is also responsible for a variety of human diseases. Second, low oxygen concentration influences many biological pathways, especially mitochondrion organelle. The existence of alterations in mitochondrion functions could be considered as the key symptom of human’s diseases. Using the gene network analysis, this study visually presents the mechanism of disorders; it also provides new insights about key candidate genes of diseases.

## Supplementary Information

Additional file 1. Supplementary material

Additional file 2: Table S1. The list of selected hypoxia- associated genes for gene network analysis. Table S2: Principle component analysis of phenotypic traits for discriminate analysis and clustering the highland and lowland populations. Table S3: The classification of highland and lowland chickens based on the discriminate analysis.

## Data Availability

The genome resequencing reads were deposited in the browser of chickenSD database (http://bigd.big.ac.cn/chickensd/)

## Abbreviations

AD: Alzheimer’s disease
ADMA: Asymmetric dimethylarginine
APP: Amyloid precursor protein
APOB: Apolipoprotein B
ARG2: Arginase-2
Aβ: β-amyloid peptide
BDKRB2: Bradykinin receptor B2
CD200: Cluster of Differentiation 200
CLCA2: Chloride channel Accessory 2
CYCS: Cytochrome c
CRC: Colorectal cancer
DDAH: Dimethylarginine dimethylaminohydrolase
DNM1L: Dynamin 1 Like
GLUTs: Glucose transporters family
HIF1A: Hypoxia-inducible factor 1-alpha
IgSF: Immunoglobulin superfamily
LRP: LDL Receptor Related Protein
MCU: Mitochondrial calcium uniporter
MKI67: Marker of Proliferation Ki-67
MPTP: Mitochondrial permeability transition pore
NADH: Nicotinamide-adenine dinucleotide
NOS: Nitric oxide synthase 1
NR3C1: Nuclear receptor subfamily 3 group C member 1
OC: Ovarian carcinoma
OCIF: Osteoclastogenesis inhibitory factor
OPA1: Optic atrophy 1
OPG: Osteoprotegerin
PD: Parkinson disease
PRKDC: Protein Kinase, DNAActivated
RANKL: Receptor activator of nuclear factor-kappaB ligand
RD: Raynaud disease
ROS: Reactive oxygen species
RUNX2: Runt-related transcription factor 2
SGK1: Serine/threonine-protein kinase
SLC2A4: Solute carrier family 2 member 4
SOD2: Superoxide Dismutase-2
α-Syn or SNCA: α-synuclein
SYTL2: Synaptotagmin like 2
TOM: Translocase of the outer mitochondrial membrane
TNFSF: Tumor necrosis factor superfamily
TNFRSF: Tumor necrosis factor receptor superfamily
UV: Ultraviolet
VEGF: Vascular endothelial growth factor
WNT-FZD: Wingless/Int1-Frizzled

## References

[1] Alavi MV, Fuhrmann N (2013) Dominant optic atrophy, OPA1, and mitochondrial quality control: understanding mitochondrial network dynamics. Mol Neurodegener 8, 32. 10.1186/1750-1326-8-32. 10.1186/1750-1326-8-32 PMC3856479 24067127

[2] Arenas E, Saltó C, Villaescusa C (2015). WNT signaling in midbrain dopaminergic neuron development and cell replacement therapies for Parkinson’s disease. SpringerPlus.

[3] Alves CR (2013). Vascular reactivity and ACE activity response to exercise training are modulated by the+9/− 9 bradykinin B2 receptor gene functional polymorphism. Physiol Genomics.

[4] Beitz JM (2014). Parkinson’s disease: a review. Front Biosci.

[5] Bekeredjian R (2010). Conditional HIF-1α expression produces a reversible cardiomyopathy. PloS One.

[6] Blackford AN, Jackson SP (2017). ATM, ATR, and DNA-PK: the trinity at the heart of the DNA damage response. Mol Cell.

[7] Borgquist S (2016). Apolipoproteins, lipids and risk of cancer. Int J Cancer.

[8] Boyce B (2013). Advances in the regulation of osteoclasts and osteoclast functions. J Dent Res.

[9] Boyle WJ, Simonet WS, Lacey DL (2003). Osteoclast differentiation and activation. Nature.

[10] Bruderer M, Richards R, Alini M, Stoddart MJ (2014). Role and regulation of RUNX2 in osteogenesis. Eur Cell Mater.

[11] Burt DW (2007). Emergence of the chicken as a model organism: implications for agriculture and biology. Poultry Sci.

[12] Burtscher M (2014). Effects of living at higher altitudes on mortality: a narrative review. Aging Dis.

[13] Caillet-Boudin ML, Buée L, Sergeant N, Lefebvre B (2015). Regulation of human MAPT gene expression. Mol Neurodegener.

[14] Ceresa C (2014). Characterization of and protection from neurotoxicity induced by oxaliplatin, bortezomib and epothilone-B. Anticancer Res.

[15] Cha MY, Kim DK, Mook-Jung I (2015). The role of mitochondrial DNA mutation on neurodegenerative diseases. Exp Mol Med.

[16] Chakraborty C, Hsu CH, Wen ZH, Lin CS, Agoramoorthy G (2009). Zebrafish: a complete animal model for in vivo drug discovery and development. Currt Drug Metab.

[17] Chavala MA (2018). A journey between high altitude hypoxia and critical patient hypoxia: What can it teach us about compression and the management of critical disease?. Med Intensiva (Engl Ed).

[18] Cogburn LA, Porter TE, Duclos MJ, Simon J, Burgess SC, Zhu JJ, Cheng HH, Dodgson JB, Burnside J (2007). Functional Genomics of the Chicken A Model Organism. Poult Sci.

[19] Cookson MR (2009) α-Synuclein and neuronal cell death. Mol Neurodegener 4, 9 10.1186/1750-1326-4-9 PMC2646729 19193223

[20] Cowin PA (2012). LRP1B deletion in high-grade serous ovarian cancers is associated with acquired chemotherapy resistance to liposomal doxorubicin. Cancer Res.

[21] de Haro Miralles J (2009). Nitric oxide: link between endothelial dysfunction and inflammation in patients with peripheral arterial disease of the lower limbs. Interact Cardiov Tho.

[22] Doan R et al (2012) Whole-genome sequencing and genetic variant analysis of a Quarter Horse mare. BMC Genomics. 13. 10.1186/1471-2164-13-78 10.1186/1471-2164-13-78 PMC3309927 22340285

[23] Dias M et al (2017) SNP detection using RNA-sequences of candidate genes associated with puberty in cattle. Genet Mol Res 16. 10.4238/gmr16019522 10.4238/gmr16019522 28340271

[24] Dimitiru L, Dimitiru A, Stamatin M (2012). 1156 Hypoxic Perinatal Cardiomyopathy-Diagnosis and Evolution. Arch Dis Child.

[25] Dodgson JB, Romanov MN (2004). Use of chicken models for the analysis of human disease. Current Protocols in. Hum Genet.

[26] Eales K, Hollinshead K, Tennant D (2016). Hypoxia and metabolic adaptation of cancer cells. Oncogenesis.

[27] Ebrahimie E, Moussavi-Nik S-H, Newman M, Lardelli M (2016). A zebrafish homologue of the Alzheimer’s disease-associated PRESENILIN isoform PS2V regulates inflammatory and other responses to hypoxic stress. J Alzheimers Dis.

[28] Esfandiari P, Dadpasand M, Kharrati-Koopaee H, Atashi H, Gharghi A, Niazi A (2020). Bioinformatics, phylogenetic and variant association analysis of Ovocalyxin-32 gene reveals its contribution to egg production traits in native chickens. Animal Gene.

[29] Esteras N (2013). Calmodulin levels in blood cells as a potential biomarker of Alzheimer’s disease. Alzheimers Res Ther.

[30] Fouchier SW (2005). et al., High frequency of APOB gene mutations causing familial hypobetalipoproteinaemia in patients of Dutch and Spanish descent. J Med Genet.

[31] Geiger TR, Peeper DS (2009). Metastasis mechanisms. Biochimica et Biophysica Acta (BBA)-Reviews on. Cancer.

[32] Giarraputo J (2016). Medical morbidities and DNA methylation of NR3C1 in preterm infants. Pediatr Res.

[33] Gohlke P, Tschöpe C, Unger T (1997) Bradykinin and cardiac protection. Hypertens Heart:159–172 10.1007/978-1-4615-5385-4_17 9433522

[34] Gong W (2017). Effect of hypoxia on the pharmacokinetics and metabolism of zaleplon as a probe of CYP3A1/2 activity. RSC Adv.

[35] Gorczynski RM, Clark DA, Erin N, Khatri I (2011). Role of CD200 expression in regulation of metastasis of EMT6 tumor cells in mice. Breast Cancer Res Tr.

[36] Grisham M, Jourd'Heuil D, Wink D (2000). chronic inflammation and reactive oxygen and nitrogen metabolism–implications in DNA damage and mutagenesis. Aliment Pharm Ther.

[37] Haining RL, Achat-Mendes C (2017). Neuromelanin, one of the most overlooked molecules in modern medicine, is not a spectator. Neural Regen Res.

[38] Halestrap AP (2009). What is the mitochondrial permeability transition pore?. J Mol Cell Cardiol.

[39] Harbauer AB, Zahedi RP, Sickmann A, Pfanner N, Meisinger C (2014). The protein imports machinery of mitochondria—a regulatory hub in metabolism, stress, and disease. Cell Metab.

[40] Hewitt VL, Whitworth AJ (2017). Mechanisms of Parkinson’s disease: Lessons from Drosophila. Curr Top Dev Biol.

[41] Hinkelbein J (2017). Thirty Minutes of Hypobaric Hypoxia Provokes Alterations of Immune Response, Haemostasis, and Metabolism Proteins in Human Serum. Int J Mol Sci.

[42] Hongo K (2013). Hypoxia enhances colon cancer migration and invasion through promotion of epithelial-mesenchymal transition. J Surg Res.

[43] Hunter KW, Crawford NP, Alsarraj J (2008). Mechanisms of metastasis. Breast Cancer Res.

[44] JBonda D (2011). The mitochondrial dynamics of Alzheimer's disease and Parkinson's disease offer important opportunities for therapeutic intervention. Curr Pharm design.

[45] Jones DP, Aw TY, Shan X (1989). Drug metabolism and toxicity during hypoxia. Drug Metab Rev.

[46] Julian CG (2017). Epigenomics and human adaptation to high altitude. Journal of Applied Physiology.

[47] Julian R (2007). The response of the heart and pulmonary arteries to hypoxia, pressure, and volume. A short review. Poultry Sci.

[48] Kann O, Schuchmann S, Buchheim K, Heinemann U (2003). Coupling of neuronal activity and mitochondrial metabolism as revealed by NAD (P) H fluorescence signals in organotypic hippocampal slice cultures of the rat. Neurosci.

[49] Ke J, Wang L, Xiao D (2017) Cardiovascular adaptation to high-altitude hypoxia. Hypoxia and human diseases 117

[50] Kerr JB (2005). Understanding the factors that affect surface ultraviolet radiation. Opt Eng.

[51] Kharrati-Koopaee H, Ebrahimie E, Dadpasand M, Niazi A, Esmailizadeh A (2019) Genomic analysis reveals variant association with high altitude adaptation in native chickens. Sci Rep. 10.1038/s41598-019-45661-7 10.1038/s41598-019-45661-7 PMC6592930 31239472

[52] Khurana P, Sugadev R, Jain J, Singh SB (2013) HypoxiaDB: a database of hypoxia-regulated proteins. Database. 10.1093/database/bat074 10.1093/database/bat074 PMC3813937 24178989

[53] Khurana P, Tiwari D, Sugadev R, Sarkar S, Singh SB (2016). A comprehensive assessment of networks and pathways of hypoxia-associated proteins and identification of responsive protein modules. Netw Model Anal Health Inform Bioinform.

[54] Kim T, Vemuganti R (2017). Mechanisms of Parkinson’s disease-related proteins in mediating secondary brain damage after cerebral ischemia. J Cereb Blood Flow Metab.

[55] Kostenuik PJ (2005). Osteoprotegerin and RANKL regulate bone resorption, density, geometry and strength. Curr Opin Pharmacol.

[56] Kotula E (2015). DNA-PKcs plays role in cancer metastasis through regulation of secreted proteins involved in migration and invasion. Cell Cycle.

[57] Lee K, Roth RA, LaPres JJ (2007). Hypoxia, drug therapy and toxicity. Pharmacol Therapeut.

[58] Lee W (2018). Asymmetric dimethylarginine (ADMA) is identified as a potential biomarker of insulin resistance in skeletal muscle. Sci Rep.

[59] Li X, Cowell JK, Sossey-Alaoui K (2004). CLCA2 tumour suppressor gene in 1p31 is epigenetically regulated in breast cancer. Oncogene.

[60] Li Y (2012). Bradykinin β2 receptor− 58 T/C gene polymorphism and essential hypertension: a meta-analysis. PloS One.

[61] Ling N (2003) Rotenoneôa review of its toxicity and use for fisheries management

[62] Liu CX, Li Y, Obermoeller-McCormick LM, Schwartz AL, Bu G (2001). The putative tumor suppressor LRP1B, a novel member of the low density lipoprotein (LDL) receptor family, exhibits both overlapping and distinct properties with the LDL receptor-related protein. J Biol Chem.

[63] Liu X (2013). Associations of polymorphisms of rs693 and rs1042031 in apolipoprotein B gene with risk of breast cancer in Chinese. Jpn J Clin Oncol.

[64] Ma MZ, Yuan SQ, Chen YM, Zhou ZW (2018). Preoperative apolipoprotein B/apolipoprotein a1 ratio: a novel prognostic factor for gastric cancer. OncoTarget Ther.

[65] McEvoy LM (2015). Identifying novel hypoxia-associated markers of chemoresistance in ovarian cancer. BMC Cancer.

[66] Michiels C (2004). Physiological and pathological responses to hypoxia. Ame J Pathol.

[67] Mukandala G, Tynan R, Lanigan S, O’Connor JJ (2016). The effects of hypoxia and inflammation on synaptic signaling in the CNS. Brain Sci.

[68] Musa R, Qurie A (2020 Jan) Raynaud Disease. [Updated 2020 Nov 19]. In: StatPearls. StatPearls Publishing, Treasure Island (FL) Available from: https://www.ncbi.nlm.nih.gov/books/NBK499833 29763008

[69] Mortazavi A, Williams BA, McCue K, Schaeffer L, Wold B (2008). Mapping and quantifying mammalian transcriptomes by RNA-Seq. Nat Methods..

[70] Moussavi-Nik SH (2015). Alzheimer’s disease-related peptide PS2V plays ancient, conserved roles in stimulation of ã-secretase activity and suppression of the unfolded protein response under hypoxia. Human Mol Genet.

[71] Muz B, de la Puente P, Azab F, Azab AK (2015). The role of hypoxia in cancer progression, angiogenesis, metastasis, and resistance to therapy. J Alzheimer Dis.

[72] O’Day DH, Eshak K, Myre MA (2015). Calmodulin binding proteins and Alzheimer’s disease. J Alzheimer Dis.

[73] O'Brien RJ, Wong PC (2011). Amyloid precursor protein processing and Alzheimer's disease. Annu Rev Neurosci.

[74] Papadopoulou MV, Ji M, Bloomer WD (2011). Hypoxia-Dependent Retinal Toxicity of NLCQ-1 (NSC 709257) in BALB/c Mice. Comparison with Tirapazamine. Basic Clin Pharmacol.

[75] Pashaiasl M, Ebrahimi M, Ebrahimie E (2016). Identification of the key regulating genes of diminished ovarian reserve (DOR) by network and gene ontology analysis. Mol Biol Reports..

[76] QiangYY LCZ, Sun R, Zheng LS, Peng LX, Yang JP, Qian CN (2018). Along with its favorable prognostic role, CLCA2 inhibits growth and metastasis of nasopharyngeal carcinoma cells via inhibition of FAK/ERK signaling. J Exp Clin Cancer Res.

[77] Rafiemanesh H (2016). Colorectal cancer in Iran: Epidemiology and morphology trends. EXCLI J.

[78] Ramena G, Yin Y, Yu Y, Walia V, Elble RC (2016). CLCA2 interactor EVA1 is required for mammary epithelial cell differentiation. PLoS One.

[79] Rankin E, Giaccia A (2008). The role of hypoxia-inducible factors in tumorigenesis. Cell Death Differ.

[80] Richardson GD, Laval S, Owens WA (2015). Cardiomyocyte regeneration in the mdx mouse model of nonischemic cardiomyopathy. Stem Cells Dev.

[81] Robinette BL, Harrill JA, Mundy WR, Shafer TJ (2011). In vitro assessment of developmental neurotoxicity: use of microelectrode arrays to measure functional changes in neuronal network ontogeny1. Front Neuroeng.

[82] Schito L, Semenza GL (2016). Hypoxia-inducible factors: master regulators of cancer progression. Trends Cancer.

[83] Sciacqua A, Grillo N, Quero M, Sesti G, Perticone F (2012). Asymmetric dimethylarginine plasma levels and endothelial function in newly diagnosed type 2 diabetic patients. Int J Mol Sci.

[84] Sedger LM, McDermott MF (2014). TNF and TNF-receptors: from mediators of cell death and inflammation to therapeutic giants–past, present and future. Cytokine Growth Factor Rev.

[85] Shariepour Z, Aliakbari BA (2011). The effects of cloudiness and total ozone on UV-B radiation in Esfahan region. Geophy J.

[86] Shlyakhto E (2007). Cellular aspects of pathogenesis of hypertrophic cardiomyopathy: Role of cardiomyocyte polyploidy and activation of nuclear antigen of the proliferating cell in myocardium. Cell Tissue Biol.

[87] Sibal LC, Agarwal SD, Home PH, Boger R (2010). The role of asymmetric dimethylarginine (ADMA) in endothelial dysfunction and cardiovascular disease. Curr Cardiol Rev.

[88] Simon F (2004). Hydroxyl radical activation of a Ca2+-sensitive nonselective cation channel involved in epithelial cell necrosis. Am J of Physiol-Cell Ph.

[89] Sochocka M, Diniz BS, Leszek J (2017). Inflammatory response in the CNS: friend or foe?. Mol Neurobiol.

[90] Solomons HD (2011). Raynaud’s phenomenon. Cardiovascular Journal of Africa.

[91] Stepień K, Dzierzega-Lecznar A, Tam I (2007). The role of neuromelanin in Parkinson's disease--new concepts. Wiad Lek.

[92] Stevenson JW (2016). The amyloid precursor protein of Alzheimer’s disease clusters at the organelle/microtubule interface on organelles that bind microtubules in an ATP dependent manner. PloS One.

[93] Su JL (2006). The VEGF-C/Flt-4 axis promotes invasion and metastasis of cancer cells. Cancer Cell.

[94] Su JL (2007). The role of the VEGF-C/VEGFR-3 axis in cancer progression. Brit J Cancer.

[95] Sung HY, Han J, Ju W, Ahn JH (2016). Synaptotagmin-like protein 2 gene promotes the metastatic potential in ovarian cancer. Oncology Rep.

[96] Svobodová AR (2012). DNA damage after acute exposure of mice skin to physiological doses of UVB and UVA light. Arch Dermatol Res.

[97] Takeuchi T, Watanabe Y, Takano-Shimizu T, Kondo S (2006). Roles of jumonji and jumonji family genes in chromatin regulation and development. Dev Dyn.

[98] Talebian F, Bai XF (2012). The role of tumor expression of CD200 in tumor formation, metastasis and susceptibility to T lymphocyte adoptive transfer therapy. Oncoimmunology.

[99] Tarazona-Santos E (2010). Diversity in the glucose transporter-4 gene (SLC2A4) in Humans reflects the action of natural selection along the old-world primates’ evolution. PloS One.

[100] Terraneo L (2017). Brain adaptation to hypoxia and hyperoxia in mice. Redox Biol.

[101] Thiersch M, Swenson E, Haider T, Gassmann M (2017). Reduced cancer mortality at high altitude: The role of glucose, lipids, iron and physical activity. Exp Cell Res.

[102] Thiersch M, Swenson ER (2018). High altitude and cancer mortality. High Alt Med Biol.

[103] Thompson R, Buttigieg J, Zhang M, Nurse C (2007). A rotenone-sensitive site and H2O2 are key components of hypoxia-sensing in neonatal rat adrenomedullary chromaffin cells. Neurosci.

[104] Tintu A (2009). Hypoxia induces dilated cardiomyopathy in the chick embryo: mechanism, intervention, and long-term consequences. PloS One.

[105] Tousoulis D, Kampoli AM, Tentolouris Nikolaos Papageorgiou C, Stefanadis C (2012). The role of nitric oxide on endothelial function. Curr Vasc Pharmacol.

[106] Twohig JP, Cuff SM, Yong AA, Wang EC (2011). The role of tumor necrosis factor receptor superfamily members in mammalian brain development, function and homeostasis. Rev Neurosci.

[107] Urner M (2012). Effect of hypoxia and dexamethasone on inflammation and ion transporter function in pulmonary cells. Clin Exp Immunol.

[108] Vasiev B, Balter A, Chaplain M, Glazier JA, Weijer CJ (2010). Modeling gastrulation in the chick embryo: formation of the primitive streak. PLoS One.

[109] Wakabayashi K, Tanji K, Mori F, Takahashi H (2007). The Lewy body in Parkinson's disease: Molecules implicated in the formation and degradation of α-synuclein aggregates. Neuropathology.

[110] Wang D, Luo L, Guo J (2014). miR-129-1-3p inhibits cell migration by targeting BDKRB2 in gastric cancer. Med Oncol.

[111] Wang RS, Oldham WM, Loscalzo J (2014). Network-based association of hypoxia-responsive genes with cardiovascular diseases. New J Phys.

[112] Watanabe Y, Kusuoka H, Fukuchi K, Fujiwara T, Nishimura T (1997). Contribution of hypoxia to the development of cardiomyopathy in hamsters. Cardiovasc Res.

[113] Weber AM, Ryan AJ (2015). ATM and ATR as therapeutic targets in cancer. Pharmacol Therapeut.

[114] Winklhofer KF, Haass C (2010). Mitochondrial dysfunction in Parkinson's disease. Biochimica et Biophysica Acta (BBA). Mol Basis Dis.

[115] Wouters A, Pauwels B, Lardon F, Vermorken JB (2007). Implications of in vitro research on the effect of radiotherapy and chemotherapy under hypoxic conditions. Oncologist.

[116] Yan MH, Wang X, Zhu X (2013). Mitochondrial defects and oxidative stress in Alzheimer disease and Parkinson disease. Free Radical Bio Med.

[117] Yel M, Güven T, Türker H (2014). Effects of ultraviolet radiation on the stratum corneum of skin in mole rats. J Radiat Res Appl Sci.

[118] Zhang J, Darley-Usmar V (2012) Mitochondrial dysfunction in neurodegenerative disease: Protein aggregation, autophagy, and oxidative stress. In: Mitochondrial dysfunction in neurodegenerative disorders Springer, London, pp 95-111.

[119] Zhang K (2018). Investigation of hypoxia networks in ovarian cancer via bioinformatics analysis. J Ovarian Res.

[120] Zucca FA (2017). Interactions of iron, dopamine and neuromelanin pathways in brain aging and Parkinson's disease. Prog Neurobiol.

