# Supplementary material for: Gene network analysis to determine the effect of hypoxia-associated genes on brain damages and tumorigenesis using an avian model
Source: J Genet Eng Biotechnol. 2021 Jul 8;19:100. doi: 10.1186/s43141-021-00184-5 (PMC8266987; doi:10.1186/s43141-021-00184-5)
Supplement: Supplementary file 1 — Supplementary material [file 43141_2021_184_MOESM1_ESM.doc]

**Results**

**Quality control**

Results of the quality control indicate that short reads were sequenced based on an appropriate form. Trimming was carried out according to the Phred score and the nucleotide contribution. Removing at least ten primary bases, we trimmed 3′ side of short reads in order to minimize mapping errors. In addition, 5% of reads that contained the lowest Phred scores were also removed.

**Phenotypic data, phylogenetic and population structure analysis**

Results of principle component analysis indicated that six components (combinations of traits) characterized 92 percent of the total variance; therefore, these components were selected in order to be utilized in the discriminate analysis (Table 1). Results of the discriminate analysis showed that collected lowland and highland samples can be classified into two separated groups, phenotypically. The accuracy was estimated to be 75% (Table 2).

Nei’s genetic distance between highland and lowland populations was estimated to be 3 percent. Thus, results of phylogenic analysis revealed that there was no significant genetic distance between highland and lowland populations.

The analysis of genetic population structure was carried out by multiple correspondence analysis (MCA) based on 10000 SNVs genotypes. The first two components of variants were 29.5% and 26.5%, respectively. Results indicated that highland chickens were classified closely; while lowland chickens were classified together. Consequently, highland and lowland chickens could be classified into two groups (Figure 1).

**Mapping, variants discovery and statistical analysis**

The mapping efficiency versus reference genome was estimated between 94% - 97% for samples (Table 3). Parameters were optimized in order to decrease false variants for the variants discovery, (See Supplementary Table S1 online for optimization process). The process of optimization was carried out in three steps, and parameters of the step 3 were selected in order to be utilized in the variants detection.

We found that more than 20 million variations were generated, including single nucleotide variant (SNV), multi nucleotide variant (MNV), insertion, deletion and replacement (See Supplementary Table S2 online). Results of the statistical analysis indicated that there was a significant (*P* < 0.001) difference of variant distribution between highland and lowland samples (Table 4). Thus, we can realize that harsh environmental conditions, including the lack of oxygen, impacted on the variant distribution. In addition, the Chi-square test was carried out for each chromosome, separately. Most of chromosomes had significant differences in harboring associated variants with altitude alteration; therefore, those chromosomes that do not have significant differences are given in Table 5, including mtDNA, W, 4, 11, and 32.

**Differential variants** **of** **highland and lowland ecotypes**

In order to remove the common variants between highland and lowland chickens*,* total variants of those samples were compared*.* Differential variants were also kept in order to be utilized in further analyses (Table S3). Finally, the frequency thresholds of 50% and 100% were determined in order to carry out comparisons between the groups of males and females (Table S4). The threshold frequency is considered as the percentage of samples that contain variants. Generally, 97610 and 17024 variants were detected for both males and females as the differential variants of highland and lowland chickens, respectively. For the first time, 16623 and 3024 variants were identified as new variants in males and females. Results of filtering in the male birds, based on the overlap, showed that 614 and 96995 variants were located in coding and non-coding regions. We found that 202 out of 614 coding variants will lead to amino acid changes. It was observed that fewer coding and non-coding variants were detected among female birds (Table 6).

Totally, more than 30 GO terms were reported for differential variants in females. Considering the molecular functions, the most frequency of GO terms belonged to 5'-3' exodeoxyribonuclease activity and histone binding, respectively. Furthermore, candidate genes of GO terms were suggested including *DCLRE1C* and *ATAD2*. It was also found that considering cellular components, the mitochondrion was identified as the most important organelle for hypoxia condition differing between highland and lowland ecotypes.

Results of biological process analysis showed that most of the GO terms were related to the DNA repair processes including double-strand break repair and inter strand cross-link repair.

Results of gene ontology enrichment among males indicated that several candidate genes such as *TATDN1*, *POLQ* and *DCLRE1A* were associated with DNA repair. It was observed in the biological process analysis that there were several Go terms, including pericardium morphogenesis and thalamus development. There were also several biological pathways suggested for DNA repair and biosynthetic (e.g., DNA biosynthetic process; double-strand break repair; DNA repair; and inter strand cross-link repair). The NK T cell differentiation was reported as a biological pathway of immune response in the hypoxia condition. To see more results of gene ontology enrichments analyses in males and females, refer to the Tables 7 and 8.

**Common variants between highland and lowland chickens**

Generally, in both males and females, 53780 variants were shared between highland and lowland chickens; 17835 new variants were also reported for the first time. It was found that 516 out of 53780 variants could lead to changes in amino acid sequences (Supplementary Table S5 online).

Results of gene ontology enrichment analysis of detected variants, in females and males, indicated that GO terms were related to the cell survival. For example, cell proliferation, cell growth (*ROS1)*, digestion (*PRSS3)*, chromosome organization (*BRCA2)*, RNA processing *(TDRD9)*, telomere capping (*POT1)*, and cytokinesis *(CEP55)*. In order to see more details of common variants, refer to the Supplementary text and Supplementary Tables S6-S7 online.

**mtDNA variants**

In general, for both highland and lowland chickens, 50 variants were detected in mtDNA (10 samples), and most of them were considered as the novel variants (Table 9). One significant gene ontology (GO term: 1902600) was observed in mtDNA variants. It was reported that, for highland chickens, hydrogen ion transmembrane transport was a biological pathway in males. It is involved in the directed movement of hydrogen ion (proton) across a membrane. Noticeably, *COX3* gene (cytochrome c oxidase III) was considered in this biological pathway.

**Validations of novel differential SNVs between highland and lowland ecotypes and mtDNA variants**

Results of the validation indicated that 19 out of 32 novel variants were validated in mtDNA. Five variations of regions including 5928, 6758, 8070, 8330, 11378 had the most validating percentage of mtDNA (Table 10). Results of the validation of novel differential SNVs showed that 21 out of 3845 variants were validated in five new samples (Table 10).

**The analysis of genomic variants enrichment for the differential variant between highland and lowland chickens**

The chromosome that had the most standard frequency in females was known as the chromosome 27 (Fig 2). In fact, 234 variants were recognized on chromosome 27; however, 100 variants (43% of variants) were located in the range of 2692-1×10^6^ base pairs (16% of the chromosome length) (Fig 3a). Findings illustrated that 40 ENSGALT (Ensembl transcript) IDs were available in this region, and five Ensembl gene IDs were also detected by BioMart tools (Table 11). We found that MIR6644-2 gene contributed in the gastrulation. Gastrulation is recognized as one of the most important steps in animals’ embryonic development. Furthermore, the single layered blastula develops to a multilayered organization, called gastrula, during the gastrulation ^17^.

The most standard frequency of variants of chromosome 2 in males was estimated to be 119.60 (Fig 2). A total of 17891 variants was detected on chromosome 2, but 7600 variants (43%) were located in the range of 41×10^6^-81×10^6^(27% of chromosome length) base pairs; however, this region, which had a high density of variation, was selected for further analysis (Fig 3b). It was shown by results of UCSC and the table browser analysis that 412 ENSGALT IDs were available for the selected region and consequently, nine Ensemble gene IDs were identified by BioMart tools (Table 11). We found that some functions were involved in the adaptation to hypoxia including chemotaxis, immune response, inflammatory response, and positive regulations of monocyte chemotaxis. Other results indicated that Jumonji protein might have an important role in the adaptation to hypoxia. Reviewing the studies, we found that Jumonji protein family would contribute in the wide range of chromatin regulation, genes expression, and signaling pathways^18^. While the Jumonji is known as the DNA binding and transcriptional repressor, this protein interacts with the Polycomb repressive complex 2 (*PRC2*) in humans, which plays a critical role in the regulation of gene expression during the embryonic development (www.ncbi.nlm.nih.gov/gene/3720). This study indicates that *PRL* gene can play a very important role in the condition of lack of oxygen. It has been illustrated that the *PRL* gene is associated with reproductive traits; specially egg production traits^19^. In fact, *PRL* gene is a multifunctional hormone that impacts on multiple physiological processes including cardiovascular system; however, they have been poorly described^20^.

**The possible gene network of differential variants**

Results of the analysis of gene network indicate that seven differential candidate genes contribute in the regulation of carcinogenesis. It is observed that most of the candidate genes induce carcinogenesis, especially *BMX* and *LRP6* (Figure 4). However, *HSPA14* and *DCLRE1A* genes had an inhibition impact on the tumorigenesis. See the Supplementary Table S8 online for more details about underlying references of relationships. Figure 5 shows the cellular location of candidate gene. For example, *LRP6* is considered as the receptor; therefore, it is shown in the membrane cell. In addition, *BMX* and *HSPA14* were activated in cytoplasm, but other candidate genes were in the nucleus. This information could be useful in order to explore the contribution of genes in biological pathways. For instance, *DCLRE1A* and *PRKDC* have a critical role in the process of DNA repair^21^. Therefore, their location can also be observed in nucleus. These results could be helpful in order to understand the process of diseases creation and select the appropriate drug design method, based on the cellular location of key proteins in carcinogenesis.

**The submission of novel variants to European Bioinformatics Information (EBI)**

Many SNVs are detected as the novel variants in this investigation. Novel variants were submitted to EBI database with the accession number of PRJEB24944. See Table S9 for more details. Discovered variants can be utilized for future breeding programs. The analysis of data with hierarchical pattern recognition algorithms can be ranked, thus the best combination of variants distinguishing highland and lowland chickens will also be found ^22, 23^.

**Materials and Methods**

The summery of sampling and whole genome data analyses are depicted in Figure 7.

**Ethical approval statements**

This investigation is in accordance with relevant guidelines and regulations of Shiraz University. All experimental protocols were approved by Institute of Biotechnology at Shiraz University.

The whole procedure of blood sampling was approved by the Department of Animal Science at Shiraz University (Permit number: 93-192). No birds were slaughtered and harmed.

**Blood sampling and DNA extractions**

Blood samples were collected from Isfahan (highland, altitude=2087m) and Mazandaran (lowland, altitude=54m) provinces. Ten samples including three males and two females of the highland, and two males and three females of the lowland were collected. See Figure 8 for more details about sampling locations. Two mL of blood was obtained from the birds’ wing vein. Using salting out protocol, the total DNA was isolated from the whole blood^69^. The process of testing DNA samples’ quality was carried out by the agarose gel (2%) and NanoDrop spectrophotometer, and high-quality DNA samples were also utilized for the subsequent whole genome resequencing.

**Phenotypic data and statistical analysis**

Classification of highland and lowland chickens, based on phenotypic traits, was the main purpose of the statistical analysis. Twenty-four quantitative traits were recorded for 16 chickens of highland and lowland. To reduce measurement error, all chickens were matured while recording. The same recording protocol was also used in order to measure phenotypic traits of all the birds. Traits included the body weight (gr), neck length, body size (between waist and pectoral circumference), shank length, body size (between waist and abdominal circumference), wing length, tail length, femur length, crown length, crown height, back cape length, body size (between pectoral and cloaca circumference), head height (height from head to floor), wings length (open wings), number of toes, number of spur (nail of the foot), beak length, head circumference with a crown and a double wattles, toes length (right, middle, left), wattle height, wattle width, pectoral width, femur diameter and shank diameter. Utilizing the principle component analysis and discriminate analysis in Minitab software, we analyzed these traits (version: 17)^70^.

**Whole genome sequencing**

The construction of genomic libraries was based on the Illumina standard genome library preparation pipeline. Using Hiseq2000 platform, whole genomes were sequenced and the length of provided paired-end short reads was also125 bp.

**Quality control and trimming**

The function of quality control in CLC Genomic Workbench (8.5.1)^71^ was used by the following parameters for each sample: length distribution, GC content, ambiguous base content, Phred score, nucleotide contribution, enrich 5 mers, and duplicate sequences^72^. The adaptor sequences were removed by Illumina Company; thereby, trimming was carried out based on other parameters.

**Reference genome, mapping, locally realignments and coverage analysis**

The reference genome and annotations were downloaded from the Ensembl database. Annotations included gene annotations and variants (<ftp://ftp.ensembl.org/pub/relase-84/fasta/gallus_gallus>). Mapping was carried out in CLC Genomics Workbench (8.5.1) ^71^ based on the following parameters: masking mode= no masking, mismatch cost = 2, cost of insertions and deletions = linear gap cost, insertion cost = 3, deletion cost = 3, length fraction = 0.7, similarity fraction = 0.8, global alignment = no, Auto-detect paired distances = yes, and non-specific match handling = ignore. Finally, local realignment was used according to the following parameters: realign-unaligned ends = yes and multi-pass realignment = 2 ^73^.

The analysis of coverage was also utilized in order to identify the frequency of a base’s coverage in the reference genome by the mapped read of a given sequencing ^74^.

Here, the analyses of coverage were estimated for all highland and lowland samples before and after the trimming process. Also, Lander/Waterman equation was used for the calculation of the coverage ^75^.

C = LN / G

Where, C stands for coverage, G is the haploid genome length (bp), L is the read length and N is the number of reads.

**Variant calling and statistical analysis**

The variant detections algorithm was used by CLC genomics workbench (8.5.1) ^71^. First, parameters were optimized for variant calling; therefore, some parameters were changed and tested for the optimization. Decreasing false variants was the main purpose of optimization (Supplementary Table S1online). The ploidy level is fixed in chickens (2n=78). Therefore, fixed ploidy algorithm^76^ was used for variant calling based on following parameters: required variant probability (%) = 95.0, ignore positions with coverage above = 50,000, restrict calling to target regions = not set, ignore broken pairs = yes, ignore non-specific matches = reads, minimum coverage = 10, minimum count = 2, minimum frequency (%) = 30, base quality filter = Yes, neighborhood radius = 15, minimum central quality = 30, minimum neighborhood quality = 25, remove pyro-error variants = yes, in homo-polymer regions with minimum length = 3-with frequency below = 0.8 ^72,76^.

We used the IDEG6 website for the statistical analysis^77^. Using Fisher exact test, we analyzed differences in variants distribution. The statistical analysis was carried out for each variant between highland and lowland samples, separately. Also, Chi-square test was established in order to analyze the differential distributed variations between highland and lowland chickens for each chromosome incorporating the sex of birds in the statistical analysis.

**Phylogenetic and structural population analysis**

Any change in mitochondrial DNA (mtDNA) can be utilized as the phylogenic analysis, because mtDNA are considered as the conserved sequences. A total of 48 SNVs (single nucleotide variations) was identified in mtDNA using CLC Genomic Workbench (8.5.1)^71^, 480 SNVs were also genotyped (see results part) for ten samples. The phylogenic analysis was carried out based on 480 SNVs genotypes, Nei’s genetic distance matrix, and UPGMA method by PopGene software (version 1.32) ^78^.

The population structural analysis was performed using multiple correspondence analysis (MCA) by R program (version: 3.2.2)^79^ and the “Ca” package ^80^) in order to classify the highland and lowland chickens. We used the multiple correspondence analyses for qualitative data, while the principal component analysis (PCA) was used for quantitative data. MCA is another tool of multivariate methods that allows the analysis of systematic patterns of variations with categorical data^81^. 1000 SNVs genotypes were collected randomly from each highland and lowland chickens and a total of 10000 SNVs genotypes was used for MCA analysis.

**Differential variants**

**Comparing variants, filter variants and gene ontology enrichment analysis**

After variants calling, variations of highland chickens were compared against the reads of lowland chickens as a control tool in order to remove the common variation between lowland and highland samples. Also, a comparison was carried out between male and female birds of highland and lowland, separately. We considered five frequency threshold percentages (0, 25, 50, 75 and 100) in order to be utilized for the frequency threshold optimization. The threshold frequency is considered as the percentage of samples that have variants. For instance, determining it to 50% indicates that at least 50% of samples, which were selected as the inputs, must contain a given variant in order to be reported in the output.

The differential variants were filtered based on known variants for the identification of novel variants. CDS (coding region) annotation was selected for overlap comparisons in order to understand which variants were located on coding and non-coding regions. Coding variants were collected for functional consequences and amino acid changes analysis. Reference genome, CDS and mRNA annotations were used for the amino acid change analysis. In addition, synonymous variants and CDS regions that had no variants were also filtered. In order to carry out the amino acid change analysis, standard genetic code was selected in CLC genomics^82^. The file of gene ontology (GO) association, which includes the gene names and associated gene ontology terms, was downloaded from the gene ontology consortium (<http://geneontology.org/>) and imported to CLC Genomic Workbench (8.5.1) ^71^. The output of amino acid change analysis was utilized for the analyses of gene ontology enrichment of the biological process, molecular function, and cellular component. The significance level of GO analysis was determined to be 0.01.

**Common variants discovery between highland and lowland chickens**

The detection of common variants, shared between highland and lowland chickens is the main purpose of common variants discovery. See the Figure 9 for more details about common variants detection. 100% was determined for the frequency threshold. In the other words, all differential variants were removed completely, and common variants were kept for further analysis. The process of filtering and gene ontology enrichment analyses was same as what described for differential variants.

**mtDNA variants**

Ignoring the mtDNA variants in discovery variant projects is very difficult. The analyses of mtDNA variants were carried out separately in this investigation. After variants detection, all mtDNA variants were obtained in highland and lowland chickens, separately. Filtrations and gene ontology enrichments were carried out same as what described for differential variants.

**Linking variants to protein structure**

Results of gene ontology enrichment analyses were applied for variants visualization. Some variants impact on a protein’s amino acid composition. Therefore, the protein’s three-dimensional structure was downloaded by CLC Genomic Workbench (8.5.1) ^71^ from the protein data bank (www.rcsb.org) in order to show the variants location on the protein structure. Variants visualization was carried out for important differential candidate genes of our investigation’s results, or the ones that have their structures in the protein database structure.

**Developing a new approach for genomic variants enrichment analysis**

The identification of genomic regions that contained the highest density of differential variant between highland and lowland chickens, including both males and females, is the main purpose of genomic variants enrichment analysis. In this article, we proposed a plan to analyze the genomic variants enrichment based on the following steps. First, we estimated each chromosome’s standard frequencies of differential variants using the division of variants number by the chromosome length (Mb). Second, the chromosome that had the most standard frequency was selected, and the cumulative frequencies graph of variants was drawn along the chromosome by the R program (version 3.2.2)^79^. Third, chromosome region, which had the most cumulative frequency, was identified and ENSGALT (Ensembl transcript) IDs were also detected by UCSC and Table browser ([www.genome.ucsc.edu/cgi-bin/hgTables](http://www.genome.ucsc.edu/cgi-bin/hgTables)) and finally, all detected ENSGALT IDs were converted into the ENSGAL gene ID by BioMart ([www.ensembl.org/biomart](http://www.ensembl.org/biomart)).

**Validations**

Variants discovery projects produce numerous variations. Thereby, validating the variants is highly required. Here, validations were carried out for two groups of detected variants. First, novel differential SNVs between highland and lowland chickens, and second, mtDNA variations in highland chickens. Thus, the sequencing of other five whole genomes of highland samples (Isfahan) was carried out, separately. The whole genome sequencing, trimming, and variant detection were performed according to the information provided in the material and method sections. A total of 3845 SNVs was reported as new variants for differential variants analyses and 32 variations were also detected in mtDNA for highland samples (see result part). Each SNV’s regions and chromosomes were evaluated in the new five samples by R program in order to validate the variants^79^. The percentage of validation was calculated based on those samples that had variants.

**Data availability**

Produced and analyzed datasets of this study are not publicly available due to the policy of financial sponsor. But datasets are available from the corresponding author on reasonable request. In addition, novel detected SNVs with an accession number of PRJEB24944 are available in EBI.

**References**

1. Tixier-Boichard, M., Bed’hom, B.&Rognon, X. Chicken domestication: From archeology to genomics. *C R Biol.* **334**, 197-204 (2011).

2. Burt, D. W. Chicken genome: current status and future opportunities. *Genome Res.* **15,** 1692-1698 (2005).

3. Meydan, H., Jang, C. P., Yıldız, M. A.&Weigend, S. Maternal origin of Turkish and Iranian native chickens inferred from mitochondrial DNA D-loop sequences. *Asian Austral J Anim Sci.* **29,**1547 (2016).

4. Shahbazi, S., Mirhosseini, S. Z.&Romanov, M. N. Genetic diversity in five Iranian native chicken populations estimated by microsatellite markers. *Biochem Genet.* **45**, 63-75 (2007).

5. Barba, M., Czosnek, H.&Hadidi, A. Historical perspective, development and applications of next-generation sequencing in plant virology. *Viruses.* **6**, 106-136 (2014).

6. Yan, Y., Yi, G., Sun, C., Qu, L.&Yang, N. Genome-wide characterization of insertion and deletion variation in chicken using next generation sequencing. *PloS One* **9**, e104652 (2014).

7. Grocott, M., Montgomery, H.&Vercueil, A. High-altitude physiology and pathophysiology: implications and relevance for intensive care medicine. *Crit Care.***11**, 203-208 (2007).

8. De, B. et al. Systems biology approach to study the high altitude adaptation in tibetans. *Braz Arch Biol Techn.* **56**, 53-60 (2013).

9. Jia, C. et al. Gene Co-Expression Network Analysis Unraveling Transcriptional Regulation of High-Altitude Adaptation of Tibetan Pig. *PloS One* **11**, e0168161 (2016).

10. Dong, K. et al. Genomic scan reveals loci under altitude adaptation in Tibetan and Dahe pigs. *PLoS One* **9**, e110520 (2014).

11. Srivastava, S. et al. Association of polymorphisms in angiotensin and aldosterone synthase genes of the renin–angiotensin–aldosterone system with high-altitude pulmonary edema. *J. Renin Angiotensin Aldosterone Syst.***13**, 155-160 (2012).

12. Michiels, C. Physiological and pathological responses to hypoxia. *Am J Pathol.* **164**, 1875-1882 (2004).

13. Takiyama, Y.&Haneda, M. Hypoxia in diabetic kidneys. *BioMed Res Int.* **2014,** doi:10.1155/2014/837421 (2014).

14. Stobdan, T., Karar, J.&Pasha, M. Q. High altitude adaptation: genetic perspectives. [*High Alt Med Biol*](https://www.ncbi.nlm.nih.gov/pubmed/18578645)*.* **9**, 140-147 (2008).

15. Zhang, Q. et al. Genome resequencing identifies unique adaptations of Tibetan chickens to hypoxia and high-dose ultraviolet radiation in high-altitude environments. *Genome Biol Evol.* **8**, 765-776 (2016).

16. Zhao, X. et al. High-altitude adaptation of Tibetan chicken from MT-COI and ATP-6 perspective. *Mitochondrial DNA.* **27**, 3280-3288 (2016).

17. Vasiev, B., Balter, A., Chaplain, M., Glazier, J. A.&Weijer, C. J. Modeling gastrulation in the chick embryo: formation of the primitive streak. *PLoS One* **5**, e10571 (2010).

18. Takeuchi, T., Watanabe, Y., Takano‐Shimizu, T.&Kondo, S. Roles of jumonji and jumonji family genes in chromatin regulation and development. *Dev Dyn.* **235**, 2449-2459 (2006).

19. Wilkanowska, A., Mazurowski, A., Mroczkowski, S.&Kokoszyński, D. Prolactin (PRL) and prolactin receptor (PRLR) genes and their role in poultry production traits. *Folia biologica.* **62**, 1-8 (2014).

20. Hsieh, D. J.-Y. et al. Prolactin protects cardiomyocytes against intermittent hypoxia-induced cell damage by the modulation of signaling pathways related to cardiac hypertrophy and proliferation. *. Int J Cardiol.* **181**, 255-266 (2015).

21. Felgentreff, K. et al. Functional analysis of naturally occurring DCLRE1C mutations and correlation with the clinical phenotype of ARTEMIS deficiency. *J Allergy Clin Immunol.* **136**, 140-150. e7 (2015).

22. Ebrahimie, E., Ebrahimi, F., Ebrahimi, M., Tomlinson, S.&Petrovski, K. R. Hierarchical pattern recognition in milking parameters predicts mastitis prevalence. *Comput Electron Agr.* **147**, 6-11 (2018).

23. Sharifi, S. et al. Integration of machine learning and meta-analysis identifies the transcriptomic bio-signature of mastitis disease in cattle. *PloS One* **13**, e0191227 (2018).

24. Hartmut, M. Cytochrome c oxidase: catalytic cycle and mechanisms of proton pumping-A discussion. *Biochem*. **38,** 15129–15140 (1999).

25. Sun, J. S., Zhong, H., Chen, S. Y., Yao, Y. G., & Liu, Y. P. Association between MT-CO3 haplotypes and high-altitude adaptation in Tibetan chicken. *Gene*. **529,** 131–137(2013).

26. Camus, M.F., Wolf, J.B., Morrow, E.H., Dowling, D.K. Single Nucleotides in the mtDNA Sequence Modify Mitochondrial Molecular Function and Are Associated with Sex-Specific Effects on Fertility and Aging. *Curr Biol*. **25,** 2717-2722 (2015).

27. Mossman, J. A., Tross, J. G., Li, N., Wu, Z., Rand, D. M. Mitochondrial-Nuclear Interactions Mediate Sex-Specific Transcriptional Profiles in Drosophila. *Genetics*. 204, 613-630 (2016).

28. Lemarie, A.&Grimm, S. Mitochondrial respiratory chain complexes: apoptosis sensors mutated in cancer? *Oncogene.* **30**, 3985 (2011).

29. Zhou, F., Yin, Y., Su, T., Yu, L.&Yu, C.-A. Oxygen dependent electron transfer in the cytochrome bc1 complex. *Biochimica et Biophysica Acta (BBA)-Bioenergetics.* **1817**, 2103-2109 (2012).

30. Liu, L., Simon, M.C. Regulation of Transcription and Translation by Hypoxia. *Cancer Biol Ther.* **3,** 492-497 (2004).

31. Koritzinsky, M., Wouters, B.G. Hypoxia and regulation of messenger RNA translation. *Methods Enzymol*. **435**, 247-273 (2007).

32. Staudacher, J.J. et al. Hypoxia-induced gene expression results from selective mRNA partitioning to the endoplasmic reticulum. *Nucleic Acids Res*. **43,** 3219–3236 (2015).

33.Tamagnone, L. et al. BMX, a novel nonreceptor tyrosine kinase gene of the BTK/ITK/TEC/TXK family located in chromosome Xp22. 2. *Oncogene* **9**, 3683-3688 (1994).

34. Paavonen, K. et al. Bmx tyrosine kinase transgene induces skin hyperplasia, inflammatory angiogenesis, and accelerated wound healing. [*Mol Biol Cell*](https://www.ncbi.nlm.nih.gov/pmc/articles/PMC515354/)*.* **15**, 4226-4233 (2004).

35. McNamee, E. N., Johnson, D. K., Homann, D.&Clambey, E. T. Hypoxia and hypoxia-inducible factors as regulators of T cell development, differentiation, and function. *Immunol Res.* **55**, 58-70 (2013).

36. Xia, X. et al. Integrative analysis of HIF binding and transactivation reveals its role in maintaining histone methylation homeostasis. *Proceedings of the National Academy of Sciences* **106**, 4260-4265 (2009).

37. Niu, X. et al. The von Hippel–Lindau tumor suppressor protein regulates gene expression and tumor growth through histone demethylase JARID1C. *Oncogene.* **31**, 776 (2012).

38. Hancock, R. L., Dunne, K., Walport, L. J., Flashman, E.&Kawamura, A. Epigenetic regulation by histone demethylases in hypoxia. *Epigenomics.* **7**, 791-811 (2015).

39. Zhang, H.&Burggren, W. Hypoxic level and duration differentially affect embryonic organ system development of the chicken (Gallus gallus). *Poult Sci.* **91**, 3191-3201 (2012).

40. Mattiesen, W.-R. C. et al. Increased neurogenesis after hypoxic-ischemic encephalopathy in humans is age related. *Acta neuropathological.* **117**, 525-534 (2009).

41. Patterson, A.J., Zhang, L. Hypoxia and fetal heart development. *Curr Mol Med*. **10,** 653–666 (2010).

42. Joiner, D. M., Ke, J., Zhong, Z., Xu, H. E.&Williams, B. O. LRP5 and LRP6 in development and disease. *Trends Endocrinol Metab.* **24**, 31-39 (2013).

43. Svobodová, A. R. et al. DNA damage after acute exposure of mice skin to physiological doses of UVB and UVA light. *Arch Dermatol Res.***304**, 407-412 (2012).

44. Yel, M., Güven, T.&Türker, H. Effects of ultraviolet radiation on the stratum corneum of skin in mole rats. *J. Radiat Res Appl.* **7,** 506-511 (2014).

45. Zhang, B. et al. A Comprehensive MicroRNA Expression Profile Related to Hypoxia Adaptation in the Tibetan Pig. *PloS One* **10**, e0143260 (2015).

46. Blick, C. et al. Identification of a hypoxia-regulated miRNA signature in bladder cancer and a role for miR-145 in hypoxia-dependent apoptosis. *Brit J Cancer.***113**, 634 (2015).

47. Kulshreshtha, R. et al. A microRNA signature of hypoxia. *Mol Cell Biol.* **27**, 1859-1867 (2007).

48. Shao, P. et al. Drastic expression change of transposon-derived piRNA-like RNAs and microRNAs in early stages of chicken embryos implies a role in gastrulation. *RNA Biol.***9**, 212-227 (2012).

49. Ghatpande, S. K., Billington, C. J., Rivkees, S. A.&Wendler, C. C. Hypoxia induces cardiac malformations via A1 adenosine receptor activation in chicken embryos. *Birth Defects Res A Clin Mol Terato.* **82**, 121-130 (2008).

50. Lo, K. H., Hui, M. N. Y., Yu, R. M. K., Wu, R. S. S.&Cheng, S. H. Hypoxia impairs primordial germ cell migration in zebrafish (Danio rerio) embryos. *PLoS One* **6**, e24540 (2011).

51. Chakraborty, C., Hsu, C. H., Wen, Z. H., Lin, C. S.&Agoramoorthy, G. Zebrafish: a complete animal model for in vivo drug discovery and development. *Curr Drug Metab.***10**, 116-124 (2009).

52. Burt, D. W. Emergence of the chicken as a model organism: implications for agriculture and biology. *Poult Sci.***86**, 1460-1471 (2007).

53. Dodgson, J. B.&Romanov, M. N. Use of chicken models for the analysis of human disease. *Curr Protoc Hum Genet.* 15.5. 1-15.5. 12 (2004).

54. Mathieu, A.-L. et al. PRKDC mutations associated with immunodeficiency, granuloma, and autoimmune regulator–dependent autoimmunity. *J Allergy Clin Immunol* **135**, 1578-1588. e5 (2015).

55. Blunt, T. et al. Identification of a nonsense mutation in the carboxyl-terminal region of DNA-dependent protein kinase catalytic subunit in the scid mouse. *Proceedings of the National Academy of Sciences* **93**, 10285-10290 (1996).

56. Xie, W., Su, Y.-h., Feng, Q., Qu, L.-k.& Shou, C.-c. Inhibitory effects of silencing PES1 gene expression on the malignant phenotypes of colon cancer cells. *Tumor.* **35**, 1175-1184 (2015).

57. Li, J. et al. Repression of PES1 expression inhibits growth of gastric cancer. *Tumor Biol.* **37**, 3043-3049 (2016).

58. Brennan, M., Lim, B. The Actual role of receptors as cancer markers, biochemical and clinical aspects: receptors in breast cancer. *Adv Exp Med Biol*. **867,** 327-337 (2015).

59. Bar-Shavit, R. et al. G protein-coupled receptors in cancer. *Int J Mol Sci.* **17**, 1320 (2016).

60. Li, Y., Lu, W., He, X., Schwartz, A. L.&Bu, G. LRP6 expression promotes cancer cell proliferation and tumorigenesis by altering β-catenin subcellular distribution. *Oncogene.* **23**, 9129 (2004).

61. Liu, C.-C., Prior, J., Piwnica-Worms, D.&Bu, G. LRP6 overexpression defines a class of breast cancer subtype and is a target for therapy. *Proceedings of the National Academy of Sciences.* **107**, 5136-5141 (2010).

62. Ma, J., Lu, W., Chen, D., Xu, B.&Li, Y. Role of Wnt Co‐Receptor LRP6 in Triple Negative Breast Cancer Cell Migration and Invasion *J Cell Biochem.* **118**, 2968-2976 (2017).

63. Tung, E. K.-K., Wong, B. Y.-C., Yau, T.-O.&Ng, I. O.-L. Upregulation of the Wnt co-receptor LRP6 promotes hepatocarcinogenesis and enhances cell invasion. *PloS One* **7**, e36565 (2012).

64. Calderwood, S. K., Stevenson, M. A.&Murshid, A. Heat Shock Proteins, Autoimmunity, and Cancer Treatment. *Autoimmune Dis*. doi:10.1155/2012/486069 (2012).

65. Lianos, G. D. et al. The role of heat shock proteins in cancer. *Cancer letters.* **360**, 114-118 (2015).

66. Yang, Z. et al. Upregulation of heat shock proteins (HSPA12A, HSP90B1, HSPA4, HSPA5 and HSPA6) in tumour tissues is associated with poor outcomes from HBV-related early-stage hepatocellular carcinoma. *Int J Med Sci.* 12, 256-263 (2015).

67. Wu, J. et al. Heat shock proteins and cancer. *Trends Pharmacol Sci.* 38, 226-256 (2017).

68. Rerole, A. L., Jego, G., Garrido, C. Hsp70: anti-apoptotic and tumorigenic protein. *Methods Mol Biol*. 787, 205-30 (2011).

69. Iranpur-Mobarakeh, V. Esmailizadeh, A, K. Rapid Extraction of High Quality DNA from Whole Blood Stored at -4 C for Long Period. *Protocol Online*. <http://www.protocol-online.org> (2010).

70. Minitab 17 Statistical Software (2010). [Computer software]. State College, PA: Minitab, Inc. ([www.minitab.com](http://www.minitab.com))

71. CLC Genomics Workbench 8.5.1 (<https://www.qiagenbioinformatics.com/>).

72. Doan, R. et al. Whole-genome sequencing and genetic variant analysis of a Quarter Horse mare. *BMC genomics.* **13**, DOI: 10.1186/1471-2164-13-78. (2012).

73. Mortazavi, A., Williams, B. A., McCue, K., Schaeffer, L.&Wold, B. Mapping and quantifying mammalian transcriptomes by RNA-Seq. *Nat Methods.***5**, 621-628 (2008).

74. Sims, D., Sudbery, I., Ilott, N. E., Heger, A.&Ponting, C. P. Sequencing depth and coverage: key considerations in genomic analyses. *Nat Rev Genet.* **15**, 121-132 (2014).

75. Lander, E. S. &Waterman, M. S. Genomic mapping by fingerprinting random clones: a mathematical analysis. *Genomics.* **2**, 231-239 (1988).

76. Dias, M. et al. SNP detection using RNA-sequences of candidate genes associated with puberty in cattle. *Genet Mol Res.* **16,** doi: 10.4238/gmr16019522 (2017).

77. Romualdi, C., Bortoluzzi, S., d’Alessi, F.&Danieli, G. A. IDEG6: a web tool for detection of differentially expressed genes in multiple tag sampling experiments. *Physiol Genomics.***12**, 159-162 (2003).

78. Yeh, F. C., Yang, R. C., Boyle, T. B., Ye, Z.&Mao, J. X. POPGENE, the user-friendly shareware for population genetic analysis. *Molecular biology and biotechnology centre, University of Alberta, Canada* **10**, 295-301 (1997).

79. R Core Team (2014). R: A language and environment for statistical computing. R Foundation for Statistical Computing, Vienna, Austria. URL <http://www.R-project.org/>.

80. Nenadic, O.&Greenacre, M. Correspondence analysis in R, with two-and three-dimensional graphics: The ca package. *J Stat Softw.* **20,** doi:10.18637/jss. v020.i03 (2007).

81. Abdi, H. &Valentin, D. Multiple correspondence analysis. *Encyclopedia of measurement and statistics*, 651-657 (2007).

82.Comparative analysis of three bovine genomes. [www.qiagenbioinformatics.com/support/tutorials](http://www.qiagenbioinformatics.com/support/tutorials) (2017).

83. Pashaiasl, M., Ebrahimi, M.&Ebrahimie, E. Identification of the key regulating genes of diminished ovarian reserve (DOR) by network and gene ontology analysis. *Mol Biol Rep.* **43**, 923-937 (2016).

**Table 1: Principle component analysis of phenotypic traits for discriminate analysis and clustering the highland and lowland populations**

| Component | 1 | 2 | 3 | 4 | 5 | 6 |
| --- | --- | --- | --- | --- | --- | --- |
| Eigenvalue | 6.45 | 2.64 | 2.07 | 1.14 | 0.79 | 0.69 |
| Proportion | 0.43 | 0.17 | 0.13 | 0.07 | 0.05 | 0.04 |
| Cumulative | 0.43 | 0.60 | 0.74 | 0.82 | 0.87 | **0.92** |

Twenty-four quantitative traits were measured on 16 highland and lowland chickens by our investigation. In order to reduce the measurement error, all chickens were mature while recording and the same recording protocol was also utilized in order to investigate the phenotypic traits of all the birds. Results of PCA show that six components of traits characterized 92 percent of total variance. These six components were selected for the discriminate analysis and classification of the studied population based on phenotypic traits.

**Table 2: The classification of highland and lowland chickens based on the discriminate analysis**

|  | True group | |
| --- | --- | --- |
| Put into group | Lowland | Highland |
| Lowland | 8 | 2 |
| Highland | 2 | 4 |
| Total sample | 10 | 6 |
| Correct sample | 8 | 4 |
| Proportion | 0.80 | 0.66 |
| Proportion correct | 0.75 | |

Results of PCA (Table 1) were used for the discriminate analysis. Based on our findings, collected lowland and highland samples can be classified phenotypically into two separated groups. For instance, eight of ten lowland chickens are classified in the lowland chicken group, correctly. Similarly, two of six highland chickens are classified in the highland group. Also, the total accuracy of classification is estimated to be 75%.

**Table 3: The summary of short reads alignments against reference genome for highland and lowland native chickens.**

| Samples | Total reads | Mapped reads% | Reads in Pair% | Coverage before trimming | Coverage after trimming |
| --- | --- | --- | --- | --- | --- |
| Lowland | 72462326 | 96.13 | 92.26 | 9.00 | 8.10 |
| Lowland | 70421388 | 97.00 | 93.53 | 8.70 | 7.88 |
| Lowland | 72850232 | 96.14 | 92.03 | 9.10 | 7.93 |
| Lowland | 62424846 | 96.01 | 92.08 | 7.70 | 6.82 |
| Lowland | 64804044 | 96.59 | 93.01 | 8.10 | 7.27 |
| Highland | 69105254 | 96.54 | 93.07 | 8.60 | 7.55 |
| Highland | 71837220 | 96.91 | 93.61 | 9.00 | 7.96 |
| Highland | 74894416 | 95.34 | 91.45 | 9.30 | 8.08 |
| Highland | 67495174 | 94.35 | 90.75 | 8.40 | 7.26 |
| Highland | 70314734 | 95.58 | 92.41 | 8.70 | 7.73 |

After trimming, the short reads of highland and lowland chickens were mapped against the standard reference genome of chicken (*Gallus gallus*, Ensembl-release 84). Mapping efficiency was estimated between 94% -97% for samples, and the coverage analysis was carried out based on the lander and waterman equation.

**Table 4: Fisher exact test for the identification of differences in variants distribution between highland and lowland samples**

| Variants | P-value |
| --- | --- |
| SNV^***^ | 0.0012 |
| MNV^***^ | 0.0001 |
| Insertion^***^ | 0.0001 |
| Deletion^***^ | 0.0001 |
| Replacement^***^ | 0.0002 |

(P < 0.001) **^***^**

In order to analyze differences of variants distribution, the input file of variants was constructed based on the IDEG6 website instruction and statistical analysis was performed for each variant between highland and lowland samples, separately. Results indicated that variants distributions between highland and lowland were significant, and a possible explanation might also be defined as: differences in variants distribution can be affected by environmental conditions including high- altitude condition.

**Table 5: Chi-square test to analyze the differential distributed variations between highland and lowland chickens for chromosomes (non-significant chromosomes are presented)**

| Variants and chromosomes | | | | | | |
| --- | --- | --- | --- | --- | --- | --- |
| Sex | Male | SNV | MNV | Insertion | Deletion | Replacement |
|  |  | 5,Mt | 9,11,12,18,  19,25,32 | 8,10,13,19  21,25,Mt | 9,10,16,  18,22,25 | 6,9,10,11,12,13,14,15,16,  17,18,20,22,24,26,27,Mt |
|  | Female | Mt | 11,32 | Mt | --- | 3,4,11,W |

Mt: Mitochondria DNA

In addition to the statistical distribution analysis of each variant (Table 4), we analyzed the differential distributed variations between highland and lowland chickens for each chromosome incorporating the sex of birds in this study. We found that the distribution of variants was significant for the most of the chromosomes; therefore, to summarize, non-significant chromosomes were only presented in Table 5.

**Table 6: Classification of differential variants in different types of categories**

| Sex | Total variants | Novel variants | Coding variants | None coding variants | Amino acid changes |
| --- | --- | --- | --- | --- | --- |
| Male | 97610 | 16623 | 614 | 96995 | 202 |
| Female | 17024 | 3024 | 190 | 16834 | 51 |

Numerous differential variants were produced in this investigation (Table S4). Logically, filtration is highly required for the classification of differential variants in order to have a better understanding about their functions by available annotations. For example, known variants annotation was used to identify the novel variants. CDS (coding region) annotation option was selected for the recognition of coding variants in order to find out which variants were located on coding and non-coding regions. Coding variants were collected for functional consequences and amino acid changes analysis. Therefore, reference genome, CDS, and mRNA annotations were used to analyze the amino acid changes. In addition, Standard genetic code was selected in CLC genomics Genomic Workbench (8.5.1) for amino acid change analysis.

Novel variants: variants were reported for the first time.

Coding variants: variants were located in coding regions.

Amino acid changes: variations can change the protein sequence.

| GO | GO term | Description and frequency | Overlapping genes | Chr | Type |
| --- | --- | --- | --- | --- | --- |
| Molecular function | 0004536 | Deoxyribonuclease activity (5%) | *TATDN1 (Asp190Glu)* | 2 | SNV |
|  | 0044822 | Poly (A) RNA binding (9%) | *RARS2 (Gln274Lys)* | 3 | SNV |
|  | 0003785 | Actin monomer binding (5%) | *COBLL1* | 7 | SNV |
|  | 0003887 | DNA-directed DNA polymerase activity (14%) | *POLQ (Thr514Ala)* | 1 | SNV |
|  | 0051575 | 5'-deoxyribose-5phosphate lyase activity (10%) |  |  |  |
|  | 0015410 | Manganese-transporting ATPase activity (9%) | *ATP2C1 (Asp383Glu)* | 2 | SNV |
|  | 0034185 | Apolipoprotein binding (5%) | *LRP6 (Thr522Ser)* | 1 | SNV |
|  | 0005041 | Low-density lipoprotein receptor activity (5%) |  |  |  |
|  | 0035312 | 5'-3' exodeoxyribonuclease activity (14%) | *DCLRE1A* | 6 | SNV |
|  | 0030553 | cGMP binding (10%) | *CNGA3* | 1 | SNV |
|  | 0004582 | Dolichyl - phosphate beta-D-mannosyltransferase activity (14%) | *ALG5* | 1 | SNV |
| Cellular component | 0000799 | Nuclear condensin complex (6%) | *NCAPD3* | 24 | SNV |
|  | 0005901 | Caveola (9%) | *LRP6 (Thr522Ser)* | 1 | SNV |
|  | 0000139 | Golgi membrane (11%) | *GOSR1* | 19 | SNV |
|  | 0005801 | Cis-Golgi network (6%) |  |  |  |
|  | 0005887 | Integral to plasma membrane (29%) | *SLC38A6* | 5 | MNV |
|  | 0070419 | Nonhomologous end joining complex (9%) | *PRKDC* | 2 | SNV |
|  | 0005654 | Nucleoplasm (12%) |  |  |  |
|  | 0000784 | Nuclear chromosome, Telomeric region (6%) | *DCLRE1A* | 6 | SNV |
|  | 0030686 | 90S preribosome (6%) | *UTP20* | 1 | SNV |
|  | 0070382 | Exocytic vesicle (6%) | *SYTL2* | 1 | SNV |
| Biological process | 0006303 | Double-strand break repair via nonhomologous end joining (4%) | *DCLRE1A* | 6 | SNV |
|  | 0006874 | Cellular calcium ion homeostasis (12%) | *ATP13A4 (lu589Asp)* |  |  |
|  | 0003344 | Pericardium morphogenesis (6%) | *LRP6 (Thr522Ser)* | 1 | SNV |
|  | 0021794 | Thalamus development (5%) |  |  |  |
|  | 0030901 | Midbrain development (6%) |  |  |  |
|  | 0021987 | Cerebral cortex development (7%) |  |  |  |
|  | 0060059 | Embryonic retina morphogenesis in camera-type eye (4%) |  |  |  |
|  | 0060325 | Face morphogenesis (1%) |  |  |  |
|  | 0051639 | Actin filament network formation (1%) | *COBLL1* | 7 | SNV |
|  | 0071897 | DNA biosynthetic process (1%) | *POLQ* | 1 | SNV |
|  | 0006302 | Double-strand break repair (18%) |  |  |  |
|  | 2000042 | Negative regulation of double-strand break repair via homologous recombination (4%) |  |  |  |
|  | 0001865 | NK T cell differentiation (2%) | *BMX (Arg122Lys)* | 1 | SNV |
|  | 0006281 | DNA repair (25%) | *PRKDC* | 2 | SNV |
|  | 0036297 | Inter strand cross-link repair (4%) | *DCLRE1A* | 6 | SNV |

**Table 7: Gene ontology enrichments analysis for differential variants – Male birds**

**Table 8: Gene ontology enrichments analysis for differential variants – Female birds**

| GO | Go term | Description and frequency | Overlapping genes | Chr | Type |
| --- | --- | --- | --- | --- | --- |
| Molecular function | 0005198 | Structural molecule activity (7%) | *PSMD13 (Ile118Thr)* | 5 | SNV |
|  | 0035312 | 5'-3' exodeoxyribonuclease activity (14%) | *DCLRE1C* | 1 | Deletion |
|  | 0005049 | Nuclear export signal receptor activity (3%) | *RANBP17* | 13 | SNV |
|  | 0008536 | Ran GTPase binding (7%) |  |  |  |
|  | 0042393 | Histone binding (13%) | *ATAD2* | 2 | SNV |
|  | 0008453 | Alanine-glyoxylate transaminase activity (14%) | *AGXT2 (Ala436Gly)* | Z | SNV |
|  | 0030170 | Pyridoxal phosphate binding (12%) |  |  |  |
|  | 0004222 | Metalloendopeptidase activity (9%) | *UQCRC1 (Arg211Gln)* | 12 | SNV |
|  | 0050839 | Cell adhesion molecule binding (8%) | *CD200 (His135Asn)* | 1 | SNV |
|  | 0004872 | Receptor activity (13%) |  |  |  |
| Cellular component | 0070545 | PeBoW complex (3%) | *PES1* | 15 | Deletion |
|  | 0005750 | Mitochondrial respiratory chain complex III (9%) | *UQCRC1 (Arg211Gln)* | 12 | SNV |
|  | 0022624 | Proteasome accessory complex (9%) | *PSMD13 (Ile118Thr)* | 5 | SNV |
|  | 0008541 | Proteasome regulatory particle (6%) |  |  |  |
|  | 0070419 | Nonhomologous end joining complex (12%) | *DCLRE1C* | 1 | Deletion |
|  | 0000784 | Nuclear chromosome, telomeric region (13%) |  |  |  |
|  | 0005769  0031410 | Early endosome (2%)  Cytoplasmic vesicle (17%) | *ZFYVE9* | 8 | SNV |
|  | 0005840 | Ribosome (12%) | *HSPA14 (Leu397Va)* | 1 | SNV |
|  | 0005913 | Cell-cell adherens junction (17%) | *CD200 (His135Asn)* | 1 | SNV |
| Biological process | 0006835 | Dicarboxylic acid transport (3%) | *SLC13A2* | 19 | SNV |
|  | 0000463 | Maturation of LSU-rRNA from tricistronic rRNA transcript (SSU-rRNA, 5.8S rRNA, LSU-rRNA) (7%) | *PES1* | 15 | Deletion |
|  | 0006611 | Protein export from nucleus (8%) | *RANBP17* | 13 | SNV |
|  | 0006303 | Double-strand break repair via non-homologous end joining (9%) | *DCLRE1C* | 1 | Deletion |
|  | 0036297 | Inter strand cross-link repair (3%) |  |  |  |
|  | 0031848 | Protection from non-homologous end joining at telomere (4%) |  |  |  |
|  | 0090305 | Nucleic acid phosphodiester bond hydrolysis (15%) |  |  |  |
|  | 0007156 | Homophilic cell adhesion (8%) | *CD200 His135Asn* | 1 | SNV |
|  | 0007157 | Heterophilic cell-cell adhesion (8%) |  |  |  |
|  | 0008037 | Cell recognition (5%) |  |  |  |
|  | 0006511 | Ubiquitin-dependent protein catabolic process (15%) | *PSMD13 Ile118Thr* | 5 | SNV |
|  | 0043248 | Proteasome assembly (3%) |  |  |  |
|  | 0009060 | Aerobic respiration (9%) | *UQCRC1 (Arg211Gln)* | 12 | SNV |
|  | 0006122 | Mitochondrial electron transport, ubiquinol to cytochrome c (3%) |  |  |  |

Results of the GO analysis are given in Tables 7 and 8. The output of amino acid change analysis for differential variants was used for gene ontology enrichment analyses in three parts, including biological process, molecular function and cellular component. The gene ontology (GO) association file, which includes gene names and associated gene ontology terms, were downloaded from the gene ontology consortium (http://geneontology.org/) and imported to CLC Genomic Workbench (8.5.1). The significance level of GO analysis was also determined as 0.01

**Table 9: Classification of mtDNA variation in highland and lowland chickens**

| Population | SNV | Insertion | Replacement | Total | New variants | Coding variants | Amino acid changes |
| --- | --- | --- | --- | --- | --- | --- | --- |
| Highland-male | 21 | 1 | 1 | 23 | 23 | 14 | 5 |
| Highland-female | 9 | 0 | 0 | 9 | 8 | 4 | 1 |
| Lowland-male | 8 | 0 | 0 | 8 | 8 | 6 | 2 |
| Lowland-female | 10 | 0 | 0 | 10 | 10 | 7 | 2 |

In this study, mtDNA variants were analyzed, separately. After variants calling, all mtDNA variants including SNV, insertion and replacement were obtained from highland and lowland chickens, separately. Filtrations were also carried out same as what described for differential variants. We recognized that highland chickens had more variants than lowland chickens (32 versus 18 variants). It might be explained that more variants in highland chickens were created in order to adapt to the high-altitude conditions.

Novel variants: variants were reported for the first time.

Coding variants: variants were located on coding regions.

Amino acid changes: The protein sequence can be changed by the variations.

**Table 10: Validation of novel differential SNVs and mtDNA variants in highland chickens**

| Chr | Region | Genotype | Validation % | Variant type |
| --- | --- | --- | --- | --- |
| MtDNA | 164 | C/T | 20 | Single nucleotide variant (SNV) |
|  | 307 | C/T | 20 |  |
|  | 443 | C/T | 20 |  |
|  | **5928** | C/A | **40** |  |
|  | **6758** | T/C | **40** |  |
|  | 6800 | T/C | 20 |  |
|  | 7530 | C/G | 20 |  |
|  | **8070** | T/C | **40** |  |
|  | 9533 | A/G | 20 |  |
|  | 10072 | A/G | 20 |  |
|  | 11378 | C/T | **40** |  |
|  | 11963 | C/T | 20 |  |
|  | 16586 | A/G | 20 |  |
|  | 253 | T/C | 20 |  |
|  | 258 | C/T | 20 |  |
|  | 4580 | G/A | 20 |  |
|  | **8330** | T/C | **40** |  |
|  | **11378** | C/T | **40** |  |
|  | 12094 | T/C | 20 |  |
| 1 | 1254679 | A/G | 20 |  |
| **1** | **2704537** | **G/A** | **40** |  |
| 1 | 103830963 | C/T | 20 |  |
| **1** | **2704537** | **G/A** | **60** |  |
| 1 | 165925917 | G/T | 20 |  |
| 1 | 89054158 | C/T | 20 |  |
| 1 | 180719232 | A/G | 20 |  |
| 1 | 138527429 | G/A | 20 |  |
| **1** | **2704537** | **G/A** | **40** |  |
| 2 | 4877832 | C/T | 20 |  |
| 2 | 56043229 | C/T | 20 |  |
| 2 | 49059638 | T/C | 20 |  |
| 3 | 33701810 | A/G | 20 |  |
| 3 | 65865344 | A/C | 20 |  |
| 4 | 76983077 | A/G | 20 |  |
| 6 | 2014167 | A/C | 20 |  |
| 7 | 17112008 | C/G | 20 |  |
| 8 | 8248431 | A/T | 20 |  |
| 9 | 2581777 | C/T | 20 |  |
| 12 | 816375 | A/G | 20 |  |
| 17 | 8030611 | G/A | 20 |  |

Validations were performed for two groups of detected variants in this study. Frist, novel differential SNVs between highland and lowland chickens, and second, mtDNA variations in highland chickens. Thus, other five whole genomes of highland samples (Isfahan) were sequenced, separately. The whole genome sequencing, trimming and variant detection were carried out based on provided information in material and method sections. A total of 3845 SNVs was reported as new variants for differential variants analyses, and 32 variations were detected in mtDNA for highland samples. The region and chromosomes of each SNV were evaluated in the new five samples by R program ([www.R-project.org](http://www.R-project.org)) in order to validate the variants. The calculation of validation’s percentage was based on samples that had variants. Finally, variants of 19 and 21 were validated for mtDNA and novel differential variants, respectively.

| Sex | Gene ID | Biotype | Function | Product name | Gene name | Uniprot ID |
| --- | --- | --- | --- | --- | --- | --- |
| Female  Male | ENSGALG00000028220 | Protein coding | structural constituent of cytoskeleton | Keratin | *LOC107055272* | E1C3X3 |
|  | ENSGALG00000026257 | Protein coding | structural constituent of cytoskeleton | Keratin | *LOC107055272* | E1BXQ5 |
|  | ENSGALG00000028852 | miRNA | Gastrulation | MIR6644-2 | *MIR6644-2* | No protein |
|  | ENSGALG00000026447 | Protein coding | Cytoskeleton organization | Keratin | *LOC428303* | E1C3N8 |
|  | ENSGALG00000027740 | miRNA | Unknown | Unknown | Unknown | No protein |
|  | ENSGALG00000011733 | Protein coding | immune response | C-C chemokine receptor type 2 | *CCR2* | F1NPK8 |
|  | ENSGALG00000011734 | Protein coding | chemotaxis | C-C chemokine receptor 8 like | *CCR8L* | F1NPK7 |
|  | ENSGALG00000011735 | Protein coding | chemokine receptor activity | chemokine XC receptor 1 | *CCXCR1-L* | Q702H5 |
|  | ENSGALG00000011808 | Protein coding | macrophage receptor activity | C-C chemokine receptor type 9 | *CCR9* | F1NMB1 |
|  | ENSGALG00000012671 | Protein coding | Hormone activity | Prolactin | *Prl* | A0A0B5L5F3 |
|  | ENSGALG00000012700 | Protein coding | ubiquitin-protein transferase activity | Ring finger protein 182 | *RNF182* | E1BZ35 |
|  | ENSGALG00000012702 | Protein coding | Chromatin binding | Protein Jumonji | *JARID2* | F1NWZ0 |
|  | ENSGALG00000012732 | Protein coding | protein phosphatase regulator activity | Phosphatase and actin regulator | *PHACTR1* | F1NWB1 |
|  | ENSGALG00000012748 | Protein coding | Elongation of very long chain fatty acids protein 2 | fatty acid elongase activity | *ELOVL2* | E1BYE9 |

**Table 11: Genomic variants enrichment analysis for the identification of candidate gene associated with hypoxia in males and females**

Identification of the genomic regions, which had the highest density of differential variant between highland and lowland chickens, is considered as the main purpose of genomic variants enrichment analysis. The candidate genes located in the regions of genome, which had the highest density of variants, can be observed in this table. We found that the most important functions that can be considered in low oxygen conditions are Immune response, chromatin binding, and gastrulation. In addition, MIR6644-2 was reported as the candidate gene in order to develop the gastrulation process, and was not implicated in the previous investigations about high-altitude adaptation of chickens.


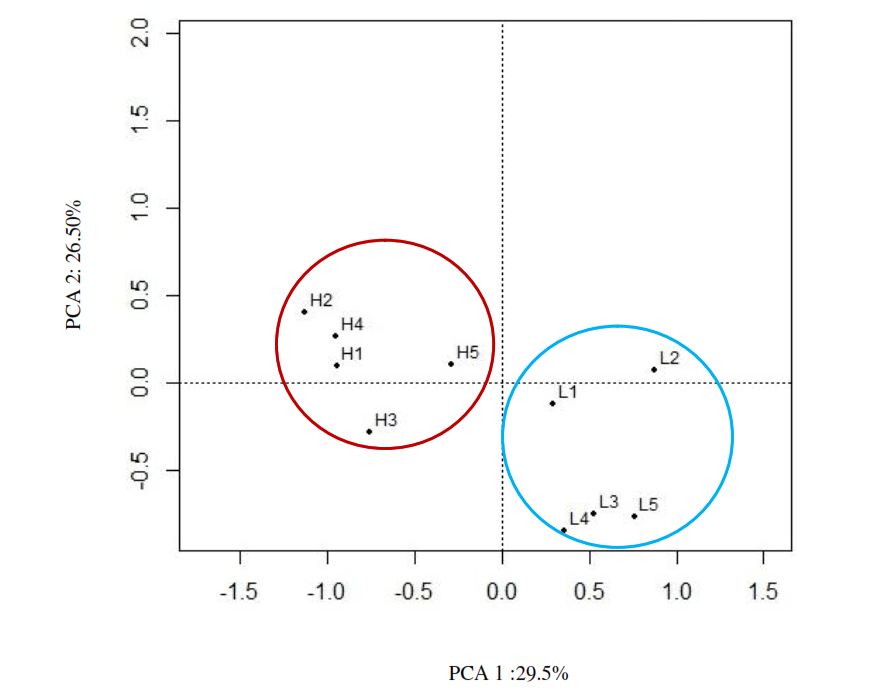


**Figure 1: Classification of the highland and lowland chickens according to the results of multiple corresponding analyses (H: highland and L: lowland).**

The information of 10000 SNVs genotypes were utilized in order to classify the highland and lowland chickens based on multiple corresponding analysis by R program and Ca package. Results show that highland and lowland chickens can be divided into two groups based on their geographical distribution.

**Figure 2: Standard frequency of differential variants between highland and lowland chicken for the selection of chromosomes that have the highest standard frequency**

Dividing variants numbers by the chromosome length (Mb), standard frequencies of differential variants were estimated for each chromosome, then chromosomes with high-density of variants were selected (marked with asterisk) in order to be utilized for further analysis.


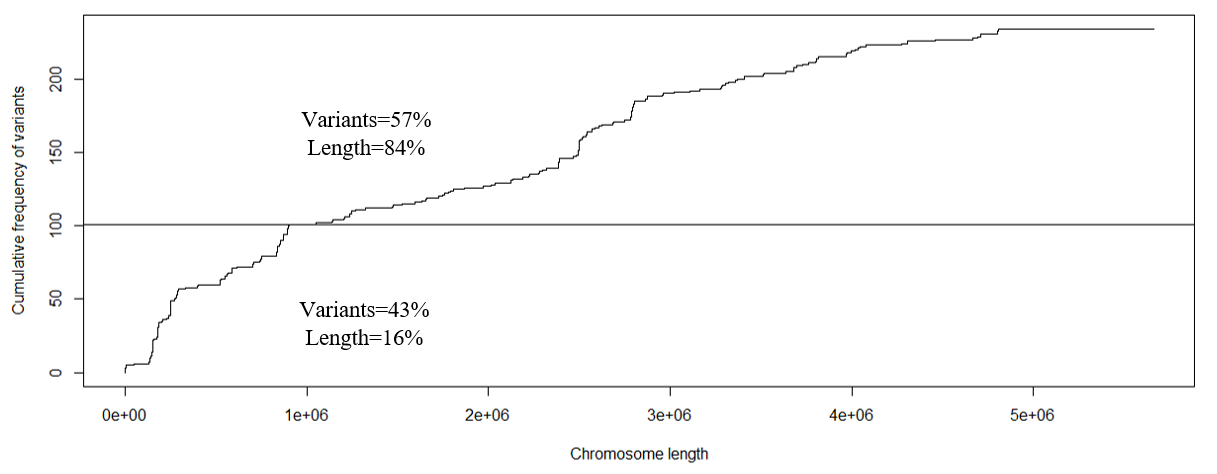


**(a)**


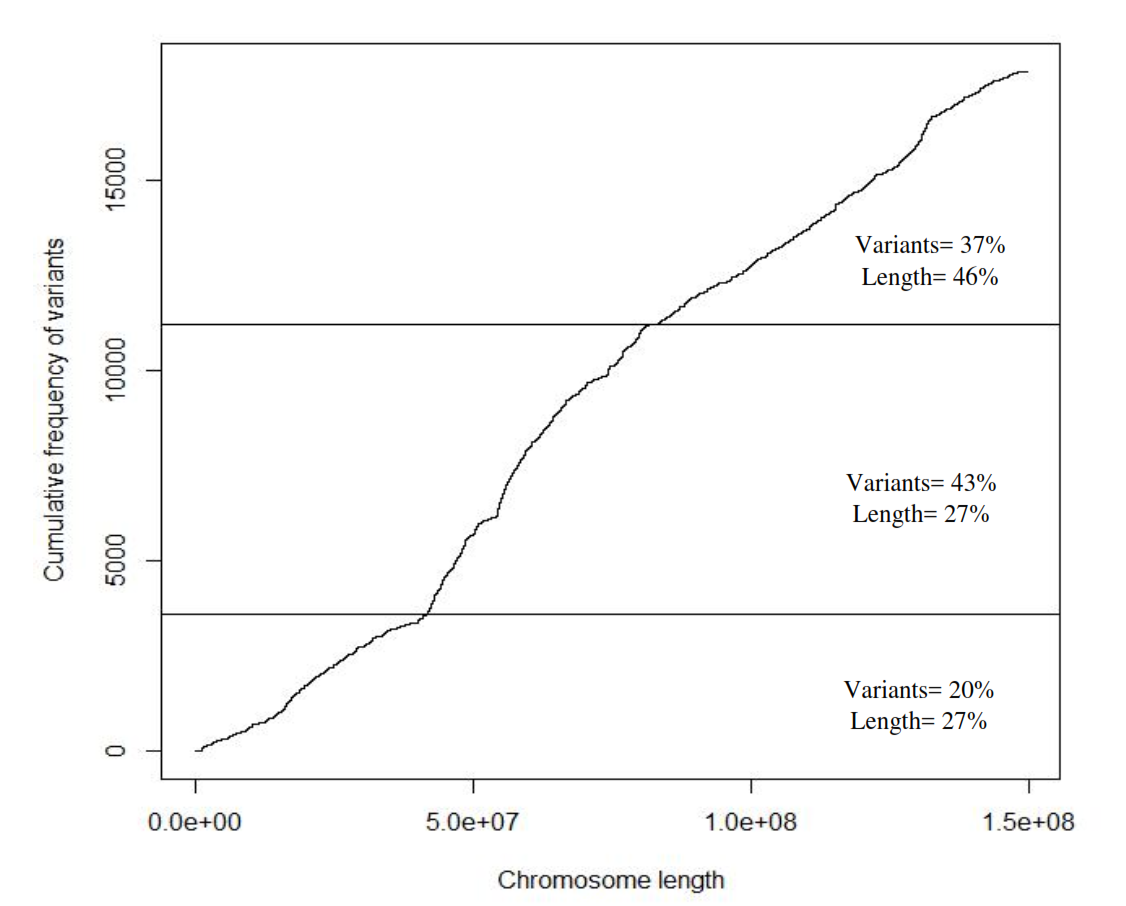


Variants=57%

Length=84%

**(b)**

**Figure 3: Cumulative frequency graph of variant numbers for chromosomes 27 (Figure a) and 2 (Figure b) in female and male chickens for genomic variant enrichment analysis.**

Chromosomes 2 and 27 (Fig 2) were selected in order to draw the cumulative frequencies graph of variants along the chromosome. It is shown by Figure (a) that the most variants enrichments are available in the range of 2692-1×10^6^ base pairs (separated with horizontal line) of chromosome 27. A total of 234 variants was recognized on chromosome 27, and 100 (42.72%) variants were also located in the range of 2692-1×10^6^ region. Figure (b) indicates that 17891 variants were recognized on chromosome 2, but 7600 variants (43%) were located in the range of 41×10^6^-81×10^6^(27% of chromosome length) base pairs. Finally, these regions with the highest density of variation were selected for the identification of candidate genes.


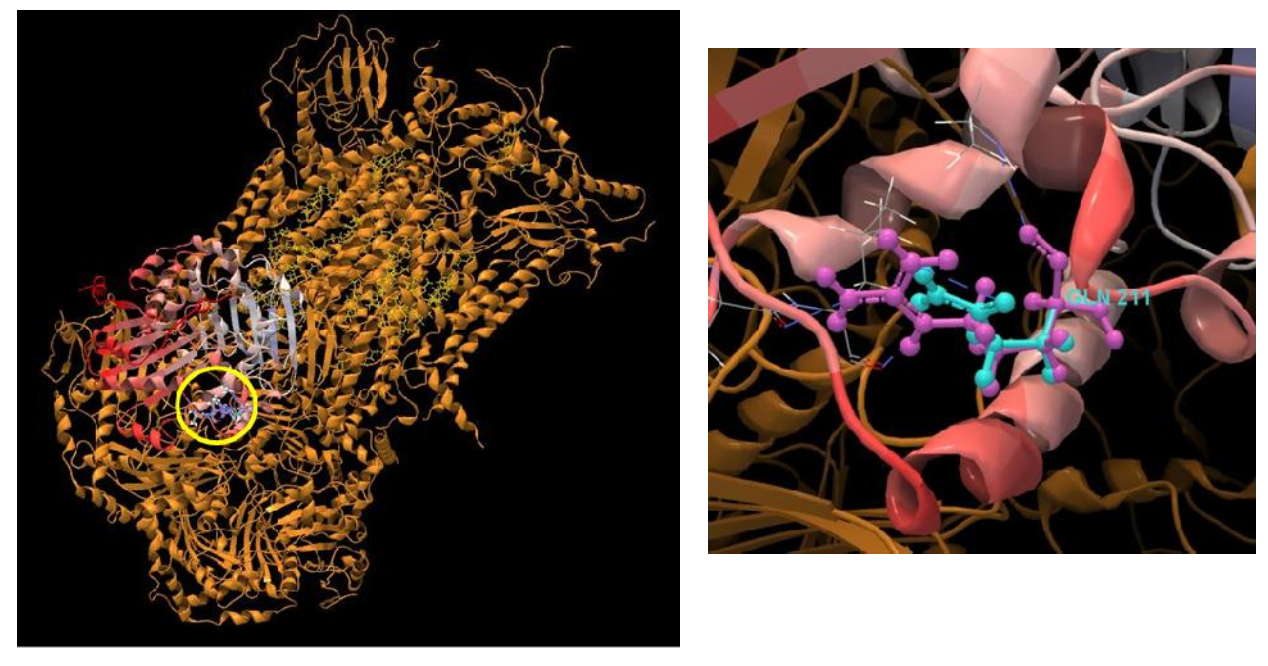


***UQCRC1 (Arg211Gln)***


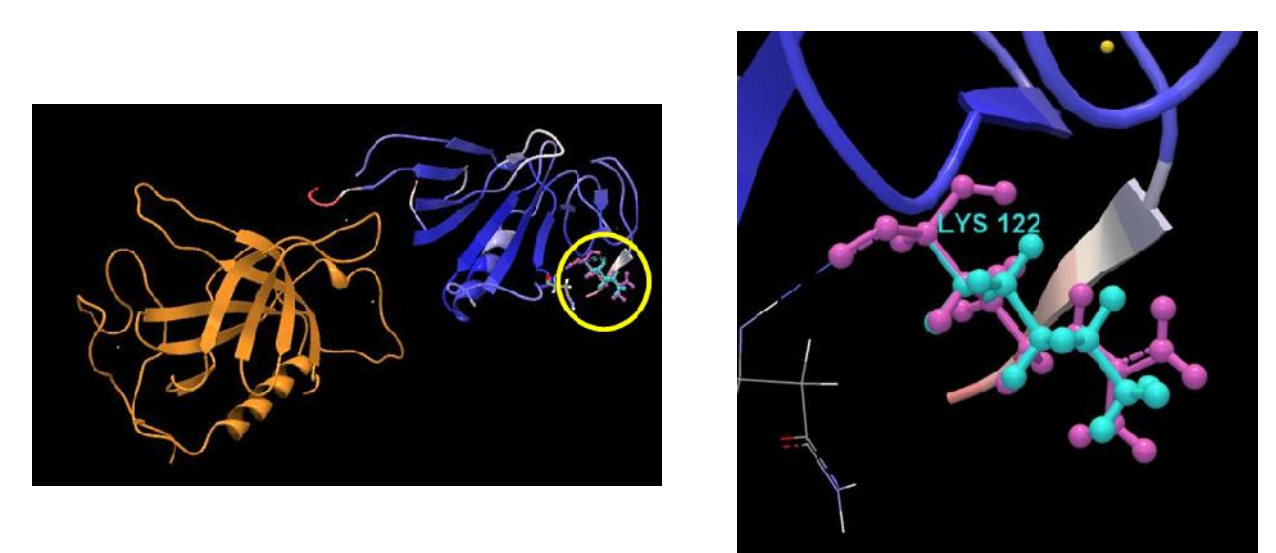
**Figure 4:** *UQCRC1* gene encodes ubiquinol cytochrome c reductase or cytochrome bc1 complex protein. It is a complex protein which is located on the mitochondrial inner membrane and involves in the cell respiration. The variants locations are shown by the yellow circle in the whole structure. Purple and green atoms show the reference and variants structures, respectively.

***BMX (Arg 122 Lys)***

**Figure 5:** *BMX* gene encodes cytoplasmic tyrosine-protein kinase and contributes in the NK T cell differentiation. It plays a critical role in the induced interleukin-6 (IL6), adaptation of different cell systems against stress, growth, and differentiation of hematopoietic cell. Therefore, *BMX* gene was considered as the immune response in hypoxia conditions. The variants locations are shown by the yellow circle in the whole structure. Purple and green atoms also show the reference and variants structures, respectively.


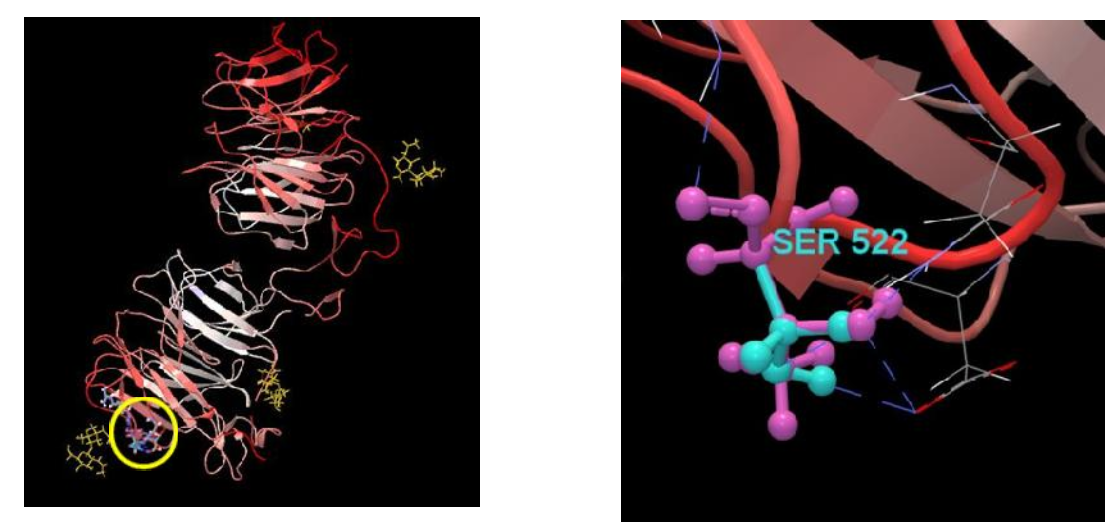


***LRP6* (*Thr522Ser*)**

**Figure 6:** *LRP6* gene was considered as a candidate gene for organs development in high altitude conditions. *LRP6* gene encodes low-density lipoprotein receptor-related protein 6 and is a key regulator of tissue development and hemostasis. The yellow circle in the whole structure shows variants locations. The reference and variants structures are also shown by purple and green atoms, respectively.


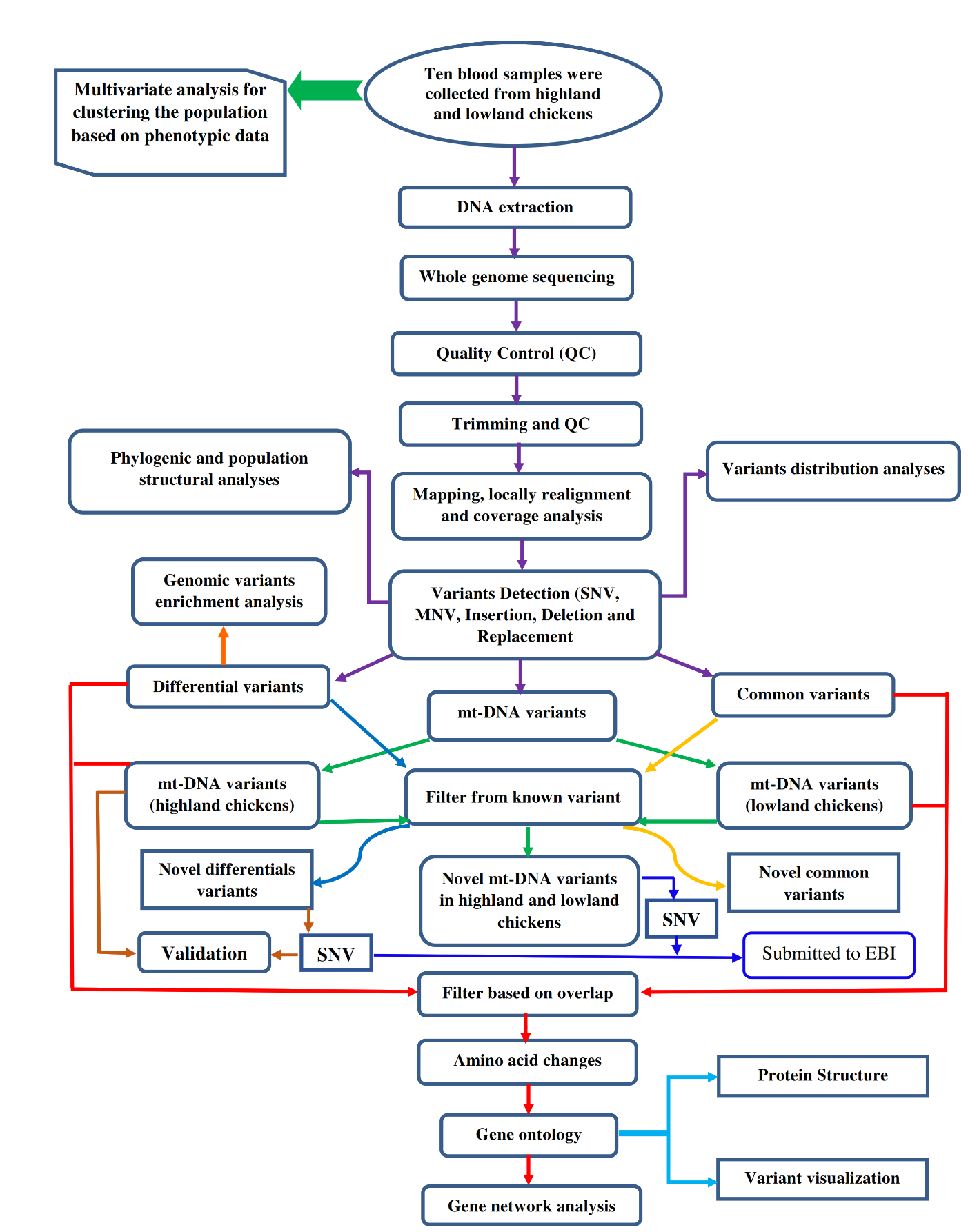


**Figure 7:** The Summary of sampling and data analysis for whole-genome sequencing of native chicken ecotypes and variant discovery associated with high-altitude adaptation (the same colors of vectors show the similar analysis).

**
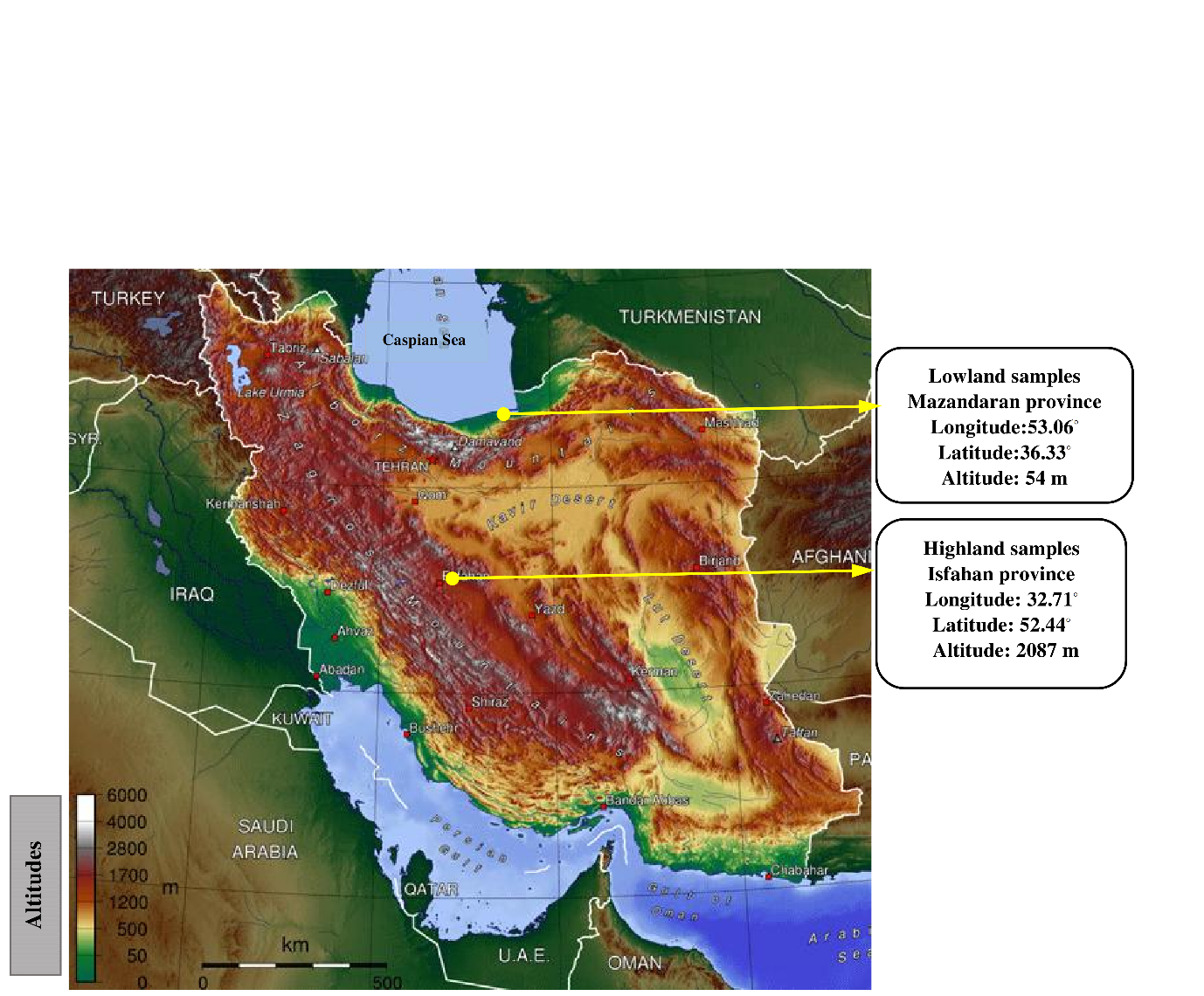
**

**Figure 8: Sampling locations of highland and lowland chickens in Isfahan and Mazandaran provinces, located at different altitudes in Iran.**

Ten blood samples, including three males and two females from highland and two males and three females from lowland, were collected in order to be utilized in the whole genome sequencing.

This figure was downloaded from an open source website <https://commons.wikimedia.org>.

The direct URL is <https://upload.wikimedia.org/wikipedia/commons/9/99/Iran_topo_en.jpg>.

The link to the license is <https://creativecommons.org/licenses/by-sa/4.0/deed.en>.

Outer areas from the original figure were cropped, therefore “Caspian Sea” was rewritten in the above of figure based on the information of original figure. In addition, the locations of sampling were added to the figure by yellow vectors.

**
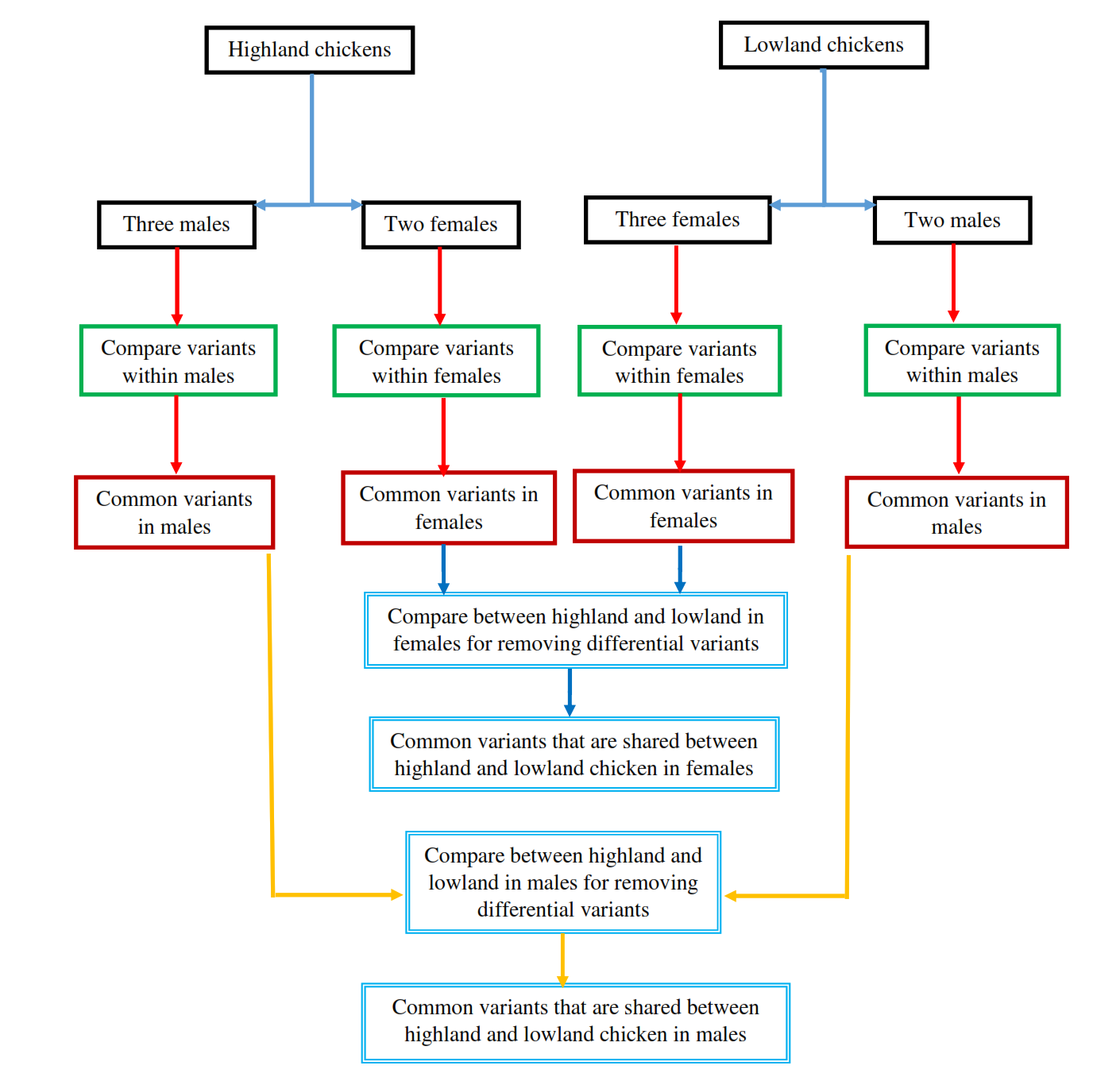
**

**Figure 9: The process of common variants detection in highland and lowland native chicken ecotypes**.

This figure shows that how differential variants were removed between highland and lowland chickens. All comparisons of native chicken ecotype were carried out based on the sex of birds in each step. The frequency threshold was determined to be 100 percent in order to collect common variants between highland and lowland chickens.
