# Supplementary material for: Gene network analysis to determine the effect of hypoxia-associated genes on brain damages and tumorigenesis using an avian model
Source: J Genet Eng Biotechnol. 2021 Jul 8;19:100. doi: 10.1186/s43141-021-00184-5 (PMC8266987; doi:10.1186/s43141-021-00184-5)
Supplement: Supplementary file 2 — The list of selected hypoxia- associated genes for gene network analysis. Table S2: Principle component analysis of phenotypic traits for discriminate analysis and clustering the highland and lowland populations. Table S3: The classification of highland and lowland chickens based on the discriminate analysis. [file 43141_2021_184_MOESM2_ESM.docx]

**Table S1: The list of selected hypoxia- associated genes for gene network analysis**

| Number | Gene name | Chicken Ensembl ID | Human’s orthologs ID |
| --- | --- | --- | --- |
| 1 | AKAP12 | ENSGALG00000036240 | ENSG00000131016 |
| 2 | AKT1 | ENSGALG00000011620 | ENSG00000142208 |
| 3 | ALPK3 | ENSGALG00000037950 | ENSG00000136383 |
| 4 | APOB | ENSGALG00000016491 | ENSG00000084674 |
| 5 | ARG2 | ENSGALG00000009519 | ENSG00000081181 |
| 6 | ATAD2 | ENSGALG00000034348 | ENSG00000156802 |
| 7 | ATR | ENSGALG00000002663 | ENSG00000175054 |
| 8 | BAG1 | ENSGALG00000013157 | ENSG00000107262 |
| 9 | BARD1 | ENSGALG00000003484 | ENSG00000138376 |
| 10 | BCL2 | ENSGALG00000012885 | ENSG00000171791 |
| 11 | BDKRB2 | ENSGALG00000011080 | ENSG00000168398 |
| 12 | BGLAP | ENSGALG00000029494 | ENSG00000242252 |
| 13 | BID | ENSGALG00000013039 | ENSG00000015475 |
| 14 | BMX | ENSGALG00000016557 | ENSG00000102010 |
| 15 | CA4 | ENSGALG00000005360 | ENSG00000167434 |
| 16 | CASP3 | ENSGALG00000010638 | ENSG00000164305 |
| 17 | CCDC170 | ENSGALG00000012971 | ENSG00000120262 |
| 18 | CD109 | ENSGALG00000015910 | ENSG00000156535 |
| 19 | CD200 | ENSGALG00000015396 | ENSG00000091972 |
| 20 | CENPF | ENSGALG00000009690 | ENSG00000117724 |
| 21 | CSF1 | ENSGALG00000033854 | ENSG00000184371 |
| 22 | CTNNB1 | ENSGALG00000037203 | ENSG00000168036 |
| 23 | CYCS | ENSGALG00000011020 | ENSG00000172115 |
| 24 | DDAH1 | ENSGALG00000008663 | ENSG00000153904 |
| 25 | DKK1 | ENSGALG00000003814 | ENSG00000107984 |
| 26 | DNM1 | ENSGALG00000004852 | ENSG00000106976 |
| 27 | DNM1L | ENSGALG00000012929 | ENSG00000087470 |
| 28 | DOT1L | ENSGALG00000000843 | ENSG00000104885 |
| 29 | DVL1 | ENSGALG00000001600 | ENSG00000107404 |
| 30 | EP300 | ENSGALG00000011992 | ENSG00000100393 |
| 31 | GAS8 | ENSGALG00000000540 | ENSG00000141013 |
| 32 | GPNMB | ENSGALG00000010949 | ENSG00000136235 |
| 33 | GPX1 | ENSGALG00000028204 | ENSG00000233276 |
| 34 | GSK3B | ENSGALG00000014977 | ENSG00000082701 |
| 35 | HDAC10 | ENSGALG00000041251 | ENSG00000100429 |
| 36 | HIF1A | ENSGALG00000011870 | ENSG00000100644 |
| 37 | IRS1 | ENSGALG00000004959 | ENSG00000169047 |
| 38 | IRS2 | ENSGALG00000026287 | ENSG00000185950 |
| 39 | LRP1B | ENSGALG00000012407 | ENSG00000168702 |
| 40 | LRP5 | ENSGALG00000029533 | ENSG00000162337 |
| 41 | LRP6 | ENSGALG00000031503 | ENSG00000070018 |
| 42 | LRPPRC | ENSGALG00000009967 | ENSG00000138095 |
| 43 | MAPT | ENSGALG00000000625 | ENSG00000186868 |
| 44 | MCU | ENSGALG00000004355 | ENSG00000156026 |
| 45 | MICU1 | ENSGALG00000004372 | ENSG00000107745 |
| 46 | NOS1 | ENSGALG00000008177 | ENSG00000089250 |

**Table S1 Continued**

| Number | Gene names | Chickens Ensembl IDs | Human’s orthologs IDs |
| --- | --- | --- | --- |
| 47 | NOS2 | ENSGALG00000038096 | ENSG00000007171 |
| 48 | NQO1 | ENSGALG00000023437 | ENSG00000181019 |
| 49 | NR3C1 | ENSGALG00000032992 | ENSG00000113580 |
| 50 | OGDH | ENSGALG00000014155 | ENSG00000105953 |
| 51 | OPA1 | ENSGALG00000007150 | ENSG00000198836 |
| 52 | PARP1 | ENSGALG00000009228 | ENSG00000143799 |
| 53 | PDE5A | ENSGALG00000011982 | ENSG00000138735 |
| 54 | PDPK1 | ENSGALG00000006418 | ENSG00000140992 |
| 55 | POLQ | ENSGALG00000014924 | ENSG00000051341 |
| 56 | PRKDC | ENSGALG00000042469 | ENSG00000253729 |
| 57 | RHOA | ENSGALG00000003806 | ENSG00000067560 |
| 58 | RIF1 | ENSGALG00000012484 | ENSG00000080345 |
| 59 | RUNX2 | ENSGALG00000040276 | ENSG00000124813 |
| 60 | SAMD3 | ENSGALG00000032409 | ENSG00000164483 |
| 61 | SGK1 | ENSGALG00000013971 | ENSG00000118515 |
| 62 | SIRT2 | ENSGALG00000029761 | ENSG00000068903 |
| 63 | SLC18A2 | ENSGALG00000009289 | ENSG00000165646 |
| 64 | SLC38A6 | ENSGALG00000011893 | ENSG00000139974 |
| 65 | SLC8A1 | ENSGALG00000008544 | ENSG00000183023 |
| 66 | SLC8B1 | ENSGALG00000008337 | ENSG00000089060 |
| 67 | SLC9A1 | ENSGALG00000034251 | ENSG00000090020 |
| 68 | SLX4 | ENSGALG00000031841 | ENSG00000188827 |
| 69 | SOD2 | ENSGALG00000011661 | ENSG00000285441 |
| 70 | SOST | ENSGALG00000009929 | ENSG00000167941 |
| 71 | SYNE2 | ENSGALG00000011811 | ENSG00000054654 |
| 72 | TBC1D4 | ENSGALG00000016925 | ENSG00000136111 |
| 73 | TGFBR1 | ENSGALG00000033179 | ENSG00000106799 |
| 74 | TNFRSF11B | ENSGALG00000043167 | ENSG00000164761 |
| 75 | TYR | ENSGALG00000017237 | ENSG00000077498 |
| 76 | UIMC1 | ENSGALG00000001158 | ENSG00000087206 |
| 77 | USP1 | ENSGALG00000010957 | ENSG00000162607 |
| 78 | VEGFC | ENSGALG00000010847 | ENSG00000150630 |
| 79 | XDH | ENSGALG00000008701 | ENSG00000158125 |
| 80 | XIRP1 | ENSGALG00000006025 | ENSG00000168334 |

**Table S2: Principle component analysis of phenotypic traits for discriminate analysis and clustering the highland and lowland populations**

| Component | 1 | 2 | 3 | 4 | 5 | 6 |
| --- | --- | --- | --- | --- | --- | --- |
| Eigenvalue | 6.45 | 2.64 | 2.07 | 1.14 | 0.79 | 0.69 |
| Proportion | 0.43 | 0.17 | 0.13 | 0.07 | 0.05 | 0.04 |
| Cumulative | 0.43 | 0.60 | 0.74 | 0.82 | 0.87 | **0.92** |

Twenty-four quantitative traits were measured on 16 highland and lowland chickens by our investigation. In order to reduce the measurement error, all chickens were mature while recording and the same recording protocol was also utilized in order to investigate the phenotypic traits of all the birds. Results of PCA show that six components of traits characterized 92 percent of total variance. These six components were selected for the discriminate analysis and classification of the studied population based on phenotypic traits.

**Table S3: The classification of highland and lowland chickens based on the discriminate analysis**

|  | True group | |
| --- | --- | --- |
| Put into group | Lowland | Highland |
| Lowland | 8 | 2 |
| Highland | 2 | 4 |
| Total sample | 10 | 6 |
| Correct sample | 8 | 4 |
| Proportion | 0.80 | 0.66 |
| Proportion correct | 0.75 | |

Results of PCA (Table 1) were used for the discriminate analysis. Based on our findings, collected lowland and highland samples can be classified phenotypically into two separated groups. For instance, eight of ten lowland chickens are classified in the lowland chicken group, correctly. Similarly, two of six highland chickens are classified in the highland group. Also, the total accuracy of classification is estimated to be 75%.
